# Ionic Liquid-Based Surfactants: Recent Advances in Their Syntheses, Solution Properties, and Applications

**DOI:** 10.3390/polym13071100

**Published:** 2021-03-30

**Authors:** Omar A. El Seoud, Nicolas Keppeler, Naved I. Malek, Paula D. Galgano

**Affiliations:** 1 Institute of Chemistry, The University of São Paulo, São Paulo 05508-000, Brazil; nicolas.keppeler@usp.br (N.K.); paula.galgano@gmail.com (P.D.G.); 2 Applied Chemistry Department, Sardar Vallabhbhai National Institute of Technology, Surat 395 007, Gujarat, India; navedmalek@gmail.com

**Keywords:** ionic liquids, ionic liquid-based surfactants, gemini ionic liquid-based surfactants, adsorption at water/air interface, formation of micelles and microemulsions, molecular structure/properties relationships, mesoporous nanoparticles, catalysis, drug delivery, polymerization

## Abstract

The impetus for the expanding interest in ionic liquids (ILs) is their favorable properties and important applications. Ionic liquid-based surfactants (ILBSs) carry long-chain hydrophobic tails. Two or more molecules of ILBSs can be joined by covalent bonds leading, e.g., to gemini compounds (GILBSs). This review article focuses on aspects of the chemistry and applications of ILBSs and GILBSs, especially in the last ten years. Data on their adsorption at the interface and micelle formation are relevant for the applications of these surfactants. Therefore, we collected data for 152 ILBSs and 11 biamphiphilic compounds. The head ions of ILBSs are usually heterocyclic (imidazolium, pyridinium, pyrrolidinium, etc.). Most of these head-ions are also present in the reported 53 GILBSs. Where possible, we correlate the adsorption/micellar properties of the surfactants with their molecular structures, in particular, the number of carbon atoms present in the hydrocarbon “tail”. The use of ILBSs as templates for the fabrication of mesoporous nanoparticles enables better control of particle porosity and size, hence increasing their usefulness. ILs and ILBSs form thermodynamically stable water/oil and oil/water microemulsions. These were employed as templates for (radical) polymerization reactions, where the monomer is the “oil” component. The formed polymer nanoparticles can be further stabilized against aggregation by using a functionalized ILBS that is co-polymerized with the monomers. In addition to updating the literature on the subject, we hope that this review highlights the versatility and hence the potential applications of these classes of surfactants in several fields, including synthesis, catalysis, polymers, decontamination, and drug delivery.

*Note: Abbreviations and acronyms are listed after Acknowledgments*

## 1. Introduction

**Figure 1:**
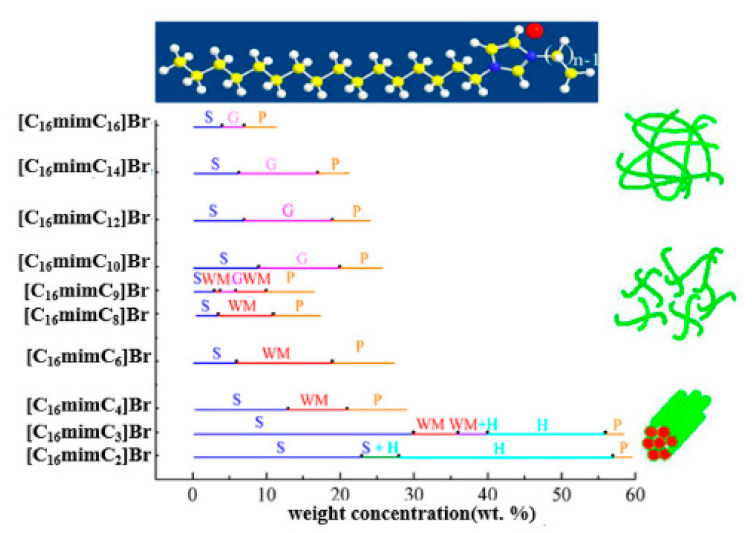
Formation of different morphologies as a function of the surfactant molecular structure (1-C_16_-3-R-imidazolium bromides; R = C_2_ to C_16_) and their concentration in water. S, H, WM, G and P refer to isotropic solution, hexagonal liquid crystals, wormlike micelles, hydrogel and ionic liquid-based surfactants precipitation, respectively [2]. Reprinted with permission from ref. [2]. Copright 2021 Elsevier.

Ionic liquids (ILs) are electrolytes whose melting points are, by operational definition, ≤100 °C. Ionic liquid-based surfactants (ILBSs) are ILs that carry hydrophobic “tails” and hence form colloidal aggregates in water, e.g., micelle and vesicles. Single-chain ILBSs can be covalently linked to form dimers (so-called gemini surfactants, GILBSs), trimers and, eventually, polymeric ILBSs. This structure versatility can be exploited to obtain different structures as shown in Figure S1 (in Supplementary Material) [1], and to obtain several colloidal morphologies, as can be seen in Figure 1 for a series of 1-hexadecyl-3-R-imidazolium bromides, allowing potentially interesting applications. Thus, the increase in the length of R from C_2_ to C_16_ leads to changes from isotropic solution to worm-like micelles, hexagonal liquid crystals, hydrogel and, eventually, surfactant precipitation [2]. Additionally, these surfactants also form thermodynamically stable water-in-oil (W/O) and oil-in-water (O/W) microemulsions that are used, e.g., in polymerization [3].

This review article is focused on ILBSs and GILBSs. Using literature data of (mostly) the last 10 years, we highlight the relationship between surfactant molecular structure and solution properties that are relevant to applications. Of these properties, we dwell on the adsorption parameters of the surfactants at the water/air interface and the characteristics of the formed aggregates. These data are important per se, and are fundamental for the development of novel applications. For example, ILBSs are employed as templates to fabricate nanoparticles (NPs) of different sizes and morphologies. Microemulsions (μEs) formed by these surfactants, both W/O and O/W, were also employed as templates for (free radical) polymerization, where the monomer acts as the “oil” component. Additionally, electrolytes and drugs, especially those with hydrophobic ions that carry opposite charge to the ILBS head-group, change the morphology of the aggregate, e.g., micelle → vesicle, with potential applications in drug delivery. ILBSs with a functional group in the long-chain (e.g., an ester or amide group) undergo reversible transitions—micelle ⇄ vesicle ⇄ organogel—on changing temperature and surfactant concentration. Vesicles and organogels have potential applications in drug delivery and waste-water decontamination (vide infra). ILBSs that carry a polymerizable group (usually a double bond) are advantageously employed in polymerization in μE media because the polymer core is covered with a surfactant shell, leading to enhanced NP stability. The hydrophilic/hydrophobic character of the NPs can be controlled by ion exchange of the anion of (co-polymerized) surfactant with other anions.

Our original premise was to limit the data discussed to ILBSs that conform to the m.p. criterion, i.e., ≤100 °C. A literature survey, however, showed that m.p.s are not reported for many compounds that are classified (by the authors) as ILBSs. In other words, our criterion for considering compounds such as ILBSs and GILBSs is either the availability of m.p. or classification of the surfactant as such by the authors. We included a few applications that use ILs because some of these are weakly surface-active [4].

The issue of surface-active purity of the surfactants employed should not be overlooked. Demonstrating this purity is important because uncertainty in the value of the critical micelle concentration (cmc) bears on the calculated adsorption and micellar parameters [5,6]; removing surface-active impurities from the surfactant solution is, at best, time-consuming and laborious [7]. Another aspect that should be considered when discussing ILBSs is their stability in aqueous media. In this regard, the purity and hence the data of aqueous solutions of ILBSs with BF_4_^−^ and PF_6_^−^ anions should be regarded with some reserve. The reason is that these ions are hydrolytically unstable in water, even at room temperature; this instability was demonstrated by several techniques [8,9,10,11]. This affects the physicochemical properties of the micellar solutions, e.g., cmc, the average aggregation number (*N*_agg_), and the degree of counter-ion dissociation (α_mic_). This problem was mentioned explicitly by some authors (precaution was taken to suppress its effect) [12] but not others [13], even when the ILBSs were heated with methanol at 85 **°**C for 8 h [14]. A literature survey using the search terms (HF, pH and hydrolysis) for the above-mentioned ILBSs showed that the time elapsed between preparing the ILBS solutions and the measurements/applications was not mentioned [15,16,17,18,19,20,21,22,23,24,25,26]. Therefore, we stress that this instability problem should not be overlooked; its potential effect on micellar parameters and other applications should be assessed. Based on these considerations, we feel justified in our decision to exclude from the parts of surfactant adsorption at the water/air interface and micellization of ILBSs with hydrolytically unstable ions, in particular BF₄**⁻** and PF₆**⁻**.

Regarding the abbreviations/acronyms that we employed, we refer to each of the discrete structural moieties using two letters. For example, Im, Py and Vn refer to imidazole, pyridine and vinyl group, respectively. The alkyl moieties attached to the surfactant head-group are listed as C_1_, C_2_, C_3_, and C_4_ for methyl, ethyl, n-propyl, and n-butyl, respectively. Unless specified otherwise, the alkyl groups are n-alkyl. Usually, one of the two groups attached to the heteroatom is a long-chain. Therefore, C_16_C_1_ImBr, C_12_C_1_ImC_8_SO_3_ and C_12_C_1_ImDBS refer to 1-(1-hexadecyl)-3-methylimidazolium bromide, 1-(1-dodecyl)-3-methylimidazolium 1-octanesulfonate and 1-(1-dodecyl)-3-methylimidazolium dodecylbenzene sulphonate, respectively.

The presence of certain functional groups (e.g., amide and ester) in the hydrophobic tail is interesting because it may lead to reversible morphology transitions, e.g., micelle ⇄ vesicles ⇄ ionogel as a function of concentration of the ILBS and solution temperature, due to changes in the hydration of the functional group. Formation of ionogels can be exploited, e.g., in waste-water decontamination and drug delivery [27,28]. Equally important, however, is that the presence of these hydrolysable functional groups contributes to their aerobic biodegradation [29], an issue that is becoming important due to their increased applications, e.g., in high-temperature lubricants, for gas chromatography (stationary phases), in conductive polymer supercapacitors, and as gel polymer electrolyte for sodium-ion batteries [30,31,32,33,34,35].

The environmental impact (biodegradation and toxicity) of ILs and ILBSs should be assessed. Thus, several studies on the relationship between their molecular structures and toxicity showed that the most toxic (to aquatic life) are those carrying aromatic/heterocyclic cations and long alkyl chains; most anions play a minor role in toxicity. Therefore, the synthesis of a new generation of easily biodegradable ILs and ILBSs from renewable sources was studied [36,37,38]. It was shown that ester functionality enhances biodegradation of ILs; furthermore, adding a methyl group to the 2-position of the imidazolium cation and use of alkyl sulfate as a counter-ion also improves the biodegradability [36].

**Figure 2:**
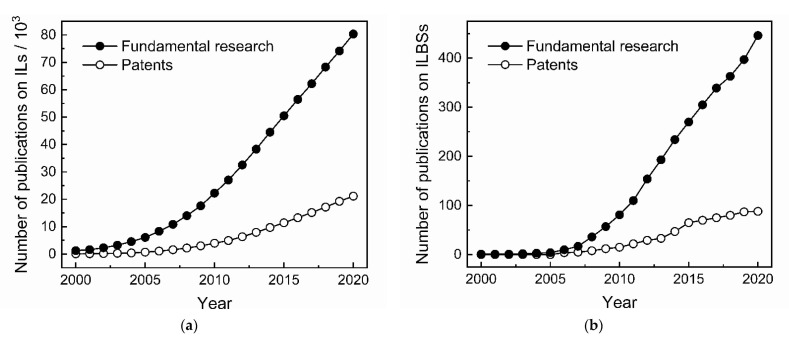
Number of publications on ionic liquids (**a**) and ionic liquid-based surfactants (**b**) between years 2000–2020, source SciFinder database.

The importance of ILs and ILBSs can be readily assessed by examining Figure 2, which shows the number of publications on both classes of compounds from 2000 to 2020 based on a SciFinder database search. Figure 2 clearly shows an exponential growth of these numbers, a consequence of their molecular structure versatility, and hence potential applications in several fields.

## 2. Strategies for Synthesizing Mono-Cationic and Gemini Ionic Liquid-Based Surfactants

The synthesis of ILBS is usually carried out by two consecutive steps: quaternization of amines or phosphines, usually by the S_N_2 mechanism (e.g., by the Menshutkin reaction) using alkyl halides, or alkyl sulphates, followed by anion exchange, where necessary, to yield the desired product. These quaternization reactions are simple and relatively efficient. The amine (or phosphine) is mixed with the desired alkyl halide, followed by stirring and heating. The effects of reaction variables on the yield are those known for S_N_2 reactions. For example, for alkyl halides, the expected order is RI > RBr > RCl (R = n-alkyl group); an increase in the chain length of R decreases the reaction rate [39,40].

The most frequently employed procedures for the Menshutkin reaction include reflux in an appropriate molecular solvent, e.g., acetonitrile. The reaction between 1-methylimidazole and 1-chloroalkanes in acetonitrile under reflux generally requires 2 to 3 days [41,42,43,44,45,46,47]. ILBSs were alternatively synthesized in the absence of solvents, using microwave irradiation [48] or a combination of microwave and ultrasound irradiation [49]. The obtained products are termed first-generation ILs and ILBSs.

**Scheme 1:**
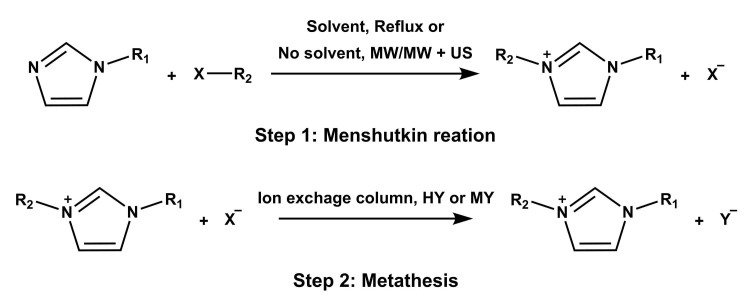
Schematic representation of the synthesis of ionic liquids and ionic liquid-based surfactants. MW and US refer to microwave and ultrasound irradiation, respectively.

Second-generation ILs and ILBSs are obtained from their first-generation counterparts by a metathesis reaction, leading to ILBSs containing bulkier anions, e.g., BF_4_⁻, PF_6_⁻, C_6_H_5_CO_2_⁻ and (CF_3_SO_2_)_2_N^−^. The synthesis steps are summarized in Scheme 1.

**Scheme 2:**
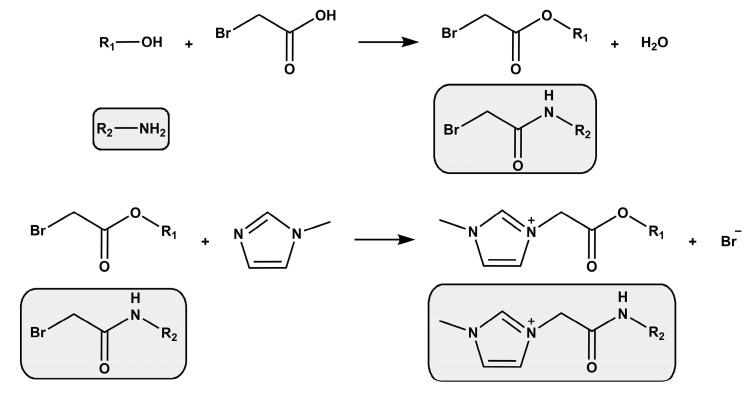
Synthetic route to ionic liquid-based surfactants with ester- or amide group side chain. Redrawn from Kanjilal [46].

The most common ILBSs are synthesized from 1-methylimidazole, which is commercially available at a low cost, along with a small number of other *N*-alkyl substituted imidazoles [40]. ILBSs that carry ester or ether groups are of interest especially because of their biodegradability [50]. Ester- and amide-containing ILBSs were synthesized by the S_N_ reaction of substituted imidazole with α-bromo ester or α-bromo amide. Examples are shown in Scheme 2. ILBS derived from other heterocycles, e.g., pyridine, pyrrolidine, and morpholine, were synthesized by the same general procedure [51,52,53].

**Scheme 3:**
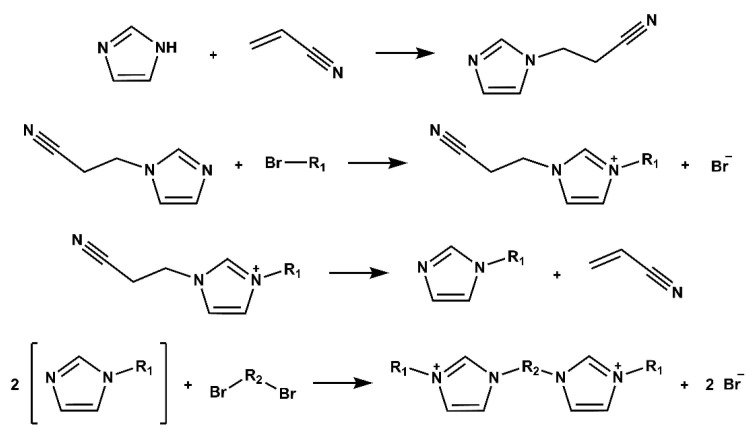
Synthetic route to gemini ionic liquid-based surfactants via the use of a protecting group. Redrawn from Baltazar [45].

GILBSs can be synthesized by two successive S_N_ reactions on imidazole. This requires protecting one of the nitrogen atoms, e.g., by reaction with acrylonitrile if the two attached alkyl groups are different. After the first alkylation, the protecting group is removed by E1cB-type elimination, followed by the second alkylation with a dihaloalkane [43,45], as shown in Scheme 3.

**Scheme 4:**
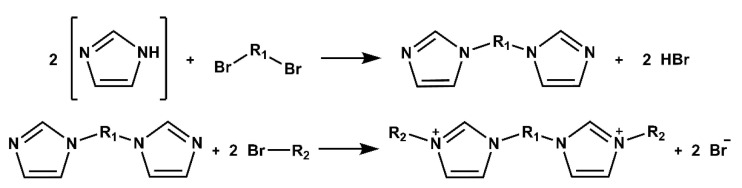
Synthetic route to gemini ionic liquid-based surfactants, via the use of dihaloalkanes. Redrawn from Baltazar [45].

Alternatively, GILBSs were synthesized by reacting imidazole with a dihaloalkane, followed by alkylation of the two “outer” nitrogen atoms, see Scheme 4.

Functionalized GILBSs were synthesized by reacting 1-methylimidazole with diesters containing two bromo substituents and two long chains [54]. Similarly, thioether containing GILBSs were prepared from alkane-1,2-dithiol, alkenes and *N*-bromosuccinimide, the intermediate was then reacted with 1-methylimidazole to form the GILBS [55]. Non-imidazolium GILBSs were prepared by a straightforward one-step reaction, e.g., of tridodecylamine and dibromoalkanes. The class of gemini pyrrolidine-based ILs was synthesized by the consecutive reaction of the secondary amine with a long chain alkyl bromide, followed by reaction of the *N*-alkylpyrrolidine with 1,4-dibromobutane [56].

## 3. Relevant Properties of Aqueous Solutions of ILBS

### 3.1. Compilation and Discussion of the Properties of Aqueous Solution of ILBSs

As already mentioned, one of the most relevant aspects of ILs is their molecular structural versatility, as can be shown, e.g., by imidazolium-based surfactants. In addition to different anions (halides, alkyl sulphate, carboxylates, etc.), different substituents can be introduced at the two nitrogen atoms and at the three carbon atoms of the diazole ring.

Table 1 displays the adsorption parameters of ILBSs in aqueous solutions, in the absence of electrolytes, at 25 °C, whereas Table S1 (in Supplementary Material) shows the micellization parameters of these surfactants. For ease of reading, we maintained the numbering of compounds the same in both Tables. For example, C8C_1_ImCl is compound number 1 in Table 1 and Table S1 in both tables. A similar approach was also applied in Table 2 (GILBSs) and Table S2 (in Supplementary Material).

The data reported cover the period between 2010 and 2020, unless the information is only available before 2010. Only ILs with alkyl chain ≥C_8_ carbons are included, because these surfactants present spherical aggregates at surfactant concentration ≥cmc [57]. The ILBSs are listed by the charge of the group with the longest hydrophobic chain, namely cationic and anionic. ILBSs are listed as biamphiphilic when the alkyl chains of the anion and cation are longer than n-octyl. In Table 1 and Table S1, entries 1–125 refer to cationic, entries 126–152 refer to anionic, and entries 153–163 refer to biamphiphilic ILBSs.

**Scheme 5:**
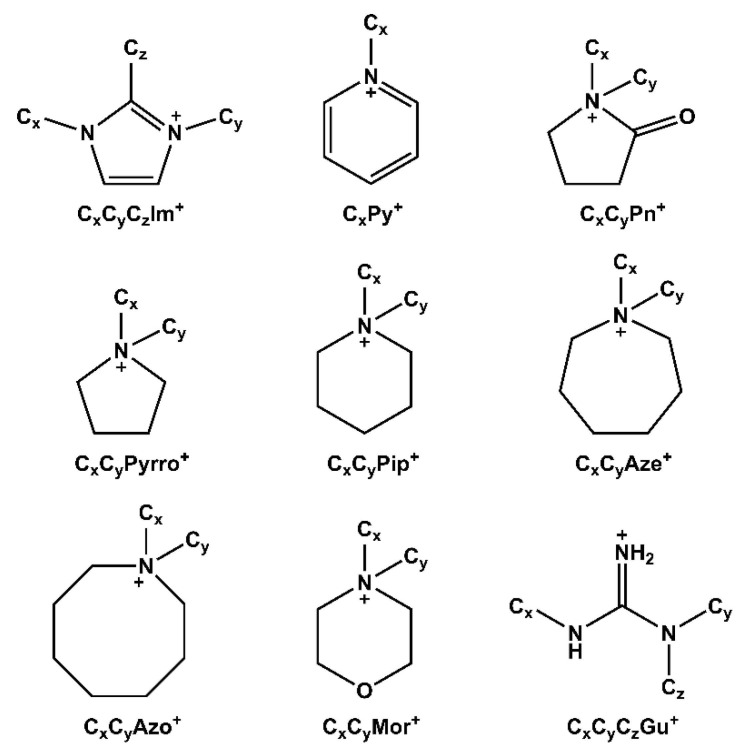
Molecular structures and acronyms of the cationic head-groups of ionic liquid-based surfactants.

Cationic ILBSs are divided according to the structure of the head group (HG), including Imidazolium (Im⁺), pyridinium (Py⁺), 2-pyrrolidinonium (Pn⁺), pyrrolidinium (Pyrro⁺), piperidinium (Pip⁺), azepanium (Aze⁺), azocanium (Azo⁺), morpholinium (Mor⁺), guanidinium (Gu⁺), ammonium (N⁺) and phosphonium (P⁺). Cationic ILBSs carrying the same HG were ordered by the number of carbons of the side chain(s). When two alkyl chains are attached to the heteroatom, e.g., the halides of 1-C_x_-3-C_y_-imidazolium, the classification is based on the length of C_y_. Accordingly, all ILBSs with C_y_ = methyl are listed before those carrying C_y_ = ethyl, independent of the length of C_x_. Additionally, saturated alkyl groups take precedence over unsaturated ones, e.g., ethyl comes before the vinyl group (both with two carbon atoms). In a few cases, the heterocyclic ring carries substituents attached to the ring carbon atoms, C_z_, e.g., when the surfactant precursor is 1,2-dimethylimidazole. In this case, we still list the surfactant according to the length of C_x_ and C_y_, giving priority to the surfactant without C_z_, e.g., C_10_C_1_C_1_Im⁺ comes after C_10_C_1_Im⁺. The molecular structures and acronyms for the ILBSs’ cationic groups are depicted in Scheme 5.

Knowledge of the adsorption and aggregation behavior of ILBSs is required to develop and improve their applications. In face of some relatively large differences between the data reported for the same ILBS, we took a conservative approach by comparing data obtained by the same technique, e.g., surface tension for surfactant adsorption at the water/air interface, and (mostly) conductivity measurements for micelle formation.

Regarding the adsorption parameters, we note that these have greater variation in relation to those obtained by conductivity. This is due to the fact that surface tension measurements are more sensitive to some experimental factors, such as time to reach the surfactant equilibrium at the water/air interface, and the presence of surface-active impurities. This problem can be minimized by analyzing data from articles separately. The data show that as the surfactant hydrophobic chain-length increases, the effectiveness of surface adsorption, given by surface tension at the cmc (γ_cmc_), varies slightly, whereas the efficiency of surface adsorption (p*C*_20_) increases significantly. As expected, *A*_min_ decreases as the size of the hydrophobic chain increases, due to the concomitant closer packing of monomers at the interface. The transfer of the surfactant monomer from bulk aqueous solution to the interface is favored by the increase of the hydrophobic chain length, which explains the increase in the values of the corresponding ∣Δ*G*^0^_ads_∣.

**Figure 3:**
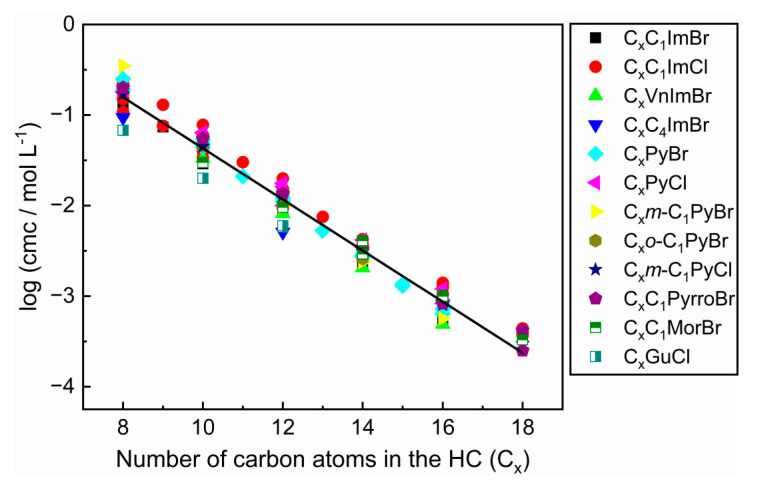
Dependence of log cmc on the number of carbons in the hydrophobic chain (C_x_) for cationic ionic liquid-based surfactants. Data taken from conductivity measurements. The abbreviations of the surfactant head-ions are those listed in the footnotes of Table 1.

Regarding micelle formation, we comment on the values of cmc of Table 1 and Table S1 because this is the main parameter employed to calculate several adsorption and micellization properties. Figure 3 shows the dependence of log cmc on the number of carbon atoms (C_x_) of the hydrophobic chain (HC). The observed linear relationship is expected because the value of the free energy of transfer of a CH_2_ group from bulk aqueous pseudo-phase to the interior of the micellar aggregate (Δ*G*^0^**_CH_**_2_) should be independent of the nature of HG. Consequently, the slope is expected to be independent of the charge and nature of the surfactant head-ions.

Figure 3 is an example of the Stauff–Klevens rule,

log cmc = A − B x,(1)

where A is a constant that depends on the experimental conditions, the structure of the surfactant monomer and counterion, and B refers to the effect of each additional CH_2_ (in x) on cmc. Application of Equation (1) to the results of Figure 3 yields Equation (2) for cationic ILBSs:

log cmc = 1.46 ± 0.04 − 0.282 ± 0.003 × R^2^ = 0.975 (2)

Application of Equation (1) to the data of Table S1, for anionic and biamphiphilic ILBSs, yield Equations (3) and (4), respectively:

log cmc = 1.12 ± 0.17 − 0.327 ± 0.017 × R^2^ = 0.954 (3)

log cmc = −0.4 ± 0.3 − 0.30 ± 0.03 × R^2^ = 0.966 (4)

The most relevant point is that the values of the slopes are of the same order, in agreement with the above-mentioned independence of Δ*G*^0^**_CH_**_2_ of the nature of the head-ions.

Other aggregation parameters, such as Gibbs free energy of micellization (Δ*G*^0^_mic_), enthalpy of micellization (Δ*H*^0^_mic_), degree of counterion dissociation of the micellar aggregate (*α*_mic_) and average micellar aggregation number (*N*_agg_), are also important. Unlike the values of cmc, which are calculated directly and precisely from solution conductivity, the above-mentioned parameters are published less frequently, and their calculation is subject to uncertainties. We dwell on this point because of its relevance to the calculated aggregation parameters that are employed in the correlation between surfactant molecular structure and solution properties. The usual procedure is to calculate the value of Δ*G*^0^**_mic_** is Equation (5), where cmc is given on the mole fraction scale, χ_cmc_ [146]:

Δ*G*^0^_mic_ = (2 − *α*_mic_) *RT* ln χ_cmc_ (5)

The value of Δ*H*^0^_mic_ is then calculated from the dependence of cmc on the temperature; the value of Δ*S*^0^_mic_ is calculated from Gibbs free energy relationship:

Δ*G*^0^**_mic_** = Δ*H*^0^_mic_ − *T*Δ*S*^0^_mic_ (6)

As argued elsewhere, this sequence of calculations can be problematic because the value of *α***_mic_** is calculated using Frahm’s (simple) equation. The latter disregards the contribution of the micelle (a macroion) to solution conductivity. Evans’ equation takes into consideration the micelle contribution to solution conductivity above the cmc [147]. The result is that α_mic_ (Frahm) > *α*_mic_ (Evans); error in *α*_mic_ is reflected in the calculated values of Δ*G*^0^_mic_, and Δ*S*^0^_mic_; see Equations (5) and (6). Note that Evans’ equation requires knowledge of *N*_agg_, whose value can be calculated, e.g., from static light scattering data, or from the volume of the surfactant monomer. As has been shown, a relatively large uncertainty in *N*_agg_ leads to a negligible effect on the value of *α*_mic_ [148]; i.e., the use of Evans’ equation is recommended.

On the other hand, the values of Δ*H*^0^_mic_ that are calculated indirectly by the van Hoff treatment and directly by ITC usually do not agree. The reason is that there is no provision in the former treatment for the effects of increasing temperature on micellar parameters, e.g., α_mic_, *N*_agg_ and monomer dehydration [149]. Effects of these variations are “embedded” in the value of Δ*H*^0^_mic_ calculated by ITC. As shown by Equation (6), the above-mentioned uncertainties in Δ*H*⁰_mic_ are carried over to Δ*S*^0^_mic_.

Analysis of the available data from ILBS shows that Δ*G*^0^_mic_ decreases as a function of increasing the number of CH_2_ groups in the HC; i.e., the micellization becomes more favorable. Regarding α_mic_ and *N*_agg_, it is seen that the former decreases and the latter increases as a function of increasing the length of the hydrophobic chain; these effects are consequences of the smaller surface area of surfactants with longer HC.

**Figure 4:**
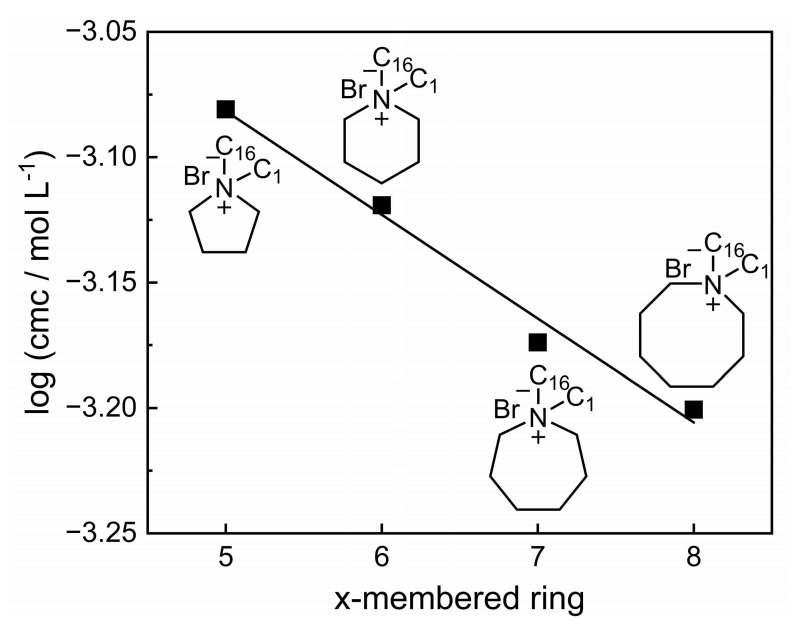
Log critical micelle concentration (cmc) as a function of the size of heterocyclic amine ring structures of cationic ionic liquid-based surfactants. Data taken from Schnee and Palmer [150].

Besides the length of the hydrophobic chain, the effects of some other structural variables in the HG were also probed. Schnee and Palmer [150] studied the effect of the size of heterocyclic ring structures (5- to 8-membered rings: C_16_C_1_PyrroBr, C_16_C_1_PipBr, C_16_C_1_AzeBr and C_16_C_1_AzoBr, respectively) on their aggregation properties. Increasing the size of HG led to a decrease in the value of cmc, from 0.83 to 0.67 mmol L⁻^1^; see Figure 4. The reason is that increasing the size and hydrophobicity of the HG results in energetically unfavorable surfactant–solvent interactions in the bulk aqueous pseudo-phase, as well as stronger interactions at the micelle surface, resulting in lower cmc values.

**Figure 5:**
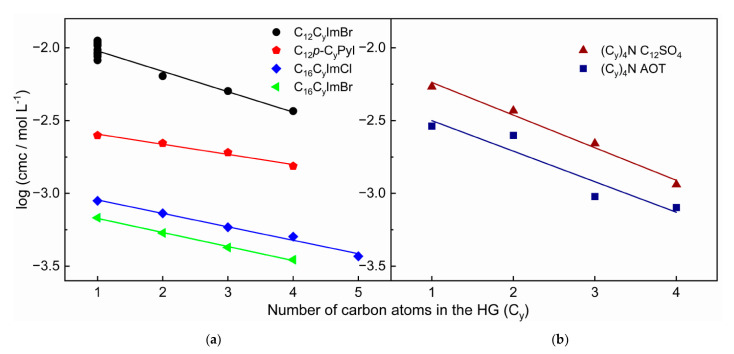
Dependence of log cmc on the number of carbons in the head group side chain (C_y_) of (**a**) cationic ionic liquid-based surfactants and (**b**) anionic ionic liquid-based surfactants. Data taken from conductivity measurements; see references [80,91,126,151,152].

Keppeler et al. [91] studied the effect of the length of alkyl side chain (C_y_) of imidazolium-based surfactants (C_16_C_y_ImBr and C_16_C_y_ImCl) on their adsorption and aggregation properties. It was found that increasing the length of C_y_ from methyl to n-pentyl led to a linear decrease in the values of log cmc; see Figure 5. The slope of log cmc versus C_y_ (change in the HG) is about 3 times smaller than the corresponding slope for introducing methylene groups in the HC (hydrophobic chain). This behavior is expected, because on micellization, there is more dehydration of most of the CH_2_ groups in HC (whose micelle interior is oil-like) than any CH_2_ in the head group. Increasing the length of the alkyl side chain results in an increase in Amin, probably due to steric repulsion between the increasingly voluminous C_y_ chains. A corollary to this statement is that the increase in the length of C_y_ leads to less surfactant molecules at the water/air interface, in agreement with the decrease in Γmax and increase in γ cmc. The adsorption of a more hydrophobic HG is favored, as shown by the increase in |ΔG^0^ads| and pC_20_.

Another structural variable that can be analyzed is the effect of the position of methyl groups in heterocyclic ring on the value of cmc. Sastry et al. [99] studied the aggregation behavior of 1-octylpyridinium and 1-octyl-2-, -3- or -4-methylpyridinium chlorides. The data show that the ILs with methyl-substituted pyridinium cations have lower cmc values than the parent pyridinium cation, indicating that the presence of methyl group in pyridine ring increases its hydrophobicity, in agreement with published values of log P (the partition coefficient of a substrate between mutually saturate water and n-octanol), 0.73 and 1.2 for pyridine and 4-methylpyridine. The position of the methyl group in the 1-ocylypyridinium ring has a small effect (6%) on the value of cmc.

The effect of the counterion was also analyzed. Since this topic was not investigated in detail, we limit our analysis to ILBSs with halide anions. Kim and Ao [75] studied the properties of aqueous solutions of ILBSs with different halide anions (C_12_C_1_ImCl, C_12_C_1_ImBr and C_12_C_1_ImCl). The order of cmc and αmic at 25 °C was (cmc in mmol kg⁻^1^; αmic calculated by Frahm’s equation): C_12_C_1_ImCl(15.1; 0.44) > C_12_C_1_ImBr(10.6; 0.25) > C_12_C_1_ImCl (5.2, 0.15). Counterions are adsorbed at the micellar interface primarily by strong electrostatic interactions. For halide anions, this adsorption depends on the balance between anion polarizability [153] and radius of the hydrated anions [154]. As a consequence of the increase in the size of the hydrated anions, the chloride counterions are located further away from the micellar interface than the hydrated iodide counter ion [155]. That is, the micellar surface potential decreases in the order ILBS-Cl > ILBS-Br > ILBS-I, in agreement with the above-mentioned values of cmc and *α*mic.

### 3.2. Compilation and Brief Discussion of the Properties of Aqueous Solution of GILBSs

**Scheme 6:**
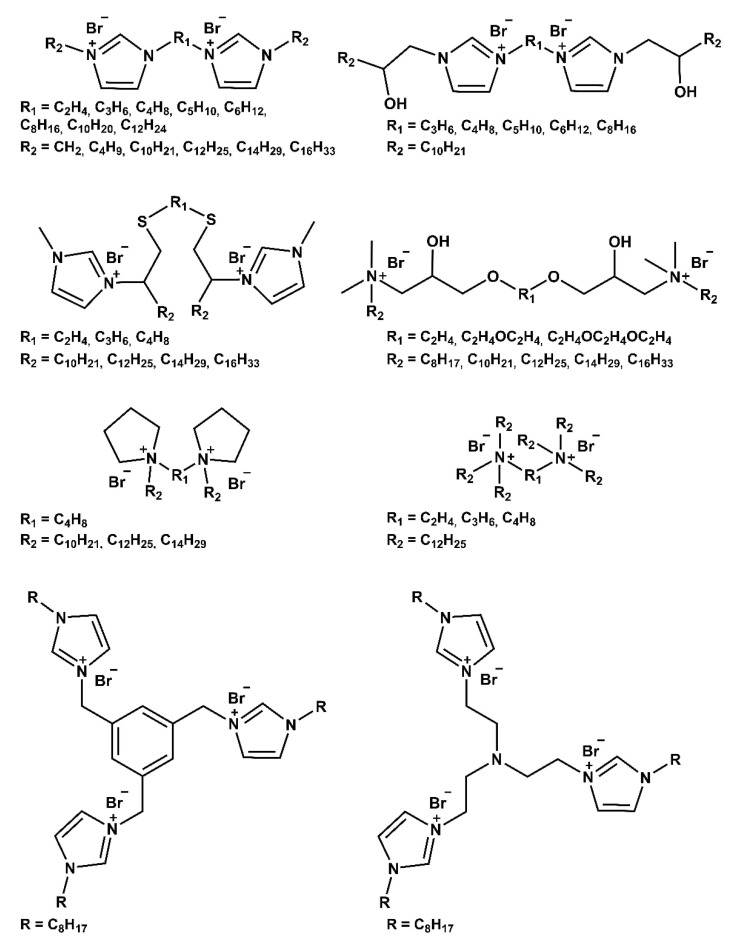
Molecular structures of the gemini ionic liquid-based surfactants reported in this review.

The GILBSs in Table 2 and Table S2 are listed in a similar way to the ILBSs. They are first divided according to the structure of cationic headgroup: imidazolium (Im), thioether-functionalized methylimidazolium (SMeIm), hydroxyl-functionalized imidazolium (OHIm), quaternary ammonium (C_x_C_y_C_z_N) and pyrrolidinium (Pyrro). Within each category, they are ordered by the number of carbon atoms of the “spacer” and then by the number of carbon atoms of the hydrophobic chain(s). Accordingly, imidazolium-based GILBSs with a spacer containing two carbon atoms are presented before those with a spacer containing three carbon atoms. At the bottom of Table 2, we present two examples of ILBSs containing three long chains. To the best of our knowledge, there are no reports on GILBSs containing unsaturated alkyl groups. The molecular structures for the GILBSs cationic groups are depicted in Scheme 6.

The first members reported in the literature that conform to the m.p. criterion (≤100 °C) are quaternary ammonium surfactants that carry the hydroxyl group; the latter was considered important to promote intermolecular hydrogen bonding that lowers the melting point [161]. As compared to their single-chain counterparts, the GILBSs have an increased propensity to form aggregates and efficiently reduce surface tension. The same trend is observed for conventional single chain and gemini surfactants [1,164].

**Figure 6:**
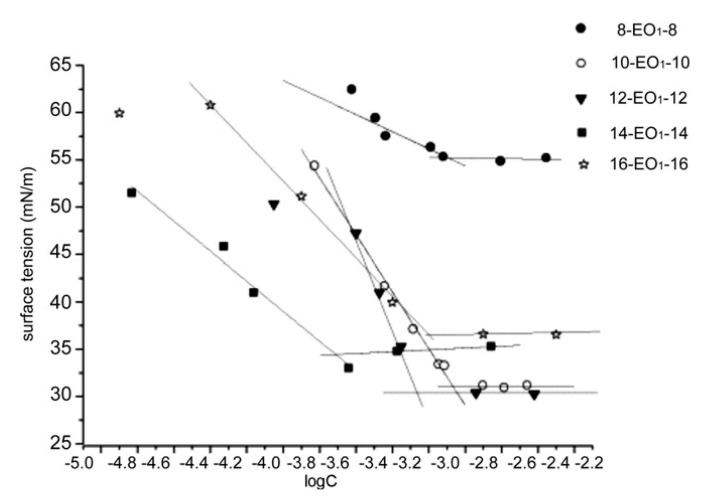
Dependence of cmc on the number of carbon atoms in the hydrocarbon chain (from C_8_ to C_16_) for the surfactant series with one ethylene oxide spacer [161]. Reprinted with permission from ref. [161]. Copright 2021 Elsevier.

As can be seen from Table 2, the general trends for ILBSs are also observed for GILBSs. For example, for the same spacer, the cmc values are expected to be lower with increasing the length of the hydrophobic chain(s). One example is the quaternary ammonium surfactants with HC from 8 to 16 carbon atoms and 1–3 ethylene oxide units (EOs) as spacers [161]. Figure 6 shows the dependence of cmc on the number of carbon atoms in HC for one series. They are not in the expected order (e.g., cmc for C_16_ > cmc for C_14_), probably due to the possibility of self-coiling or formation of pre-micellar aggregates [161].


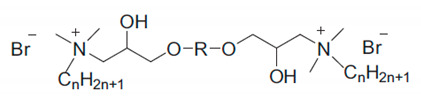


**Figure 7:**
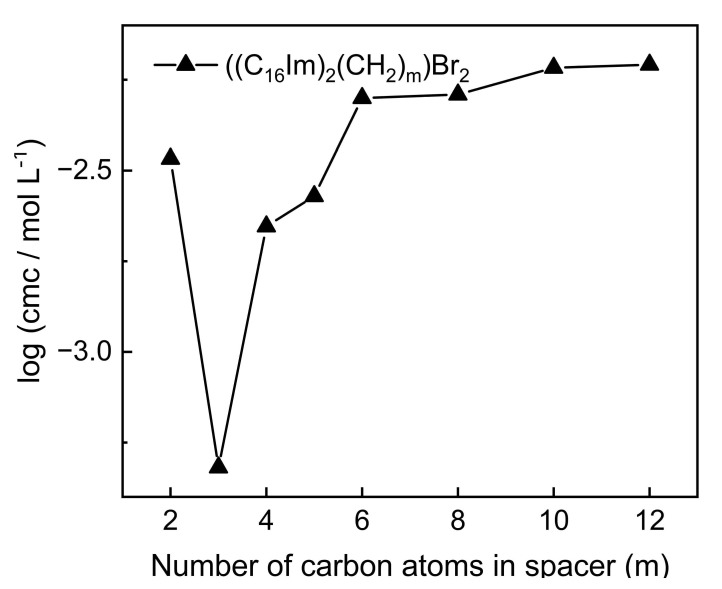
cmc as a function of spacer length from m = 2–12 in ((C₁₆Im)₂(CH₂)_m_)Br₂ series [157].

The effect of the spacer length on cmc is complex because it is a sum of several factors, including rigidity of the molecule, hydrogen bonding (where applicable), hydration of HG, Coulombic repulsion between HGs. This complex behavior was shown by Pal et al. [157] for a series of GILBSs containing two imidazolium rings in the HG and spacer from 2 to 12 methylene groups. They observed a lower cmc value for the (CH_2_)_3_ spacer, after which the cmc values increased and then reached a plateau (Figure 7). This was explained in terms of rigidity and planar nature of the imidazolium HG, which interfere with the spacer packing, leading to independent behavior of each single chain beyond a spacer of (CH_2_)_3_.

**Figure 8:**
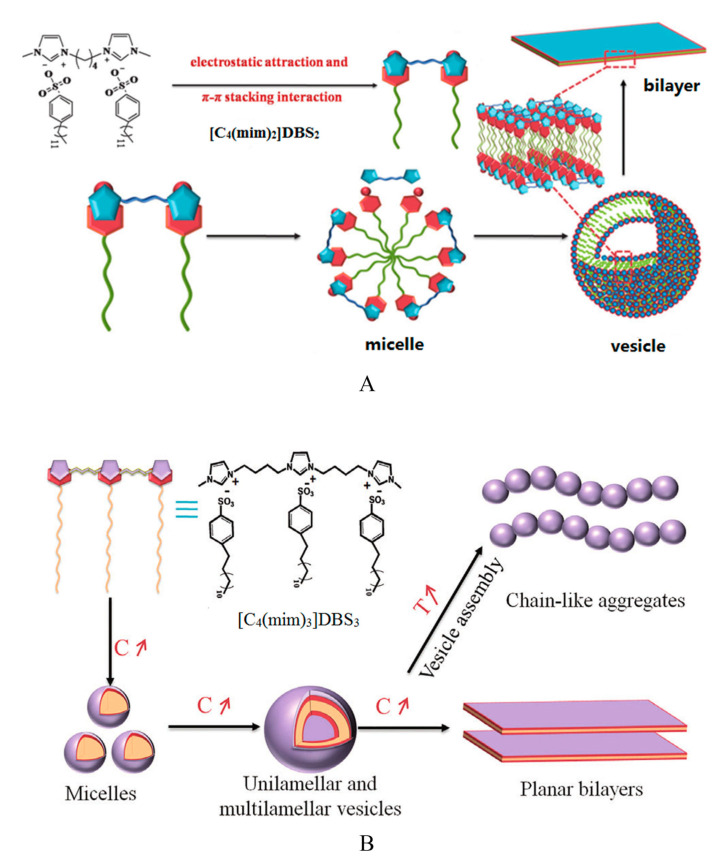
Dependence of the aggregate morphologies on the structure of the cation and anion in gemini (**A**) and trimeric (**B**) biamphiphilic surfactants.

The possibility of “tuning” the morphology of the colloidal aggregate by adjusting the length of the two component ions of biamphiphilic compounds is nicely shown in Figure 8 for dimeric and trimeric surfactants with ring-containing cation and anion. The multitude of possibilities is relevant to applications of these surfactants that may require, e.g., a vesicle, a bilayer, or a wormlike aggregate. These aggregates can be obtained by a judicious choice of (in Figure 8) the length of the spacer in the cation and the HC of the anion [1].

## 4. Applications of IL-based Surfactants

### 4.1. Nanotechnology

ILBSs have been explored in various fields, including chemical synthesis and catalysis [165,166,167,168,169,170], drug delivery [171,172,173,174,175,176,177], biomass conversion [178,179,180,181,182,183], liquid crystal development [184,185,186], decontamination [187,188,189,190,191,192,193] and formation and stabilization of metal NPs [61,194,194,195,196,197,198,199,200,201,202,203,204,205,206,207,208,209,210,211,212,213,214,215,216]. We dwell here on the synthesis and stabilization of mesoporous nanoparticles (MNPs), including mesoporous silica (MSNPs).

MNPs have small sizes and large surface areas that make them important materials in various fields, e.g., medicine, electronics, electrical and magnetic materials, catalysis and fabrication of novel chemical and biological sensors. Apart from synthesis, controlling the average size, surface area, porosity and stability of MNPs is a challenging task and, if achieved, contributes to their wider applications. The synthesis of uniform-sized MNPs with controlled morphology is feasible, thanks to the relative ease of tailoring the properties of ILBSs to play a required role. Consequently, various morphological architectures, e.g., micelles and vesicles, were employed as soft templates for the formation and stabilization of these MNPs. The latter particles are, however, only kinetically stable and will aggregate to thermodynamically more stable larger particles due to Ostwald ripening. This spontaneous process occurs because larger particles are energetically favored, due to their lower surface-to-volume ratio. Therefore, stabilization of the formed NPs is essential for any application. The effect of the colloidal template depends, inter alia, on the length of its HC, the nature of the HG and the counter ion. The reason is that these structural factors determine the value of cmc and the morphology of the colloidal species (spherical micelle, vesicle, etc.).

This dependence was nicely shown by studying the effect of the length of the hydrophobic group on the average particle size and stability of Ag NPs, by a series of 1-R-3-MeImX (R = C_8_, C_10_, C_12_; X = Cl⁻, Br⁻), C_12_Me_3_NBr and sodium dodecyl sulfate (SDS). It was found that the length of the surfactant HC is determinant to the stability of the NPs. For example, (cationic) micelles of the surfactant with R = C_8_ did not provide enough stabilization, so the synthesized Ag NPs coalesced immediately. Increasing the length of R from C_8_ to C_12_ led to an increase in stability and concentration of the formed Ag NPs due essentially to hydrophobic interactions between surfactants and surface of the Ag NPs [217].

**Figure 9:**
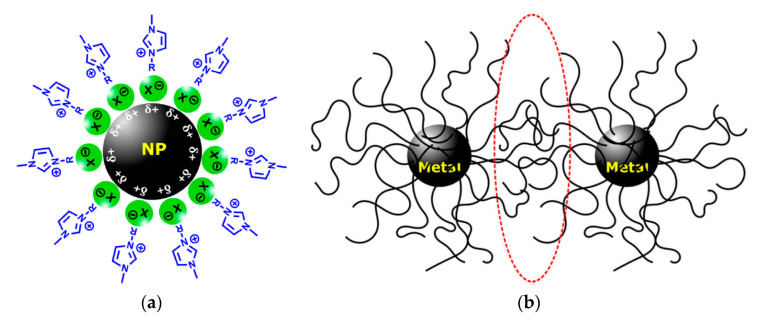
Schematic representation of the electrostatic (**a**) and steric (**b**) stabilization mechanisms of nanoparticles. In the former mechanism, the nanoparticles (NPs) are stabilized due to electrostatic repulsion of the positively charged outer layer. Steric repulsion between the surfactant hydrophobic chains contributes to NP stabilization [219]. Reprinted with permission from ref. [219]. Copright 2021 Springer Nature.

As shown by Figure 9, stabilization of the NPs by ILBS is achieved mainly through (i) electrostatic interactions of their ions with the NP and (ii) steric repulsion between the sheaths covering the generated NPs. Both mechanisms create a protective coating around the NPs, thereby hindering their aggregation and control the distribution of their sizes. Judicious selection of the molecular structures of ILBSs is required for fabricating NPs with controlled sizes, shapes and porosity that can be useful in various biological applications and catalysis [218,219,220].

**Figure 10:**
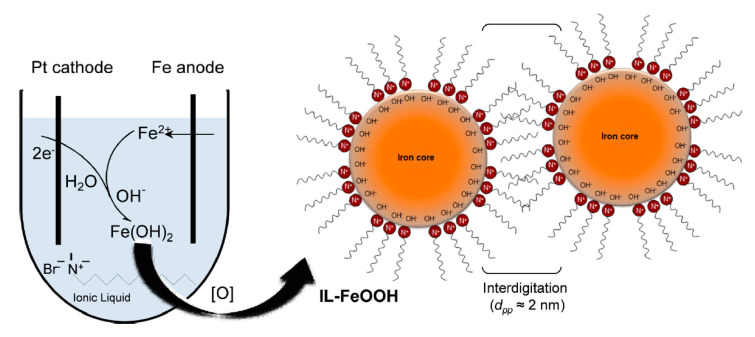
Schematic representation for the production of FeO_2_H particles coated with an ionic liquid-based surfactant layer [194]. Reprinted with permission from ref. [194]. Copright 2021 Elsevier.

Aqueous micellar solutions of dodecyltrimethylammonium bromide were found to stabilize α-FeO_2_H NPs and decrease their average size, when compared with those prepared in absence of the surfactant. An electrochemical method was used to synthesize nano-sized α-FeO_2_H particles (average diameter = 5–10 nm) in the presence of the surfactant. The proposed reaction pathway for the electrosynthesis of ILBS-FeO_2_H NPs is shown in Figure 10, where FeO_2_H NPs were formed inside inverse micelles [194].

Because of their surface and optoelectronic properties, nano-sized α-FeO_2_H particles with an average diameter of 1–100 nm are used as a semiconductor catalyst in the degradation of chlorinated compounds [99,100], e.g., 2-chlorophenol (2-CP). Note that α-FeO_2_H is practically inactive in the absence of oxidizing agents (e.g., H_2_O_2_) that provide the hydroxyl radicals (·OH) necessary for 2-CP oxidative-degradation. IL-FeO_2_H degraded 56% and 85% of 2-CP when the catalyst was irradiated with visible light (350–600 nm), in the absence and presence of H_2_O_2_, respectively. It is presumed that the surfactant head-groups in the IL-FeO_2_H reverse micelle trapped the photogenerated electrons at the conduction band and simultaneously decreased the recombination rate of photo-induced electron-hole pairs at the valence band; resulting in the enhancement of the 2-CP degradation.

A similar magnetite (Fe_3_O_4_) NP stabilization mechanism by the reverse micelles of the GILBS (16-2-16), α,ω-bis(3-decylimidazolium-1-yl) ethane dibromide, and other gemini cationic surfactants were suggested. The Fe_3_O_4_ NPs were synthesized by a hydrothermal treatment of an equimolar mixture of FeCl_3_ plus FeSO_3_ in the presence of GILBS (150 °C, 24 h), followed by removal of the excess surfactant by extraction with hexane. The positively charged surface-active Fe_3_O_4_ NPs thus obtained were used to extract Au and Ag NPs from their aqueous solutions. The Au and Ag NPs were solubilized in water by single-chain surfactants (cetyltrimethylammonium bromide, CTABr or SDS); hence, they have positive and negative charges, respectively. Consequently, the extraction was favored by NP–NP interactions, including electrostatic, in the case of SDS-stabilized Au- and Ag NPs, and hydrophobic, for CTABr and SDS stabilized metal NPs [221].

**Figure 11:**
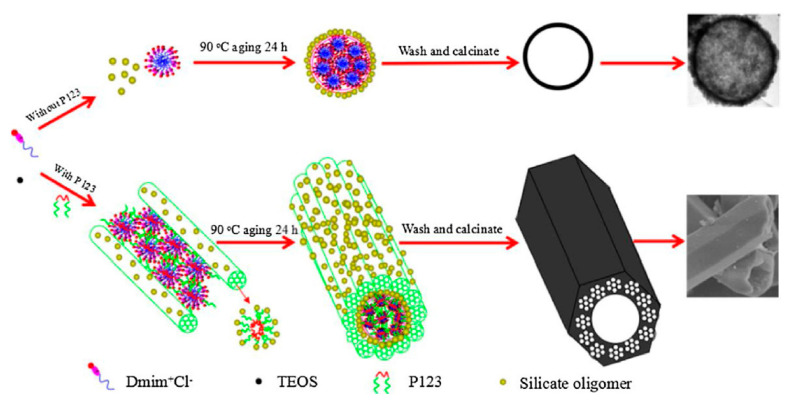
Schematic representation of the effects of C₁₀C₁ImCl and ionic liquid-based surfactant plus the co-template (P123) on the morphology of the formed SiO_2_ NPs. HCl is used to hydrolyze tetraethyl orthosilicate silane (TEOS), the silicate precursor [195]. Reprinted with permission from ref. [195]. Copright 2021 Elsevier.

Han et al. [195] used a novel sol-gel method to synthesize hollow silica spheres and tubes with disordered and ordered mesopores by using C_10_C_1_ImCl and the nonionic surfactant P123 (a copolymer between PEG and PPG, of the average composition PEG_20_PPG_70_PEG_20_) as the template and co-template, respectively. The micelle/P123 co-assembly is supposed to be responsible for the formation of the silica morphology and mesostructure. Two strategies were explored for the fabrication of SiO_2_ hollow spheres, namely using the ILBS alone, and using the ILBS plus P123 as co-template. It was observed that the shape and size of the SiO_2_ nanospheres depend on the IL concentration; i.e., at low dosage (0.0025 mol), flake-like silica was observed which was converted into bulk-like silica when the IL concentration was increased to 0.005 mol. Increasing the IL concentration to 0.015 mol resulted in uniformly sized (average radius = 3.8 nm) hollow SiO_2_ spheres. In the second strategy, it was observed that increasing the molar ratio of P123/C_10_C_1_ImCl from 0.03 to 0.04 resulted in the SiO_2_ morphology changing from spheres with an average diameter of 5 μm to a long curved tube. Upon a further increase of the above-mentioned ratio to 0.07, the silica tubes become longer and prism morphology began to appear. Addition of P123 strengthened the binding between adjacent spheres and allowed them to adhere to each other more tightly. It is interesting to note that using P123 alone resulted in the formation of bulk silica without any tubes, demonstrating the significant impact of both P123 and C₁₀C₁ImCl on the morphology of silica tubes; see Figure 11.

Based on Figure 11, these authors explained the role of the template (C**₁₀**C**₁**ImCl) and the co-template (P123) on the morphology of the fabricated SiO_2_ NPs. When only ILBS was present at a concentration less than its cmc (0.062 mol L⁻^1^), the tetraethyl orthosilicate silane (TEOS) hydrolyzed quickly, while the hydrolysate condensed slowly. As a result, stable small oligomers Si(OC_2_H_5_)_4−x_(OH)_x_ were formed. At [C_10_C_1_ImCl] less than its cmc, the Si(OC_2_H_5_)_4−x_(OH)_x_ dissolved into the spherical micelles present; these coalesced into larger spheres that, upon washing and calcination, produced hollow SiO₂ nanospheres. In the second case, i.e., in the presence of the co-template, the tail of the C_10_C_1_ImCl interacted hydrophobically with the PPO block of the P123. This led to the formation of P123/C_10_C_1_ImCl mixed micelles. At higher concentrations of P123, the PEO blocks of the P123 interacted with the oligomers through hydrogen bonding to form the cylinder-like micelles. Through aging, the small oligomers crystallized on the surface of the long cylindrical micelles that, on washing and calcination, formed the hollow prism-like tubes.

Therefore, the formation of ILBS-additive mixed micelles offers a versatile approach for the fabrication of MNPs of controlled geometry suitable for many applications. In addition to co-polymers (e.g., P123), the additive can be a relatively hydrophobic ion with an opposite charge (to that of the micellar surface), leading to the growth of the spherical micelles and the incorporation of TEOS hydrolysis oligomers therein. This approach was used to prepare MSNPs containing the drug ibuprofen (2-(4-(isobutylphenyl)propanoic acid; pKa = 4.54) by hydrolysis of TEOS in the presence of the ILBS C_8_C_1_ImCl. At the working pH (=7.5) the (relatively hydrophobic), ibuprofen anions were incorporated into the micelles (by ion exchange with Cl⁻), leading to the formation of MSNPs with large surface area (as high as 812 m^2^ g⁻^1^) and pore volumes (1.25 cm^3^ g⁻^1^) with ibuprofen loading of 46 wt.%. The residual ILBS in the ibuprofen-MSNPs was relatively high (13%); its release under drug delivery conditions should be assessed [196].

MSNPs were successfully synthesized by acid hydrolysis of TEOS in the presence of two ILBSs, C₁₆C₁ImBr and C₁₈C₁ImBr. The average particle sizes (nm), surface area (BET; m^2^ g⁻^1^) and pore volume (cm^3^ g⁻^1^) of the formed MSNPs were affected by the alkyl chain length of the ILBS template. Namely, smaller, more porous NPs were obtained using the C₁₈C₁ImBr template. Thus, the ratios of the above-mentioned properties (C₁₈C₁ImBr/C₁₆C₁ImBr) were found to be 0.68, 1.05 and 1.70, respectively [197]. Therefore, changing the length of the hydrophobic tail of the ILBS is another variable that can be exploited for controlling the properties of MNPs.

**Figure 12:**
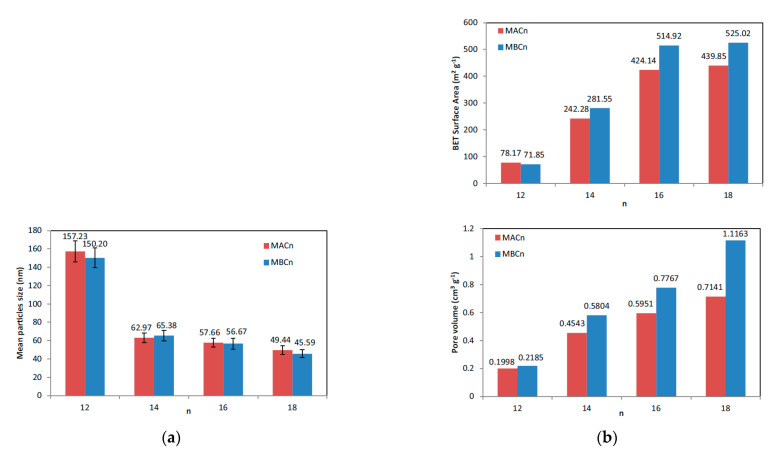
(**a**) MACn = mesoporous silica nanoparticles prepared by strategy (i) and (**b**) MBCn = mesoporous silica nanoparticles prepared by strategy (ii) [198]. Reprinted with permission from ref. [198]. Copright 2021 Elsevier.

Similar results were observed when pyridine-based ILBSs (C₁₂PyBr, C₁₄PyBr, C₁₆PyBr and C₁₈PyBr) were employed as templates for fabricating MSNPs, using triethanolamine (TEA) to get well-dispersed MSNPs. The authors employed two strategies: (i) The template was fed with the premixed and preheated TEA and TEOS. (ii) TEA, template and water were preheated and stirred before the addition of the TEOS. With both strategies, the authors assessed the role of alkyl chain length of pyridinium ILBSs in the formation of MSNPs. It was observed that with both strategies, except for a minor change in the results, the size of the MSNPs decreased with increasing the length of HC, whereas the porosity and surface area of MSNPs increased in the same direction. This is shown by the reported results of C₁₈PyBr/C₁₂PyBr, for the mean particle size (nm), BET surface area (m^2^ g⁻^1^) and pore volume (cm^3^ g⁻^1^), respectively: 0.31, 5.63 and 3.57 [198]. The same authors employed experimental design to optimize the average surface area and particle size of the fabricated MSNPs and studied the loading and release of the drug quercetin (a flavonoid employed to prevent/treat some cancer types). The loaded drug was then checked for its release profile using a dialysis bag technique. It was observed that, due to the drug–MSNP interaction, the crystallinity of quercetin changed to amorphous, which increased its bioavailability. The 32% cumulative release of the drug was obtained in the MSNP-loaded drug against the unloaded drug. These results suggest that with MSNPs, it is possible to have a slower release of drugs [199]. Drug release profile is illustrated in Figure 12 [198].

**Figure 13:**
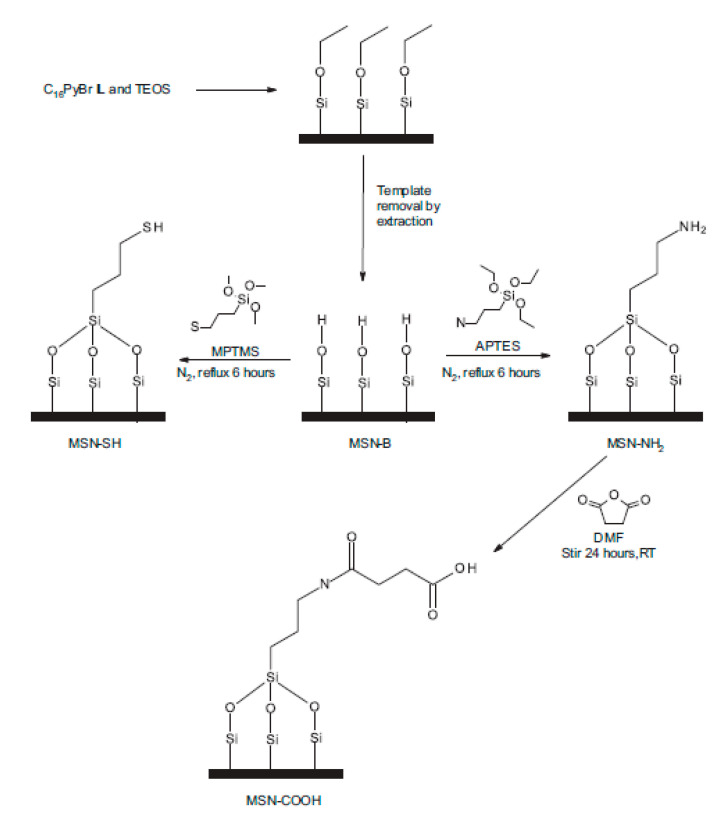
Schematic representation for the fabrication of functionalized mesoporous silica nanoparticles [200]. Reprinted with permission from ref. [200]. Copright 2021 Elsevier.

C_16_PyBr was used as the soft template for the production of uniformly shaped spherical MSNPs with a particle diameter of 35 to 40 nm. These MSNPs were then functionalized to obtain MSNP-NH_2_, MSNP-SH and MSNP-COOH surface-functionalized particles through a post-grafting technique, during which the functional groups were covalently bonded to the silanol group (Si–OH) on the external or internal pore surface, see Figure 13. The functionalized MSNPs thus obtained were used as carriers to load drugs employed for cancer treatment, including hydrophilic gemcitabine, and hydrophobic quercetin [200].

**Figure 14:**
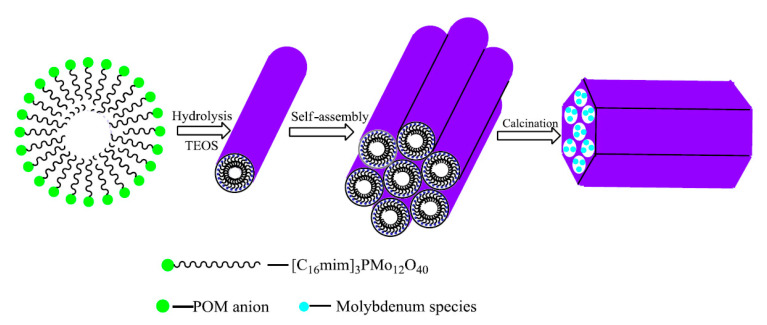
Schematic representation for the fabrication of functional molybdenum-containing mesoporous silica nanoparticles [201]. Reprinted with permission from ref. [201]. Copright 2021 Elsevier.

In their efforts to enhance the desulfurization of diesel oil and gasoline, Zhang et al. [201] explored the oxidative desulfurization of fuels using an ILBS with polyoxometalate as anion. The ILBS (1-hexadecyl-3-methylimidazolium phosphomolybdic compound ([C_16_C_1_Im]_3_PMo_12_O_40_)) was used to fabricate the functional molybdenum-containing ordered mesoporous silica, as shown in Figure 14. Herein, the C_16_ chain acted as the template of ordered mesoporous, whereas the polyoxometalate anion acted as the source of active metal sites. Under optimal conditions, dibenzothiophene was completely removed in 50 min, and the catalyst efficiency was found to be 91% after recycling nine times [201].

C₁₂C₁ImCl based template was used to fabricate zeolite nanocrystals by using a hydrothermal technique. In the absence of the ILBS, unstable nanocrystals in the micrometer range were obtained. In the presence of the ILBS, however, much smaller particles were obtained, with average size and surface area of <30 nm and 443.6 m^2^ g⁻^1^, respectively. The post treatment to remove the ILBS template included direct calcination at 550 °C and extraction with refluxing ethanol for 12 h; the latter treatment resulted in smaller particles with a larger average surface area. For example, the following ratios (ethanol extracted sample/directly calcinated sample) were observed for the micropore volume: 0.90 and 0.94 for hydrothermal heating times of 54 and 102 h, respectively. The ratios for the surface area were 2.19 and 6.82 for hydrothermal heating times of 54 and 102 h, respectively [202].

Nano-particle aggregation/growth during the reaction that causes their catalytic deactivation was hindered through stabilizing the MNPs using ILBSs. In their work on the catalytic hydrochlorination of acetylene, Hu et al. [203] used tetra(n-butyl)phosphonium carboxylates (octanoate, dodecanoate, tetradecanoate, hexadecanoate and octadecanoate). The self-assembling of the ILBSs along with the high reactivity of NPs was used to reduce the deactivation of the metal catalysts through establishing the effective redox cycle between Pd⁰ and Pd^II^. The ILBSs form a protective layer around the NPs, hindering their aggregation. Herein, the authors observed no obvious disparity in dispersion degree or particle size of Pd NPs (narrow size distribution in the range of 2.4–4.4 nm and the mean size ~3.2 nm) when the alkyl chain of the anions of ILBSs was changed from C_7_ to C_17_.

Pt and Au catalysts were fabricated using the respective NPs/ILBSs with the stearate anion simply by blending the surfactant with the precursor PtCl_2_ and HAuCl_4_·4H_2_O at 120 °C, respectively. The authors observed highly ordered lattice fringes in a Pt NP with particle sizes in the range of 1.5 nm, whereas in the case of Au, the particle size was >20 nm. When tested for their catalytic performances, Pd NPs/ILBSs systems with the longer carboxylate showed maximum acetylene conversion into vinyl chloride of 93.04%, whereas the corresponding ILBS with octanoate anion showed 76.23%, suggesting the impact of alkyl chain length of the anion on the catalytic performance. For the Pt and Au NP/ILBSs systems, the catalytic performance of the stearate ILBS was 74.25% and 64.56% respectively. Furthermore, the basicity of the carboxylate anion was effective in absorbing and activating acetylene and HCl. It was observed that 1 mol of ILBS with the stearate anion absorbed approximately 2 mol of HCl and 0.6 moles of C_2_H_2_ at the reaction temperature. This study shows that metal NPs/ILBS systems are promising as substitutes for toxic mercury catalysts in the hydrochlorination of acetylene [203].

**Figure 15:**
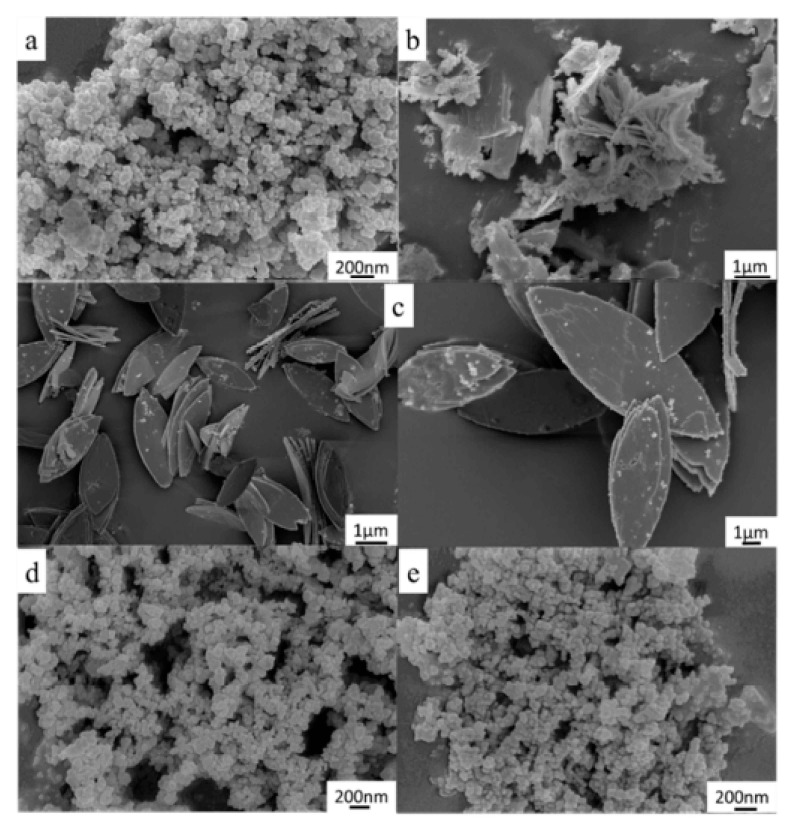
Micrographs showing the dependence of Nd_2_O_3_ nanoparticles shape on the concentration of *N*-(3-cocoamidopropyl)-betaine (CAPB), parts (**a**–**d**). Part (**e**) shows the nanoparticles fabricated in the absence of the surfactant [204]. Reprinted with permission from ref. [204]. Copright 2021 Elsevier.

To explore the application potential of a rare earth oxide in its various morphologies, Huang et al. [204] synthesized monodisperse Nd_2_O_3_ nanoparticles using ILBS as a template. The surfactants employed included (cationic) C_14_C_1_ImCl, C_8_C_1_ImCl and (zwitterionic) *N*-(3-cocoamidopropyl)-betaine (CAPB). Nd_2_O_3_ nanoparticles were prepared from its precursor NdCl₃ in the absence and presence of ILBS at concentrations greater than cmc. Nd_2_O_3_ forms multifarious shaped nanocrystals (short nanorods, nanospheres, irregular flakes, highly regular leaf-shaped to torispherical) when CAPB concentrations were changed from 1 to 20 times its cmc (=0.01 M). The short nanorods prepared in the absence of surfactant have good homogeneity with diameters of about 100 nm, (Figure 15e), changing to nanospheres with better homogeneity and an average diameter of 50 nm when the CAPB was added at its cmc (Figure 15a). Increasing the concentration of CAPB to 5 times its cmc led to a mixture of nanospheres and irregular flakes with diameters about 50 nm (Figure 15b). At [CAPB] = 10 × cmc, regular leaf-shaped Nd₂O₃ nanoparticles with lengths of 12 mm, widths of 6 mm and thicknesses of 50 nm were obtained. These NPs are composed of aggregated nanorods with lengths of about 200 nm and some nanospheres (Figure 15c). At [CAPB] = 20 × cmc, the shape of the Nd_2_O_3_ particles changed to torispherical with diameters about 50–100 nm with a certain extent agglomeration; see Figure 15d [204].

**Figure 16:**
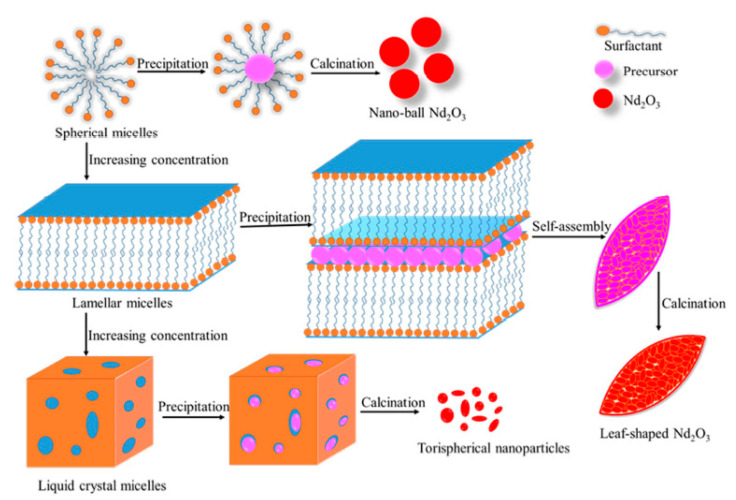
Schematic representation of the effects of surfactant concentrations on the morphologies of Nd_2_O_3_ nanoparticles. Close to the surfactant critical micelle concentration, small, spherical micelles are formed, leading after calcination, to spherical Nd_2_O_3_ nanoparticles. Micellar morphology changes at higher [surfactant] lead to the formation of leaf-shaped Nd_2_O_3_ nanoparticles [204]. Reprinted with permission from ref. [204]. Copright 2021 Elsevier.

When CAPB was replaced by C_14_C_1_ImCl, Nd_2_O_3_, NPs with different morphologies were obtained, namely leaf-shaped nanosheets and nano-blocks. Thus, different surfactants form different micellar templates, leading to different morphologies of the fabricated NPs; the surfactant with a lower cmc value (C_14_C_1_ImCl; 0.003 mol L⁻^1^) [222] forms a more stable micelles template. The authors explained the mechanism of the formation of variously shaped nanoparticles through the schematic representation shown in Figure 16 [204].

**Figure 17:**
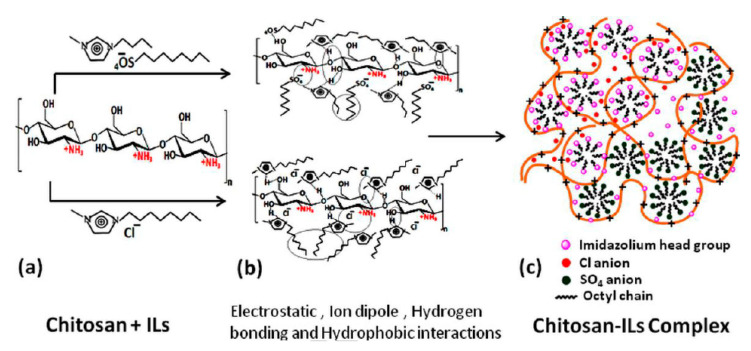
Schematic representation of the effect of surfactant anion on the formation of chitosan nanoparticles. Interactions of the chloride ion with the 1-octyl-3-methylimidazolium cations at the aggregate interface lead to the formation of larger aggregates. This is hindered in the case of the voluminous octyl sulfate anion [61]. Reprinted with permission from ref. [61]. Copright 2021 Elsevier.

Chitosan forms complexes with ILBSs (C_4_C_1_ImC_8_OSO_3_ and C_8_C_1_ImCl) above their respective cmc values, at pH = 3, i.e., where the biopolymer is protonated. The difference between these surfactants is that the hydrophobic part of the former is the anion (C_8_OSO_3_⁻), whereas it is the cation in the latter (C_8_C_1_Im). Electrostatic and hydrophobic interactions between chitosan and these ILBSs lead to the formation of positively charged spherical chitosan NPs, with sizes ranging from 300 to 600 nm from the aqueous biopolymer (0.2 wt.%) solutions. Chitosan NPs prepared using C_4_C_1_ImC_8_OSO_3_ have better sphericity and show less agglomeration than those prepared using C_8_C_1_ImCl. For the latter ILBS, the chloride counter-ions at the surface of micelles induce interactions between chitosan and C_8_C_1_ImCl complexes, leading to the agglomeration of biopolymer–micelle complexes. The relatively hydrophobic octyl sulfate anion at the micellar interface prevents the agglomeration of the chitosan-ILBS aggregate complexes; see Figure 17 [61].

**Figure 18:**
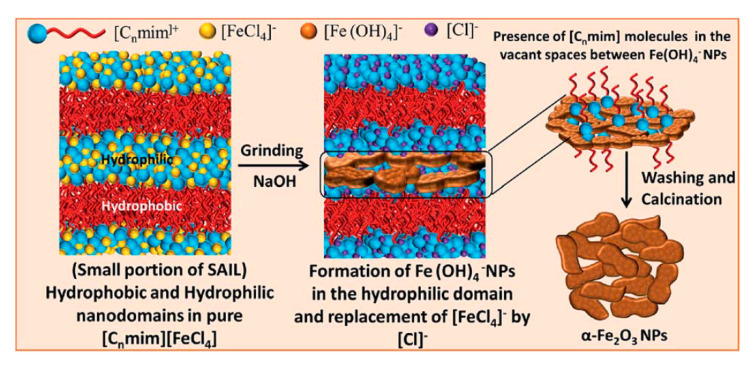
Schematic representation of the role of the nano-segregated polar and non-polar domains of the ionic liquid-based surfactant in the formation of interconnected network of α-Fe_2_O_3_ nanoparticles [205]. Reprinted with permission from ref. [205].

To limit the use of organic solvents, and to reduce the number of preparation steps, Komal et al. [205] used ILBSs with tetrachloroferrate anion, namely, 1-R-3-methylimidazolium FeCl₄, R = n-butyl, n-octyl and n-hexadecyl as the templates for the preparation of α-Fe_2_O_3_ NPs, via a grinding followed by calcination. It was observed that upon going from n-butyl to n-hexadecyl, the size of the NPs decreases. These NPs are interconnected in the form of nano-sheets, where the void spaces in the interconnected network and solution viscosity increase upon going from n-butyl to n-hexadecyl, preventing agglomeration of the NPs. At the same time, the concomitant decrease in the surface tension reduces the energy barrier to nucleation that causes an increase in the rate of nucleation as compared to the growth rate of NPs. This also decreases the size of the NPs upon going from n-butyl to n-hexadecyl. Pictorial presentation of the nano-segregated polar and non-polar domains of the ILBSs employed in this study is depicted in Figure 18. The synthesized NPs showed ILBS dependent structural, photo-physical and magnetic properties.

The fabricated α-Fe_2_O_3_ NPs were further explored as catalysts for the photo-degradation of the organic dye Rhodamine B by sunlight. The availability of the larger voids between the interconnected network influences the catalytic activity of the synthesized NPs with those fabricated using n-hexadecyl ILBSs showing the highest and n-butyl the lowest. Furthermore, ILBSs with n-hexadecyl chain length show excellent recyclability and can be used without losing their catalytic character even after four dye-degradation cycles [205].

Li et al. [206] used 1-(10-bromodecyl)-3-methylimidazolium bromide as the morphology-controlling agent to synthesize icosahedral gold NPs. These were then electrochemically deposited onto a glassy carbon electrode surface. A highly ordered and dense monolayer of the Au NPs was formed at the interface through self-assembling the 1,3-di-(3-mercaptopropyl)-imidazolium bromide IL. The prepared modified glassy carbon electrode was used as the electrochemical immunosensor for selective and sensitive determination of Squamous cell carcinoma antigen [206].

Xu et al. [207] used vesicles of the PEGylated ILBSs (surfactants with polyethylene glycol (PEG) side chain) to stabilize the Pd nanoparticles. The polyethylene glycol ether moiety was CH_3_O-(CH_2_CH_2_O)_11_-CH_2_CH_2_- and the other group was methyl, benzyl, n-octyl and n-hexadecyl, the counter ion was iodide (first ILBS) or bromide. The prepared Pd NPs in the presence of hydrazine hydrate were used as an efficient catalytic system for the chemoselective reduction of nitroarenes. ILBSs with C**₁₆** chain gave the best result with a 99% yield, as compared to no reduction in the case of the ILBSs with R = methyl. It was observed that in absence of aqueous ILBS solution, the reduction was inhibited, because of poorly stabilized Pd NPs. As observed above, the NP sizes decreased with increasing the alkyl chain length and concentration of the ILBS. The increased reaction yield is due to the smaller-sized NPs that increase the surface area, leading to their better stabilization. The authors suggested three stabilizing effects (i) electrostatic, through the cations and anions of the ILBS; (ii) steric, via protection of the NPs through the PEG chain; and (iii) chemical, due to the formation of *N*-heterocyclic carbene palladium complex between the C-2 hydrogen of the surfactant imidazolium ring [207].

A similar approach of using PEGylated GILBS was employed for the fabrication of catalytically active Pd suspension that was employed in hydrogenation. The PEGylated GILBS was synthesized by reacting 1-(n-dodecyl)imidazole with Cl(CH_2_)_2_O-(CH_2_CH_2_O)_43_-Cl to get the poly(ethylene glycol) functionalized gemini surfactant. The Pd NPs were fabricated by a treatment of palladium acetate with the surfactant solution (12 h, room temperature), followed by treatment with hydrogen (0.1 MPa) at 60 °C for 20 min. The catalyst obtained was successfully employed for the hydrogenation of several classes of organic compounds at room temperature and a pressure of 1 MPa. The following are examples of compounds that were hydrogenated with 100% product yield: styrene, cyclooctene, ethyl acrylate, allyl alcohol, nitrobenzene and 4-nitrotoluene [223].

C_10_C_1_ImBr was used as a template for fabricating hollow spherical PtCu alloy NPs (with the size of 124 ± 16 nm) supported on reduced graphene oxide (PtCu/rGO). The rGO was used as the supporting material because of its unique structure, large specific surface area and excellent electrical conductivity. The synthesized PtCu/rGO exhibited a high electrocatalytic activity and good poisoning-resistant ability during methanol oxidation in acidic medium. In order to investigate the influence of the ILBS anion on the formation of PtCu/rGO nanoparticles, three C_10_C_1_ImCH_3_CO_2_ and C_10_C_1_ImBF_4_ were tested. Among these, C_10_C_1_ImBr resulted in less NP agglomeration. Further, the impact of the alkyl chain length of the ILBSs was studied. Unlike other studies, irregularly shaped hybrid PtCu/rGO NPs were obtained when C_6_C_1_ImBr and C_14_C_1_ImBr were employed, and hollow spherical PtCu/rGO was observed when C_10_C_1_ImBr was used. This dependence was attributed to the difference in the amphiphilicity of these cations, which can produce ordered self-organized structures. The electrochemical active surface area of the fabricated supported PtCu/rGO (in m^2^ g⁻^1^) was 1.64, i.e., larger than that of commercial Pt/rGO. The material obtained was employed for the catalytic oxidation of a solution of methanol in 0.5 mol L⁻^1^ H_2_SO_4_. The high electrocatalytic activity and a good tolerance for methanol oxidation of PtCu/rGO were attributed to the unique hollow spherical nanostructures that enlarge the specific surface area and provide more active sites for the electrooxidation of methanol, and the two dimensional and nanostructures of rGO promote electron transfers during the reaction [208].

Abbaszadegan et al. [209] prepared C₁₂C₁ImCl-protected positively charged Ag NPs that were further studied as a promising disinfectant in root-canal dental treatment. Ag NPs with different surface charges (negative, neutral and positive) were synthesized, and their antibacterial activity and cytotoxicity were evaluated and compared with two widely used endodontic irrigates, namely NaOCl and chlorhexidine gluconate. Among the synthesized NPs, positively charged Ag NPs completely prevented the growth of *Enterococcus faecalis,* even after 5 min of contact time, whereas negatively charged Ag NPs started to inhibit their growth only after 1 h of contact time; neutral Ag NPs had a moderate inhibitory effect. The greater affinity of the positively charged Ag NPs to sulfur- and phosphorus-containing proteins of bacteria leads to the higher antibacterial activity of it amongst the three Ag NPs. Results of the antibacterial activity suggest that a 70-fold concentration of NaOCl was required to achieve an antibacterial activity equal to the positively charged Ag NPs. When tested for their cytotoxicity against L929 fibroblast cell lines in vitro, positively charged Ag NPs showed significantly lower cytotoxicity than the negatively charged and neutral charged Ag NPs. The positively charged Ag NPs exhibited even less cytotoxicity than the NaOCl and CHX [209].

### 4.2. Polymerization

**Figure 19:**
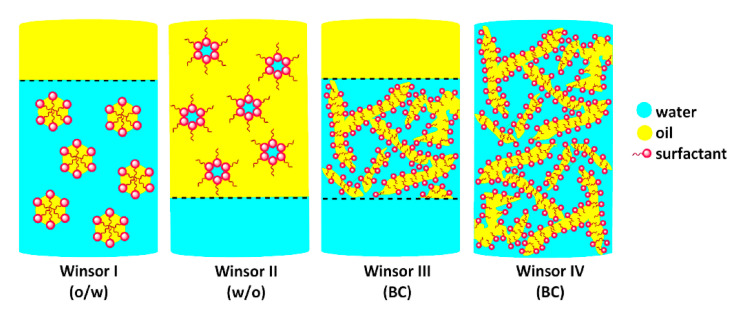
Types of microemulsions according to Windsor. Water and oil phases are colored in turquoise and yellow color, respectively. W, O and BC refer to water, oil and bicontinuous phase, respectively [3]. Reprinted with permission from ref. [3]. Copright 2021 Elsevier.

As expected, weakly surface-active ILs and ILBSs form emulsions and microemulsions (μEs), both W/O and O/W. Microemulsions are isotropic, transparent or translucent solutions, usually formed by water, oil and an amphiphile. Interest in μEs stems from the small diameters of the (W or O) nanodroplets formed (3–30 nm) and, most importantly, their thermodynamic stability, essentially because of the very low O/W interfacial tension [224,225]. Windsor [226] classified μEs into the four types, as shown in Figure 19. In Windsor type I μEs, oil nanodroplets are stabilized in an aqueous continuous phase by the surfactant, in addition to excess oil phase. The inverse situation exists in type II μEs, i.e., water nanodroplets stabilized in oil, in addition to the excess aqueous phase. In type III, the μE is composed of a bicontinuous (BC) O/W μE in equilibrium with W and O, whereas type IV is composed solely of BC phase, i.e., W and O mix in all proportions. Depending on the type of continuous pseudo-phase, μEs are classified into aqueous and nonaqueous systems. μEs have important applications because the diameter of the formed NPs, including metals, oxides, and polymers can be controlled by adjusting, e.g., the molar ratio of W and O. This, in turn, controls the diameter of the formed nanodroplets and hence that of the NPs therein.

Regarding polymerization, ILs and ILBSs were employed to form μEs that contain monomers as the “oil” component; this is usually followed by a controlled polymerization. Additionally, “polymerizable” ILBSs can be employed in latex production instead of nonpolymerizable surfactants, leading to latex with enhanced stability. Thus, μEs were prepared from oil (methyl methacrylate, MMA), water and either the IL C_4_C_1_ImBr, or the ILBS C_12_C_1_ImBr. The free-radical polymerization of MMA (by atom transfer radical polymerization; ATRP) produced polymethylmethacrylate (PMMA) NPs with diameters between 40 and 60 nm. The IL and ILBs were recycled and reused, producing PMMA NPs with reproducible size distribution, average molar mass (MM) and low polydispersity [227,228].

The polymerization of MMA was carried out at 25 °C by AGET (activators regenerated by electron transfer)-ATRP in C_4_C_1_ImBF_4_-based μE with polyoxyethylene sorbitan monooleate as surfactant; CCl_4_ as initiator; the complex FeCl_3_·6H_2_O/*N*,*N*,*N*′,*N*′-tetramethyl-1,2-ethanediamine as catalyst, and ascorbic acid as reducing agent. The polymerization kinetics showed the feature of controlled ″living″ process as evidenced by linear first-order plots. The produced PMMA had narrow polydispersity indices (1.22 to 1.35) and an average particle size <30 nm. The IL-based μEs were transparent throughout the polymerization process, whose rate increased with increasing the [MMA]/[CCl₄] molar ratio and the concentration of the surfactant [229].

**Figure 20:**
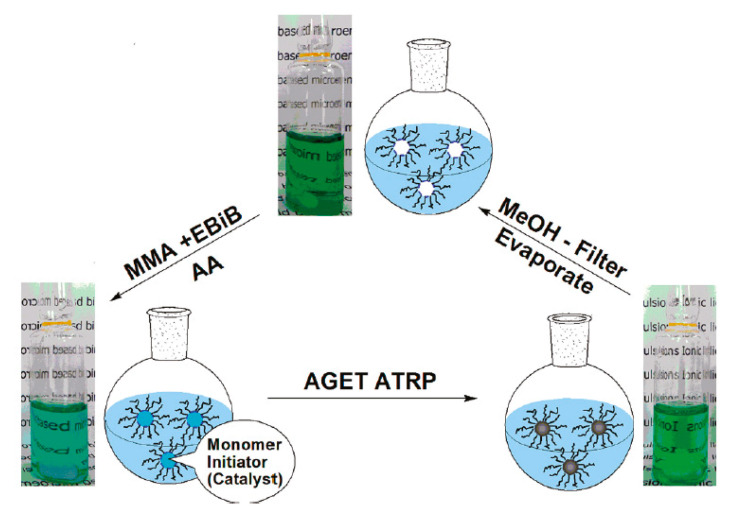
The complete cycle of activators regenerated by electron transfer- atom transfer radical polymerization) (AGET–ATRP) of MMA, polymer precipitation and microemulsion regeneration in the system C_12_C_1_ImBr/C_4_C_1_ImBF_4_/MMA. EBiB and AA refer to the polymerization initiator ethyl-2-bromo-isobutyrate and ascorbic acid, respectively [230]. Reprinted with permission from ref. [230]. Copright 2021 ACS Publications.

The use of C_12_C_1_ImBr/C_4_C_1_ImBF_4_-based μEs for AGET-ATRP polymerization of MMA was investigated. The produced PMMA NPs had a small average diameter (∼5 nm) and narrow MM distribution (*M*_w_/*M*_n_ = 1.24), probably due to the low initiation efficiency in IL/ILBS-μE polymerization. After isolation of the formed PMMA, the mixture containing the catalyst and the IL/ILBS was recycled four times with convenient results in terms of the average MM (5748 ± 398) and *M*_w_/*M*_n_, 1.24 to 1.37. Figure 20 shows the complete cycle of AGET-ATRP of MMA, polymer precipitation and microemulsion regeneration in the system C_12_C_1_ImBr/C_4_C_1_ImBF_4_/MMA [230].

The effect of ILBS (C_12_C_1_ImBr) and GILBS (C_14_Im)_2_C_4_Br_2_ on the polymerization at 60 °C of MMA in O/W μEs was investigated to delineate the effects of the molecular structure of the surfactant. The latex PMMA NPs obtained with the ILBS had a smaller diameter (30–40 nm) and higher *M*_n_ (=442,600) than that synthesized in the presence of GILBS, (50–90 nm and 262,400, respectively). This was attributed to the difference between the cmc values of the two surfactants, which were larger for C_14_C_1_ImBr. Therefore, at the same surfactant molar concentration, there is a smaller number of micelles in the C_14_C_1_ImBr μE, leading to the formation of more MMA oligomeric radicals in the bulk aqueous phase, before they adsorb the surfactant molecules [231]. A PMMA/TiO_2_/IL photocatalyst was fabricated by polymerization of MMA in μE of C_4_C_1_ImBF_4_/1-butanol/Triton X-100 nonionic surfactant (2-[4-(2,4,4-trimethylpentan-2-yl)phenoxy]ethanol), with TiO_2_ loading from 0.006 to 0.14 wt.%. For all samples, no visible phase separation was observed during and after the polymerization reaction. The recyclability of the polymerization catalyst was demonstrated through a series of subsequent polymerization reactions. The efficiency is significantly reduced, however, after the 5th polymerization cycle due to progressive surface poisoning and particle aggregation. Films were fabricated from the NPs and employed for the photodegradation of methylene blue as a model pollutant. The results indicated that the PMMA/TiO_2_ NPs are more efficient in dye elimination than pure TiO_2_. The efficiency of photooxidation first increased, then decreased as a function of increasing the TiO_2_ content of the NPs. The effect of pH on the photodegradation of MB dye was investigated; the NP efficiency was maximum at pH = 8. The reason is that at this pH, a positive charge develops on the surface of the catalyst, leading to increased NP-cationic dye electrostatic attraction that increases the photocatalytic activity [232].

The separation of aromatic/aliphatic hydrocarbons is industrially important; it is carried out by extractive distillation, azeotropic distillation and liquid–liquid extraction. The efficiency of membrane separation of these hydrocarbons is increased by using silver salt complexes adsorbed onto the membrane. On use, however, the adsorbed silver ions “leach” into the liquid medium, leading to a decreased membrane separation efficiency and selectivity. This problem can be avoided by incorporating Ag⁺ into a polymeric matrix. Thus, AgCl NPs were incorporated into MMA–acrylamide (AM) co-polymer. The latter was fabricated by co-polymerization of MMA/AM (molar ratio 3:1) in μE using C_12_C_1_ImCl. The AgCl/poly(MMA-*co*-AM) hybrid membranes were 20 ± 2 μm thick, composed of a core (AgCl; average diameter = 20 nm)-shell (MMA-*co*-AM) structure. Figure S2 shows a schematic representation of the formation of AgCl that results from the addition of an aqueous solution of AgNO**₃** to the ILBS-μE, via an anion-exchange reaction (AgNO_3_ + C_12_C_1_ImCl → AgCl + C_12_C_1_ImNO_3_); see Figure S2. AgCl was fixed into the MMA-AM co-polymer matrix via a –(H_2_N)C==O⋯⋯Ag⁺ coordination. The swelling/sorption behavior of the hybrid membranes in benzene and cyclohexane and the separation ability of these hydrocarbons were investigated. The membranes showed preferential sorption of/swelling by benzene and demonstrated better pervaporation performance than that of the membrane without Ag⁺ NPs in separating the benzene/cyclohexane mixtures [233].

**Figure 21:**
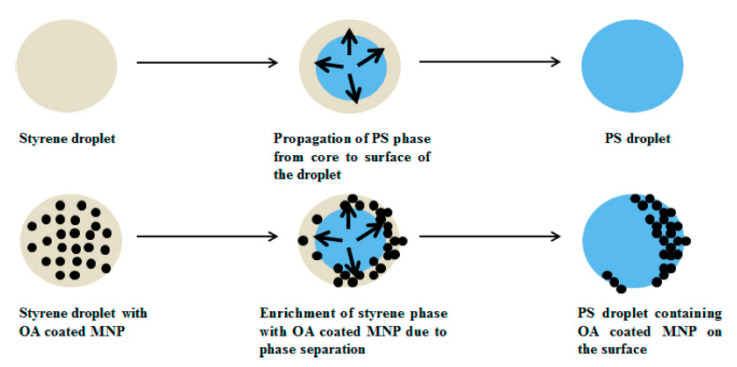
Schematic representation of ionic liquid-based surfactant-mediated fabrication of nanoparticles of polystyrene (PS) without and with magnetic properties (MNP) [234]. Reprinted with permission from ref. [234]. Copright 2021 ACS Publications.

Styrene was polymerized (free radical, RAFT) in 1-R-3-methlyimidazolium bromide-based mini-emulsions (R = C_12_ and C_16_). Polystyrene (PS) nanoparticles were obtained (average diameter 80–125 nm, depending on the experimental conditions), demonstrating the efficiency of the ILBSs. The surfactant stabilized mini-emulsions were employed to fabricate PS-based magnetic NPs, as shown in Figure 21. The enrichment of styrene phase with oleic acid (OA)-coated magnetic NPs is due to phase separation between magnetic NPs and developing PS phase during mini-emulsion polymerization of PS [234].

OA-coated magnetic NPs were fabricated by stirring an aqueous mixture of FeCl_2_ + FeCl_3_ with OA, followed by neutralization of OA with NH_4_OH aqueous solution, washing and drying of the magnetic OA-coated NPs. The magnetic PS NPs were then fabricated by styrene polymerization in the presence of OA-coated NPs. The authors assumed a preferential migration of the magnetic NPs to the PS particle surface, due to their immiscibility with the final PS phase, as shown in Figure 21. Finally, the magnetic properties of the materials were determined by vibrating sample magnetometer analysis [234].

**Figure 22:**
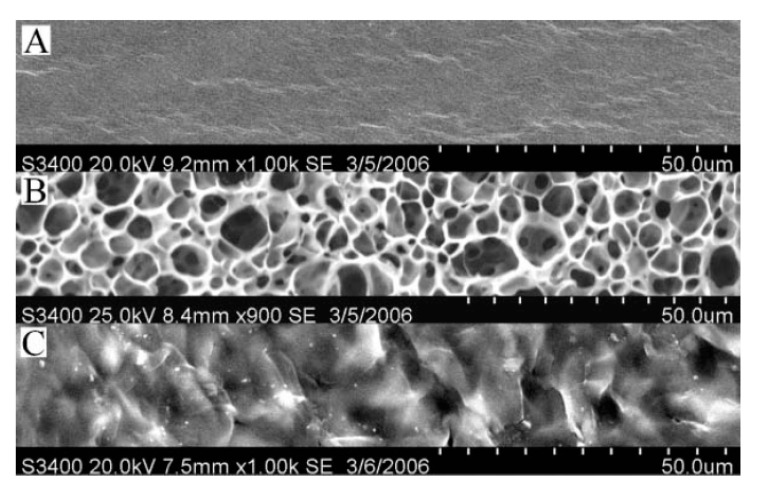
SEM images of polymers fabricated by microemulsion polymerization of MMA in the presence of the polymerizable ionic liquid-based surfactant-b. Parts (**A**–**C**) refer to polymer gel as produced, the gel after treatment with aqueous solutions of 0.1 mol L⁻^1^ of KPF_6_ and NaBr, respectively [227].

An interesting extension of the use of ILBSs in polymerization is where the surfactant itself carries a polymerizable group (usually a double-bond), leading to its inclusion in the formed polymer. Thus O/W μE-mediated polymerization of MMA was carried out using (non-polymerizable) C_12_C_1_ImBr (ILBS-a) and polymerizable 1-(2-acryloyloxyundecyl)-3-methylimidazolium bromide (ILBS-b, for structure see Figure S3). Unlike the PMMA particles produced using ILBS-a, those obtained using ILBS-b did not show aggregation, probably due to the formation of polymerized surfactant shell around the PMMA core NPs, thus rendering them more stable. The anion (Br⁻) of the polymerized surfactant can be exchanged with other, less hydrophilic and stimuli-responsive anions, e.g., BF_4_⁻, PF_6_⁻ and N(CN)_2_⁻. Figure 22 shows SEM images of the fabricated PMMA, before and after the above-mentioned anion exchange [227].

The same polymerizable surfactant-based μE was employed to fabricate PMMA NPs that were employed to obtain polymeric films. These can be used as starting material for the production of coatings whose porosity and transparency can be “fine-tuned” according to the duration of their treatment in an aqueous KPF_6_ solution; see Figure S4 in Supplementary Material. Ion-exchange with the surfactant anion (Br⁻) with BF_4_⁻, PF_6_⁻, and N(CN)_2_⁻ leads to stimuli-responsive films, due to the pairing of the anion to the imidazolium ion [235].

Anion exchange of the ILBS can be also employed to confer magnetic properties to the fabricated polymers. For example, C_12_VnImBr was homopolymerized and the anion (Br^−^) of the produced polymer was transformed into FeBrCl_3_^−^, CoBrCl_2_^−^ and MnBrCl_2_^−^ by reaction with FeCl_3_, CoCl_2_ and MnCl_2_, respectively. As expected, these polymerized ILBSs showed magnetic properties. Additionally, C_12_VnImX (X = FeBrCl_3_^−^, CoBrCl_2_^−^ and MnBrCl_2_) were copolymerized with mixtures of MMA and n-butyl acrylate to give stable latexes that showed paramagnetic behavior with weak antiferromagnetic interactions between the adjacent metal ions; i.e., they are candidates as optically transparent microwave absorbing materials [236].

The same strategy, i.e., polymerization in the presence of a colloidal stabilizer, followed by ion-exchange was employed to fabricate so-called “liquid marbles”. This term refers to liquid droplets (generally water) coated with an exterior layer of a hydrophobic material. They display non-adhesive and nonwetting behavior toward many surfaces, so that these droplets “float” on the surface of water. When liquid marbles exist in macroscale, they appear as free-flowing powders, referred to as “dry liquids” (e.g., “dry water”) because they appear as dry, solid materials rather than continuous fluids. Thus, styrene, and mixtures of MMA and benzyl methylacrylate were polymerized (free radical emulsion polymerization) in the presence of cationic surfactants, e.g., CTACl, and poly(1-vinyl)-3-methylimidazolium bromide as particle stabilizers. The fabricated liquid marbles (diameter up to 500 nm) can have magnetic properties by polymerization in the presence of magnetite (Fe_3_O_4_). The same strategy was employed to fabricate fluorescent liquid marbles, namely, emulsion polymerization in the presence of fluorescein *O*-methacrylate or acrylate functionalized Rhodamine B1. The fabricated liquid marbles can be flocculated by ion exchange of the stabilizer anions (Br^−^ or Cl>^−^) with (hydrophobic) bis(trifluoromethanesulfonyl)-imide. The properties (magnetic and fluorescent) of the above-mentions materials can be exploited in different applications, e.g., gas and pH sensing, microreactors, microfluidics, biotechnology, drug delivery and also cosmetics and personal care products [237,238].

## 5. Conclusions and Perspectives

We identify molecular structure versatility as the main reason for the sustained interest in single- and multiple-chain ILBSs. This offers a window of opportunities for diverse applications, including the fabrication of NPs thfighuat are employed, inter alia, in catalysis, decontamination and drug delivery. Dimeric and polymeric ILBSs have an additional structural dimension, the spacer, whose length can be varied as required, and may contain a heteroatom. Another interesting class is biamphiphilic surfactants, because the electrostatic and hydrophobic interactions that lead to aggregate formation can be “fine-tuned” by controlling the length and hydrophobicity of both surfactant ions; see Figure 8. Table 1, Table S1, Table 2, Table S2 show the recent data available (last 10 years) on the adsorption at the water/air interface and micelle formation by single- and multiple-chain ILBSs. This should help in choosing not only the molecular structure of the ILBS, but also the optimum surfactant concentration for the intended application. The values of *α*_mic_ are related to the surface potential of the micelle and hence help in applications where electrostatic interactions substrate-micelle are important. Among many applications, we chose those relying on the use of ILBSs as soft templates for the fabrication of NPs and polymers. We hope that our effort highlights these points and serves to increase the awareness of the enormous potential of ILBSs in science and technology.

Figure 2 shows the sustained and expanded interest in ILs and ILBSs. In this review, we focused on their use in the fabrication of NPs, both metallic and polymeric. Although it is outside the scope of the present review to cover all important applications of ILBSs, it is worthwhile to mention other applications, including their use in enhanced oil recovery, e.g., by flooding with μEs [239,240], and in analytical chemistry, including liquid–liquid extraction [241], voltammetry and amperometry as organic electrolytes for carrying out electrochemical processes [242], solid-phase microextraction [243] and in chromatography [244]. The ease with which the properties of weakly surface-active ILs and ILBSs can be fine-tuned to the researcher’s need means that the ascending curves shown in Figure 2 is likely to continue in the future. The somewhat “exotic” uses of ILs by NASA are just an example (www.nasa.gov/oem/ionicliquids (accessed on 29 March 2021)).

## Figures and Tables

**Table 1:** Literature data on aqueous solutions of ionic liquid-based surfactants at 25 °C. Adsorption and micellar parameters calculated from surface tension data.

Entry	Cation ^1^	Anion ^2^	cmc × 10^3^ (mol L^−1^) ^3^	γ_cmc_ (mN m^−1^) ⁴	Π_cmc_ (mN m^−1^) ⁵	*Γ*_max_ (mol m^−2^) ⁶	*A*_min_ (Å^2^) ⁷	p*C*_20_ ⁸	Δ*G*^0^_ads_ (kJ mol^−1^) ⁹
**Cationic ILBSs**									
1	C_8_C_1_Im^+^	Cl^−^	116 [58], 101.7 [59], 190 [60], 108.9 [61], 170.2 [62]	28.3 [59], 33.5 [61], 37.3 [62]	43.3 [59], 32.2 [60], 38.5 [61]	2.682 [58], 1.6 [59], 1.52 [61], 1.24 [62]	104 [59], 84 [60], 109 [61], 133 [62]	1.8 [59], 1.67 [60], 1.92 [61]	−43.33 [58], −47.6 [61], −24.6 [62]
2		Br^−^	121 [45], 120.0 [59], 170 [63], 119.29 [64]	41 [45], 28.7 [59], 41.3 [63], 29.33 [64]	44.9 [59], 42.87 [64]	2.7 [59], 3.065 [64]	60 [59], 124 [63], 53 [64]	1.8 [59]	−36.5 [64]
3		I^−^	94.9 [59]	28.2 [59]	44.4 [59]	1.4 [59]	117 [59]	2.0 [59]	
4		C_1_SO_3_^−^	220 [65]		29.0 [65]			1.0 [65]	
5		C_4_SO_3_^−^	140 [65]		28.5 [65]			1.6 [65]	
8	C_10_C_1_Im^+^	Cl^−^	39.90 [42], 45 [60], 40.00 [66], 40.0 [67], 40 [68], 40 [69], 27.7 [70]	27.3 [66]	33.7 [60], 44.5 [66]	1.9 [66], 1.84 [67], 1.84 [68], 1.84 [69]	85 [42], 92 [60], 85 [66], 90 [67], 90 [68], 90 [69]	2.55 [60], 2.5 [66], 2.60 [68]	−30.16 [42], −48.55 [67], −48.00 [68], −48.55 [69]
9		Br^−^	20 [45], 33 [63], 36.7 [71], 27.24 [72], 39.6 [73]	39 [45], 39.1 [63], 37.3 [71], 48.95 [72], 36.04 [73]	35.5 [71], 23.02 [72], 33.37 [73]	1.82 [71], 1.75 [72], 1.71 [73]	91 [63], 57.6 [74], 91.4 [71], 94.61 [72], 97.01 [73]	1.61 [72]	
10		C_1_SO_3_^−^	60 [65]		28.5 [65]			1.5 [65]	
12	C_12_C_1_Im^+^	Cl^−^	13.17 [42], 14.80 [66], 13.8 [70], 16.8 [75], 13.86 [76], 13.25 [77], 11.62 [78]	38.7 [66], 38.4 [75], 30.45 [76]	33.6 [66], 41.55 [76], 28.6 [77]	2.3 [66], 2.91 [75], 2.04 [76]	72 [42], 72 [66], 57 [75], 81.39 [76], 72 [77]	2.4 [66], 2.16 [75], 2.72 [76]	−32.03 [42]
13		Br^−^	4.3 [45], 9.0 [63], 9.19 [72], 10.6 [75], 9.29 [77], 10.35 [79], 8.22 [80], 11.21 [81], 8.73 [82], 9.68 [83], 9.0 [84], 9 [85]	35 [45], 37.2 [63], 46.71 [72], 36.8 [75], 38.96 [80], 42.9 [81], 38.68 [82], 37.40 [83]	25.26 [72], 29.3 [77], 33.06 [80], 33.94 [82]	1.80 [72], 3.03 [75], 4.48 [79], 3.36 [80], 3.095 [82]	67 [63], 92.49 [72], 64 [74], 55 [75], 71 [77], 37.1 [79], 49.42 [80], 53.7 [82]	2.35 [72], 2.16 [75], 3.06 [79], 2.54 [80], 2.45 [82]	
14		I^−^	4.6 [75], 4.76 [77]	31.7 [75]	37.7 [77]	4.47 [75]	37 [75], 62 [77]	2.80 [75]	
15		C_1_SO_3_^−^	14 [65]		28.5 [65]			2.1 [65]	
17	C_14_C_1_Im^+^	Cl^−^	2.98 [42], 3.10 [70], 3.63 [86]	34.15 [86]	36.65 [86]	2.25 [86]	56 [42], 74 [86]	2.89 [86]	−33.81 [42], −55.55 [86]
18		Br^−^	1.9 [63], 2.69 [83], 2.76 [87], 2.6 [88], 2.8 [89]	37.2 [63], 37.70 [83], 37 [88], 39.2 [89]	41.4 [87], 33.8 [89]	1.74 [87], 1.26 [88], 1.96 [89]	67 [63], 64.8 [74], 95 [87], 132 [88], 84.7 [89]	3.6 [87], 3.03 [88], 3.33 [89]	−47.48 [87]
19	C_16_C_1_Im^+^	Cl^−^	0.87 [42], 1.14 [66], 0.89 [90], 0.87 [91]	37.0 [66], 40.9 [91]	34.8 [66], 28.8 [90]	3.4 [66], 2.06 [90], 3.4 [91]	49 [42], 49 [66], 80 [90], 49 [91]	3.2 [66], 3.4 [90], 3.39 [91]	−35.23 [42], −35.64 [91]
20		Br^−^	0.8 [45], 0.610 [72], 0.78 [79], 0.51 [83], 0.55 [89], 0.71 [91], 0.566 [92]	41 [45], 44.53 [72], 37.41 [83], 39.1 [89], 38.7 [91], 38.98 [92]	27.44 [72], 33.9 [89], 33.05 [92]	2.00 [72], 4.20 [79], 2.03 [89], 3.0 [91], 2.06 [92]	83.14 [72], 39.6 [79], 81.6 [89], 54 [91], 80.7 [92]	3.42 [72], 3.85 [79], 3.78 [89], 3.55 [91]	−36.83 [91]
21	C_18_C_1_Im^+^	Cl^−^	0.40 [66]	42.0 [66]	29.8 [66]	3.7 [66]	45 [66]	3.6 [66]	
22	C_12_C_2_Im^+^	Br^−^	6.40 [80]	38.09 [80]	33.93 [80]	3.09 [80]	53.74 [80]	2.70 [80]	
23	C_16_C_2_Im^+^	Cl^−^	0.88 [91]	35.4 [91]		2.6 [91]	63 [91]	3.62 [91]	−38.29 [91]
24		Br^−^	0.55 [91], 0.26 [93]	39.8 [91], 32.2 [93]	40.3 [93]	2.7 [91], 2.58 [93]	61 [91], 64.2 [93]	3.65 [91], 4.22 [93]	−38.19 [91], −66.32 [93]
25	C_10_VnIm^+^	Br^−^	27.20 [93]	34.5 [93]	37.3 [93]	1.86 [93]	89.3 [93]	2.44 [93]	−50.60 [93]
26	C_12_VnIm^+^	Br^−^	7.00 [93]	34.1 [93]	38.4 [93]	2.03 [93]	81.8 [93]	2.80 [93]	−56.31 [93]
28	C_14_VnIm^+^	Br^−^	1.85 [93]	33.8 [93]	38.6 [93]	2.18 [93]	76.2 [93]	3.40 [93]	−61.30 [93]
29	C_16_VnIm^+^	Br^−^	0.60 [91], 0.48 [93]	37.7 [91], 33.5 [93]	39.1 [93]	3.1 [91], 2.53 [93]	53 [91], 65.7 [93]	3.63 [91], 4.00 [93]	−37.09 [91], −65.47 [93]
30	C_12_C_3_Im^+^	Br^−^	5.05 [80]	37.56 [80]	34.46 [80]	2.20 [80]	75.48 [80]	2.85 [80]	
31	C_16_C_3_Im^+^	Cl^−^	0.71 [91]	35.2 [91]		2.2 [91]	75 [91]	3.82 [91]	−40.78 [91]
32		Br^−^	0.44 [91]	39.7 [91]		2.3 [91]	73 [91]	3.80 [91]	−40.46 [91]
33	C_16_AlIm^+^	Br^−^	0.51 [91]	38.7 [91]		2.6 [91]	63 [91]	3.74 [91]	−38.90 [91]
34	C_8_C_4_Im^+^	Br^−^	41 [45], 75.8 [94]	40 [45], 33.2 [94]	37 [94]	1.3 [94]	126.9 [94]	2.3 [94]	−51.1 [94]
35	C_10_C_4_Im^+^	Br^−^	6.3 [45]	36 [45]					
36	C_12_C_4_Im^+^	Br^−^	2.4 [45], 3.68 [80], 5.3 [94]	38 [45], 34.92 [80], 33.0 [94]	37.10 [80], 37.2 [94]	2.02 [80], 2.1 [94]	82.21 [80], 76.9 [94]	3.18 [80], 2.9 [94]	−53.2 [94]
37	C_16_C_4_Im^+^	Cl^−^	0.50 [91]	38.0 [91]		2.1 [91]	80 [91]	3.89 [91]	−41.86 [91]
38		Br^−^	0. 1 [45], 0.40 [91]	45 [45], 40.3 [91]		1.9 [91]	86 [91]	3.92 [91]	−42.73 [91]
39	C_16_C_5_Im^+^	Cl^−^	0.35 [91]	39.6 [91]		1.6 [91]	106 [91]	4.15 [91]	−46.37 [91]
40	C_8_C_8_Im^+^	Br^−^	5.6 [45], 8.0 [95]	32 [45], 25.9 [95]					
41	C_12_C_12_Im^+^	Br^−^	0.1 [45]	28 [45]					
42	C_10_C_1_C_1_Im^+^	Br^−^	43.0 [96]		30.9 [96]	1.7 [96]	99.4 [96]		
43	C_12_C_1_C_1_Im^+^	Cl^−^	12.27 [97]	31.21 [97]	40.79 [97]	0.95 [97]	1.75 [97]	2.75 [97]	−78.78 [97]
44	C_10_C_1_C_10_Im^+^	Cl^−^	1.23 [98]	32.7 [98]		1.98 [98]	83.5 [98]		−46.66 [98]
45	C_8_Py^+^	Cl^−^	181 [99]	36.84 [99]	34.6 [99]	1.70 [99]	98 [99]	1.6 [99]	
46		Br^−^	180 [63]	41.9 [63]			66 [63]		
48	C_10_Py^+^	Cl^−^	65.5 [100]						
49		Br^−^	30 [63]	40.7 [63]			61 [63]		
50	C_11_Py^+^	Br^−^	19.5 [101]						
51	C_12_Py^+^	Cl^−^	14.0 [101]						
52		Br^−^	9.3 [63]	39.3 [63]			71 [63]		
53	C_13_Py^+^	Br^−^	4.57 [101]						
54	C_14_Py^+^	Cl^−^	3.20 [102]						
55		Br^−^	2.2 [63], 2.65 [101]	38.0 [63]			86 [63]		
56	C_16_Py^+^	Cl^−^	0.99 [103]	49 [103]	23.0 [103]	1.17 [103]	142.0 [103]	3.00 [103]	−25.7 [103]
57		Br^−^	0.62 [101], 0.9 [103]	49 [103]	23.0 [103]	0.91 [103]	142 [103]	3.03 [103]	−25.70 [103]
58	C_8_-(*o*-C_1_)Py^+^	Cl^−^	166 [99]	31.80 [99]	40.1 [99]	1.67 [99]	99 [99]	3.0 [99]	
63	C_8_-(*m*-C_1_)Py^+^	Cl^−^	170 [99]	32.38 [99]	39.2 [99]	1.71 [99]	97 [99]	1.9 [99]	
65	C_10_-(*m*-C_1_)Py^+^	Cl^−^	45 [85]		27.9 [85]		121 [85]	1.62 [85]	
66	C_12_-(*m*-C_1_)Py^+^	Cl^−^	13 [85]		27.9 [85]		108 [85]	2.22 [85]	
67		Br^−^	10 [85], 9.83 [104]	39.2 [104]	32.9 [104]	1.84 [104]	90.3 [104]	2.8 [104]	−55.00 [104]
68	C_14_-(*m*-C_1_)Py^+^	Cl^−^	3.1 [85]		28.0 [85]		92 [85]	2.82 [85]	
69		Br^−^	2.26 [104]	38.8 [104]	33.3 [104]	2.09 [104]	79.4 [104]	3.1 [104]	−58.39 [104]
70	C_16_-(*m*-C_1_)Py^+^	Cl^−^	0.8 [85]		27.9 [85]		80 [85]	3.39 [85]	
71		Br^−^	0.508 [104]	37.6 [104]	34.5 [104]	2.38 [104]	69.7 [104]	3.8 [104]	−63.00 [104]
72	C_18_-(*m*-C_1_)Py^+^	Cl^−^	0.3 [85]		27.8 [85]		76 [85]	3.87 [85]	
73	C_8_-(*p*-C_1_)Py^+^	Cl^−^	175 [99]	31.00 [99]	40.7 [99]	1.65 [99]	101 [99]	2.2 [99]	
79	C_10_C_1_Pn^+^	Br^−^	31 [105]	28.6 [105]		3.8 [105]	44 [105]		
80	C_12_C_1_Pn^+^	Br^−^	16 [105]	25.1 [105]		4.4 [105]	38 [105]		
81	C_14_C_1_Pn^+^	Br^−^	7.4 [105]	28.7 [105]		4.5 [105]	37 [105]		
82	C_16_C_1_Pn^+^	Br^−^	3.3 [105]	31.2 [105]		5.2 [105]	32 [105]		
83	C_18_C_1_Pn^+^	Br^−^	1.5 [105]	32.7 [105]		4.4 [105]	38 [105]		
86	C_12_C_1_Pyrro^+^	Cl^−^	19.60 [106]	34.4 [106]	36.6 [106]	2.4 [106]	69 [106]	2.3 [106]	
87		Br^−^	15 [107], 13.5 [108]	42.4 [108]	30.3 [108]	3.03 [108]	54.8 [108]		
88	C_14_C_1_Pyrro^+^	Br^−^	3.30 [108]	42.7 [108]	30.0 [108]	3.55 [108]	46.8 [108]		
89	C_16_C_1_Pyrro^+^	Br^−^	0.860 [108], 0.72 [109]	41.2 [108], 36.7 [109]	31.5 [108]	3.67 [108]	45.2 [108], 51 [109]		
90	C_18_C_1_Pyrro^+^	Cl^−^	0.42 [66]	36.5 [66]	35.3 [66]	3.5 [66]	48 [66]	3.7 [66]	
92	C_8_C_4_Pyrro^+^	Br^−^	150 [107]						
93	C_12_C_4_Pyrro^+^	Br^−^	6 [107]						
98	C_12_C_1_Pip^+^	Cl^−^	19.79 [110]	36.5 [110]	35.3 [110]	2.4 [110]	68.5 [110]	3.35 [110]	
99		Br^−^	11 [85], 11.83 [111]	41.43 [111]	31.57 [111]	2.31 [111]	71.82 [111]	2.33 [111]	
100	C_14_C_1_Pip^+^	Br^−^	3.22 [111]	41.23 [111]	31.77 [111]	2.35 [111]	70.65 [111]	2.90 [111]	
101	C_16_C_1_Pip^+^	Br^−^	0.68 [109], 0.73 [111]	37.3 [109], 41.14 [111]	31.86 [111]	2.63 [111]	61 [109], 63.22 [111]	3.51 [111]	
102	C_18_C_1_Pip^+^	Cl^−^	0.45 [66]	37.7 [66]	34.1 [66]	3.3 [66]	50 [66]	3.8 [66]	
103	C_16_C_1_Aze^+^	Br^−^	0.590 [109]	37.9 [109]			65 [109]		
104	C_16_C_1_Azo^+^	Br^−^	0.51 [109]	38.8 [109]			75 [109]		
105	C_10_C_1_Mor^+^	Br^−^	30 [112]						
106	C_12_C_1_Mor^+^	Cl^−^	21.80 [106]	34.6 [106]	36.4 [106]	2.5 [106]	66 [106]	2.3 [106]	
107		Br^−^	9.6 [112]						
108	C_14_C_1_Mor^+^	Br^−^	4.0 [112], 2.93 [113], 4.10 [114]		41.2 [113]	2.79 [113]	59 [113]	3.2 [113]	−15.4 [113]
109	C_16_C_1_Mor^+^	Br^−^	0.74 [109], 1.0 [112], 1.02 [113], 1.00 [114]	29.9 [109]	38.5 [113]	2.69 [113], 1.82 [114]	71 [109], 61 [113], 91 [114]	3.6 [113]	−9.5 [113], −42 [114]
110	C_18_C_1_Mor^+^	Br^−^	0.33 [112]						
111	C_8_Gu^+^	Cl^−^	75 [53]	24.5 [53]		4.60 [53]	36.2 [53]		
112	C_10_Gu^+^	Cl^−^	22 [53]	23.5 [53]		3.78 [53]	44.2 [53]		
113	C_12_Gu^+^	Cl^−^	5.5 [53]	24.1 [53], 24.5 [115]		3.93 [53], 4.54 [115]	42.3 [53], 36.6 [115]		
115	C_8_C_1_C_1_Gu^+^	Cl^−^	7.2 [53]	35.4 [53]		3.66 [53]	45.4 [53]		
116	C_10_C_1_C_1_Gu^+^	Cl^−^	2.1 [53]	33.3 [53]		3.40 [53]	48.9 [53]		
117	C_12_C_1_C_1_Gu^+^	Cl^−^	0.67 [53]	32.5 [53]		3.05 [53]	54.5 [53]		
118	C_8_Ph_3_P^+^	Br^−^	32 [116]						
120	C_10_Ph_3_P^+^	Br^−^	6.1 [116], 9.0 [117], 6.60 [118]	40.00 [118]					
122	C_12_Ph_3_P^+^	Br^−^	2.0 [116], 1.51 [118], 2.35 [119], 2.00 [120], 2.00 [121]	40.80 [118], 46.0 [120]	26.5 [120], 32.4 [121]	2.24 [119], 1.36 [121]	74 [119], 122 [121]	3.01 [120]	−60.2 [119], −64.2 [121]
123	C_14_Ph_3_P^+^	Br^−^	0.33 [116], 0.61 [118], 0.61 [119], 0.60 [122]	40.60 [118], 41.3 [122]		1.88 [119]	88 [119], 82.2 [122]		−56.0 [119]
124	C_16_Ph_3_P^+^	Br^−^	0.10 [116], 0.15 [118], 0.24 [119], 0.14 [121], 0.15 [123], 0.10 [124], 0.10 [125]	40.25 [118]	25.8 [121], 27.2 [123], 31.4 [124]	1.02 [119], 1.38 [121], 1.39 [123], 1.40 [124]	163 [119], 120 [121], 119 [123], 118.0 [124]		−75.3 [119], −71.1 [121], −51.3 [123], −58.9 [124]
125	C_18_Ph_3_P^+^	Br^−^	0.018 [116]						
**Anionic ILBSs**									
127	(CH_3_)_4_N^+^	AOT^−^	2.90 [126], 2.48 [127]	29.4 [127]		1.60 [126]	104 [126], 96 [127]		
129	(C_2_H_5_)_4_N^+^	AOT^−^	2.45 [126], 2.07 [127]	28.7 [127]		1.43 [126]	116 [126], 101 [127]		
130	(C_3_H_7_)_4_N^+^	C_12_SO_4_^−^	1.46 [127]	31.8 [127]			67 [127]		
131		AOT^−^	0.97 [126], 1.27 [127]	26.1 [127]		1.71 [126]	97 [126], 96 [127]		
132	(C_4_H_9_)_4_N^+^	C_8_SO_4_^−^	24.5 [128]	32.0 [128]		3.75 [128]			
133		C_10_SO_4_^−^	4.17 [128]	31.6 [128]		4.03 [128]			
134		C_12_SO_4_^−^	0.525 [128]	31.2 [128]		4.47 [128]			
135		C_14_SO_4_^−^	0.26 [129]			5.43 [129]	31 [129]		
136		AOT^−^	0.77 [126]			1.63 [126]	102 [126]		
137	C_4_Py^+^	DBS^−^	0.92 [130]	29.69 [130]	42.31 [130]	2.31 [130]	72.08 [130]	3.92 [130]	
138	C_4_C_1_Pyrro^+^	C_12_SO_4_^−^	2.7 [131]	34.3 [131]	37.9 [131]	2.27 [131]	74 [131]	3.5 [131]	
139	C_4_C_1_Im^+^	C_8_SO_3_^−^	135 [65]		40.0 [65]			1.6 [65]	
140		C_12_SO_3_^−^	4.4 [132]	36.9 [132]	35.9 [132]	1.14 [132]	145 [132]	3.05 [132]	
141		C_8_SO_4_^−^	33.4 [61], 23.0 [62], 34.9 [133], 30.5 [134], 30 [135], 34.5 [136], 31.9 [137], 30.0 [138]	26.1 [61], 30.5 [62], 26.1 [133], 29.6 [134], 31.5 [135], 30.3 [136], 32.8 [137], 34.5 [138]	45.9 [61], 45.9 [133], 42.4 [134], 41.0 [136], 37.5 [138]	2.44 [61], 1.63 [62], 1.9 [133], 2.18 [134], 1.87 [135], 1.39 [136], 2.079 [138]	68 [61], 102 [62], 87.1 [133], 76 [134], 89 [135], 12 [136], 69 [137], 131 [138]	2.60 [61], 2.5 [133], 2.56 [135], 1.52 [138]	−49.7 [61], −38.7 [62], −49.5 [134]
142		C_10_SO_4_^−^	8.8 [139]	34.7 [139]	37.9 [139]	2.81 [139]	59 [139]	2.7 [139]	
143		C_12_SO_4_^−^	1.8 [131], 2.4 [133], 2.30 [140], 1.9 [141], 1.84 [142]	31.9 [131], 34.4 [133], 32.9 [140], 31.90 [142]	40.3 [131], 37.6 [133], 39.90 [142]	2.53 [131], 2.4 [133], 2.48 [142]	66 [131], 67.8 [133], 56 [140], 67 [142]	3.4 [131], 3.3 [133], 3.82 [142]	
144		C_14_SO_4_^−^	0.5 [139]	30.5 [139]	42.1 [139]	1.66 [139]	10 [139]	4.2 [139]	
145		DBS^−^	1.08 [130]	29.18 [130]	42.82 [130]	2.09 [130]	79.86 [130]	3.94 [130]	
146		AOT^−^	1.78 [140]	25.7 [140]			86 [140]		
147		TC^−^	0.55 [140]	24.8 [140]			111 [140]		
148	C_5_C_1_Im^+^	C_12_SO_4_^−^	1.6 [143]	57.86 [143]	13.64 [143]	0.96 [143]	173.4 [143]		−59.23 [143]
149		DBS^−^	0.32 [144]	30.92 [144]	42.38 [144]	1.91 [144]	86.76 [144]	−1.03 [144]	
150	C_6_C_1_Im^+^	C_8_SO_4_^−^	14.2 [133], 18.6 [137]	25.6 [133], 30.3 [137]	45.2 [133]	1.9 [133]	84.7 [133], 66 [137]	2.9 [133]	
151		C_12_SO_4_^−^	1.1 [133], 0.8 [139]	27.1 [133], 30.0 [139]	44.9 [133], 42.6 [139]	2.4 [133], 2.08 [139]	68.5 [133], 80 [139]	4.1 [133], 4.0 [139]	
152	C_7_C_1_Im^+^	DBS^−^	0.12 [144]	34.21 [144]	39.09 [144]	1.35 [144]	122.64 [144]	−1.15 [144]	
**Biamphiphilic ILBSs**									
153	C_8_C_1_Im^+^	C_8_SO_3_^−^	12 [65]		43.7 [65]			2.9 [65]	
154		C_8_SO_4_^−^	4.1 [133]	24.4 [133]	47.6 [133]	2.5 [133]	66.0 [133]	3.3 [133]	
155		C_12_SO_4_^−^	0.4 [133], 0.3 [139]	26.0 [133], 26.9 [139]	46.0 [133], 45.7 [139]	2.4 [133], 2.33 [139]	68.5 [133], 71 [139]	4.3 [133], 4.5 [139]	
156	C_10_C_1_Im^+^	C_12_SO_4_^−^	0.1 [139]	25.4 [139]	47.2 [139]	2.36 [139]	70 [139]	5.0 [139]	
157	C_16_Py^+^	C_8_SO_3_^−^	54 [145]						
158		C_8_SO_4_^−^	24 [145]						
162	C_16_(CH_3_)_3_N^+^	C_8_SO_3_^−^	88 [145]						
163		C_8_SO_4_^−^	26 [145]						

^1^ Abbreviations: Imidazolium (Im⁺), pyridinium (Py⁺), 2-pyrrolidinonium (Pn⁺), pyrrolidinium (Pyrro⁺), piperidinium (Pip⁺), azepanium (Aze⁺), azocanium (Azo⁺), morpholinium (Mor⁺), guanidinium (Gu⁺), ammonium (N⁺) and phosphonium (P⁺). ^2^ Acronyms: DBS, AOT, and TC refer to dodecylbenzene sulfonate, bis (2-ethylhexyl) sulfosuccinate, and aerosol-OT trichain analog, respectively. ^3^ Critical micelle concentration (cmc) from surface tension measurements. ⁴ Surface tension at cmc. ⁵ Surface pressure at cmc. ⁶ Surface excess concentration at the interface. ⁷ Minimum area per molecule at the water/air interface. ⁸ Surface tension reduction efficiency (by 20 mN m⁻^1^). ⁹ Gibbs free energy of surfactant adsorption at the water/air interface.

**Table 2:** Literature data on aqueous solutions of gemini ionic liquid-based surfactants at 25 °C. Adsorption and micellar parameters calculated from surface tension data. All surfactants have two bromides as counterions, except for entries 54 and 55, which have three bromides as counterions.

Entry	Cation ^1^	cmc × 10^3^ (mol L^−1^)	γ_cmc_ (mN m^−1^)	Π_cmc_ (mN m^−1^)	*Γ*_max_ (mol m^−2^)	*A*_min_ (Å^2^)	p*C*_20_	Δ*G*^0^_ads_ (kJ mol^−1^)
1	(C_12_Im)_2_C_2_)^2+^	0.55 [156]	33.6 [156]		1.26 [156]	135 [156]	4.54 [156]	
2	(C_16_Im)_2_C_2_)^2+^	0.0341 [157]						
3	(C_16_Im)_2_C_3_)^2+^	0.0048 [157]						
4	(C_10_Im)_2_C_4_)^2+^	4.50 [43], 2 [45]	35.2 [43], 35 [45]		1.25 [43]	133 [43]	3.14 [43]	
5	(C_12_Im)_2_C_4_)^2+^	0.72 [43], 0.76 [158]	35.7 [43], 35.0 [158]	37.8 [43]	1.19 [43], 16.30 [158]	140 [43], 101.43 [158]	3.94 [43]	−50.93 [158]
6	(C_14_Im)_2_C_4_)^2+^	0.10 [43]	37.2 [43]		0.88 [43]	188 [43]	5.04 [43]	
7	(C_16_Im)_2_C_4_)^2+^	0.0222 [157]						
8	(C_16_Im)_2_C_5_)^2+^	0.0269 [157]						
9	(C_12_Im)_2_C_6_)^2+^	0.78 [156]	39.5 [156]		1.16 [156]	143 [156]	3.73 [156]	
10	(C_16_Im)_2_C_6_)^2+^	0.0501 [157]						
11	(C_4_Im)_2_C_8_)^2+^	32.3 [45]	47 [45]					
12	(C_16_Im)_2_C_8_)^2+^	0.0512 [157]						
13	(C_1_Im)_2_C_10_)^2+^	14.9 [45]	38 [45]					
14	(C_4_Im)_2_C_10_)^2+^	11.7 [45]	38 [45]					
15	(C_16_Im)_2_C_10_)^2+^	0.0607 [157]						
16	(C_4_Im)_2_C_12_)^2+^	7.2 [45]	46 [45]					
17	(C_10_Im)_2_C_12_)^2+^	0.6 [45]	48 [45]					
18	(C_16_Im)_2_C_12_)^2+^	0.0619 [157]						
19	((C_12_SMeIm)_2_C_2_)^2+^	0.32 [55]	39.7 [55]		2.60 [55]	63 [55]	3.93 [55]	−45.57 [55]
20	((C_14_SMeIm)_2_C_2_)^2+^	0.072 [55]	42.9 [55]		2.12 [55]	78 [55]	4.53 [55]	−47.80 [55]
22	((C_12_SMeIm)_2_C_3_)^2+^	0.26 [55]	40.7 [55]		2.13 [55]	77 [55]	4.06 [55]	−48.21 [55]
23	((C_14_SMeIm)_2_C_3_)^2+^	0.063 [55]	45.8 [55]		3.09 [55]	53 [55]	4.42 [55]	−42.31 [55]
25	((C_12_SMeIm)_2_C_4_)^2+^	0.22 [55]	40.8 [55]		2.06 [55]	80 [55]	4.12 [55]	−47.51 [55]
26	((C_14_SMeIm)_2_C_4_)^2+^	0.058 [55]	46.6 [55]		3.10 [55]	53 [55]	4.39 [55]	−42.82 [55]
28	((C_12_OHIm)_2_C_3_)^2+^	0.72 [159]	30.0 [159]		2.53 [159]	65 [159]	3.91 [159]	−49.67 [159]
29	((C_12_OHIm)_2_C_4_)^2+^	0.76 [159]	28.1 [159]		2.33 [159]	71 [159]	4.11 [159]	−50.63 [159]
30	((C_12_OHIm)_2_C_5_)^2+^	1.02 [159]	32.9 [159]		2.29 [159]	72 [159]	3.72 [159]	−52.46 [159]
31	((C_12_OHIm)_2_C_6_)^2+^	1.07 [159]	35.2 [159]		2.98 [159]	55 [159]	3.44 [159]	−44.78 [159]
32	((C_12_OHIm)_2_C_8_)^2+^	1.14 [159]	37.6 [159]		1.90 [159]	87 [159]	3.58 [159]	−49.45 [159]
33	((C_12_)_3_N)_2_C_2_)^2+^	1.995 [160] ^2^		39 [160]	0.769 [160]	251.7 [160]	5.1 [160]	−20.19 [160]
34	((C_12_)_3_N)_2_C_3_)^2+^	1.412 [160] ^2^		40 [160]	1.012 [160]	147.3 [160]	5.1 [160]	−19.30 [160]
35	((C_12_)_3_N)_2_C_6_)^2+^	1.445 [160] ^2^		42 [160]	1.131 [160]	146.8 [160]	6.9 [160]	−19.63 [160]
36	((C_8_C_1_C_1_N)_2_(OE)_3_Gly)^2+^	1.02 [161] ^2^	55.2 [161]		0.802 [161]			−47.5 [161]
37	((C_10_C_1_C_1_N)_2_(OE)_3_Gly)^2+^	0.859 [161]	32.55 [161]		1.466 [161]		3.863 [161]	−53.9 [161]
38	((C_12_C_1_C_1_N)_2_(OE)_3_Gly)^2+^	0.711 [161]	30.57 [161]		3 [161]		3.64 [161]	−37.1 [161]
39	((C_14_C_1_C_1_N)_2_(OE)_3_Gly)^2+^	0.243 [161]	34.5 [161]		0.947 [161]		4.727 [161]	−69.6 [161]
40	((C_16_C_1_C_1_N)_2_(OE)_3_Gly)^2+^	0.631 [161]	37 [161]		1.314 [161]		4.14 [161]	−55 [161]
41	((C_8_C_1_C_1_N)_2_(OE)_4_Gly)^2+^	1.822 [161]	49.08 [161]		0.519 [161]		3.417 [161]	−80.7 [161]
42	((C_10_C_1_C_1_N)_2_(OE)_4_Gly)^2+^	1.7 [161]	46.96 [161]		0.417 [161]		3.222 [161]	−85.3 [161]
43	((C_12_C_1_C_1_N)_2_(OE)_4_Gly)^2+^	1.239 [161]	28.54 [161]		1.06 [161]		4.124 [161]	−67.1 [161]
44	((C_14_C_1_C_1_N)_2_(OE)_4_Gly)^2+^	0.333 [161]	34.81 [161]		0.813 [161]		4.63 [161]	−75 [161]
45	((C_16_C_1_C_1_N)_2_(OE)_4_Gly)^2+^	0.389 [161]	36.51 [161]		0.785 [161]		4.71 [161]	−75.1 [161]
46	((C_8_C_1_C_1_N)_2_(OE)_5_Gly)^2+^	0.701 [161]	52.22 [161]		0.519 [161]			−83 [161]
47	((C_10_C_1_C_1_N)_2_(OE)_5_Gly)^2+^	0.649 [161]	48.28 [161]		0.827 [161]		3.539 [161]	−62.5 [161]
48	((C_12_C_1_C_1_N)_2_(OE)_5_Gly)^2+^	0.607 [161]	33.78 [161]		1.4 [161]		4 [161]	−55.1 [161]
49	((C_14_C_1_C_1_N)_2_(OE)_5_Gly)^2+^	0.502 [161]	33.61 [161]		1.04 [161]		4.389 [161]	−65.2 [161]
50	((C_16_C_1_C_1_N)_2_(OE)_5_Gly)^2+^	0.398 [161]	43.92 [161]		0.925 [161]		5.91 [161]	−60.0 [161]
51	(C_10_Pyrro)_2_C_4_)^2+^	3.3 [56]	43.2 [56]		1.37 [56]	121.2 [56]	2.96 [56]	
52	(C_12_Pyrro)_2_C_4_)^2+^	0.5 [162], 0.53 [56]	41.7 [56]		1.46 [56]	113.3 [56]	3.80 [56]	
53	(C_14_Pyrro)_2_C_4_)^2+^	0.1 [162], 0.108 [56]	40.4 [56]		1.59 [56]	104.4 [56]	4.41 [56]	
54	((C_8_Im)_3_Am)^3+^	4.3 [163]	33 [163]		1.11 [163]	1.50 [163]	3.13 [163]	
55	((C_8_Im)_3_Bn)^3+^	2.2 [163]	40 [163]		1.37 [163]	1.21 [163]	2.81 [163]	

^1^ Abbreviations: Imidazolium (Im), thioether-functionalized methylimidazolium (SMeIm), hydroxyl-functionalized imidazolium (OHIm), quaternary ammonium (C_x_C_y_C_z_N), ethylene oxide units (OE), glycol (Gly), pyrrolidinium (Pyrro), triethylamine (Am) and 1,3,5-trimethylbenzene (Bn). ^2^ Measurements done at 20 °C.

## Data Availability

Not applicable.

## Supplementary Materials

The following are available online at https://www.mdpi.com/article/10.3390/polym13071100/s1, Figure S1: Scheme of (A) micellar two-dimensional shape, (B) hemimicelle and (C) admicelle of monocationic and gemini ILBSs; Figure S2: Schematic representation of the formation of NP core (AgCl) in the poly(MMA-co-AM) shell; Figure S3:The molecular structure of 1-(2-acryloyloxyundecyl)-3-methylimidazolium bromide (ILBS-b); Figure S4: SEM of 4% ILBr undialyzed nanolatex coating on a glass slide before (a) and after (b) treatment with 0.1 mol L^−1^ KPF₆; (c) SEM image of film shaving fracture surface. Table S1: Literature data of ionic liquid-based surfactants aqueous solutions at 25 °C. Parameters calculated using techniques other than surface tension; Table S2: Literature data of gemini ionic liquid-based surfactants aqueous solutions at 25 °C. Parameters calculated using techniques other than surface tension.

## Abbreviations

A_min_	Minimum area per molecule at the water/air interface
AGET	Activators generated by electron transfer
AIBN	Azobisisobutyronitrile
AM	Acrylamide
ATRP	Atom transfer radical polymerizations
Aze⁺	Azepanium
Azo⁺	Azocanium
C₁, C₂, etc	Methyl, ethyl, etc, these alkyl groups have n-chain, unless indicated otherwise.
pC_20_	Surface tension reduction efficiency (by 20 mN m⁻^1^).
Cmc	Critical micelle concentration
CTAB	Cetyltrimethylammonium bromide
CTACl	Cetyltrimethylammonium chloride
DBS	Dodecylbenzene sulphonate
EO	Ethylene oxide unit
GILBS	Gemini ionic liquid-based surfactant, where two ILBSs are joined with a tether or a spacer.
Gu⁺	Guanidinium
HC	Surfactant hydrophobic group (or tail)
HG	Surfactant head-group
ILBS	Ionic liquid-based surfactant
Im^+^	Imidazolium ring
M_n_	Number average molar mass
M_w_	Weight average molar mass
MM	Molar mass
MMA	Methyl methacrylate monomer
MNP	Mesoporous nanoparticles
MSNP	Mesoporous silica nanoparticle
Mor⁺	Morpholinium
N⁺	Ammonium
Nagg	Average aggregation number of the micellar aggregate
NP	Nanoparticle
OA	Oleic acid
OHIm	Hydroxyl-functionalized imidazolium ring
O/W	Oil-in-water emulsion or microemulsion
P⁺	Phosphonium
Pip⁺	Piperidinium
PMMA	Polymethylmethacrylate (PMMA)
Pn^+^	2-Pyrrolidinonium
PS	Polystyrene
Py^+^	Pyridinium ring
Pyrro⁺	Pyrrolidinium
SDS	Sodium dodecyl sulphate, SDS
SMeIm	Thioether-functionalized methylimidazolium ring
TC	An AOT analogue, based on trans-aconitic acid
Triton X-100	Nonionic surfactant 2-[4-(2,4,4-trimethylpentan-2-yl)phenoxy]ethanol
Vn	Vinyl group, respectively
W/O	Water-in -oil emulsion or microemulsion
α_mic_	Degree of the surfactant counter-ion dissociation
Δ*G*^0^_ads_	Gibbs free energy of surfactant adsorption at the water/air interface
Δ*G*^0^_mic_	Gibbs free energy of micelle formation
Δ*H*^0^_mic_	Enthalpy of micelle formation
Δ*S*^0^_mic_	Entropy of micelle formation
γ_cmc_	Surface tension at cmc
Γ_cmc_	Surface pressure at cmc
Π_cmc_	Surface excess concentration at the interface
μE	Microemulsion

## References

[1] Pino V., Germán-Hernández M., Martín-Pérez A., Anderson J.L. (2012). Ionic Liquid-Based Surfactants in Separation Science. Sep. Sci. Technol..

[2] Cao H., Hu Y., Xu W., Wang Y., Guo X. (2020). Recent Progress in the Assembly Behavior of Imidazolium-Based Ionic Liquid Surfactants. J. Mol. Liq..

[3] Hejazifar M., Lanaridi O., Bica-Schröder K. (2020). Ionic Liquid Based Microemulsions: A Review. J. Mol. Liq..

[4] Freire M.G., Carvalho P.J., Fernandes A.M., Marrucho I.M., Queimada A.J., Coutinho J.A.P. (2007). Surface Tensions of Imidazolium Based Ionic Liquids: Anion, Cation, Temperature and Water Effect. J. Colloid Interface Sci..

[5] Lee D.J. (1995). Enthalpy-Entropy Compensation in Ionic Micelle Formation. Colloid Polym. Sci..

[6] Lunkenheimer K., Miller R. (1987). A Criterion for Judging the Purity of Adsorbed Surfactant Layers. J. Colloid Interface Sci..

[7] Lunkenheimer K., Pergande H.J., Krüger H. (1987). Apparatus for Programmed High-Performance Purification of Surfactant Solutions. Rev. Sci. Instrum..

[8] Freire M.G., Neves C.M.S.S., Marrucho I.M., Coutinho J.A.P., Fernandes A.M. (2010). Hydrolysis of Tetrafluoroborate and Hexafluorophosphate Counter Ions in Imidazolium-Based Ionic Liquids. J. Phys. Chem. A.

[9] Martins C.T., Sato B.M., El Seoud O.A. (2008). First Study on the Thermo-Solvatochromism in Aqueous 1-(1-Butyl)-3- Methylimidazolium Tetrafluoroborate: A Comparison between the Solvation by an Ionic Liquid and by Aqueous Alcohols. J. Phys. Chem. B.

[10] Xue Z. (2019). Hydrolysis of Ionic Liquids. Encyclopedia of Ionic Liquids.

[11] Swatloski R.P., Holbrey J.D., Rogers R.D. (2003). Ionic Liquids Are Not Always Green: Hydrolysis of 1-Butyl-3- Methylimidazolium Hexafluorophosphate. Green Chem..

[12] Shi H., Xie Y., Du C., Cong Y., Wang J., Zhao H. (2016). Thermodynamic Study of the Solubility of 2,4′-Dihydroxydiphenyl Sulfone in Nine Organic Solvents from T = (278.15 to 313.15) K and Thermodynamic Properties of Dissolution. J. Chem. Thermodyn..

[13] Wei Y., Wang F., Zhang Z., Ren C., Lin Y. (2014). Micellization and Thermodynamic Study of 1-Alkyl-3-Methylimidazolium Tetrafluoroborate Ionic Liquids in Aqueous Solution. J. Chem. Eng. Data.

[14] Lozano P., Bernal J.M., Vaultier M. (2011). Towards Continuous Sustainable Processes for Enzymatic Synthesis of Biodiesel in Hydrophobic Ionic Liquids/Supercritical Carbon Dioxide Biphasic Systems. Fuel.

[15] Zhang S., Gao Y., Dong B., Zheng L. (2010). Interaction between the Added Long-Chain Ionic Liquid 1-Dodecyl-3-Methylimidazolium Tetrafluoroborate and Triton X-100 in Aqueous Solutions. Colloids Surf. A Physicochem. Eng. Asp..

[16] Tejada-Casado C., Moreno-González D., García-Campaña A.M., del Olmo-Iruela M. (2015). Use of an Ionic Liquid-Based Surfactant as Pseudostationary Phase in the Analysis of Carbamates by Micellar Electrokinetic Chromatography. Electrophoresis.

[17] Malhotra S.V., Kumar V. (2010). A Profile of the in Vitro Anti-Tumor Activity of Imidazolium-Based Ionic Liquids. Bioorganic Med. Chem. Lett..

[18] Pan Q., Yang L., Meng X. (2013). Optimization of Enzymatic Synthesis of Tricaprylin in Ionic Liquids by Response Surface Methodology. JaocsJ. Am. Oil Chem. Soc..

[19] Bian M., Zhang Z., Yin H. (2012). Effects and Mechanism Characterization of Ionic Liquids as Mobile Phase Additives for the Separation of Matrine-Type Alkaloids by Liquid Chromatography. J. Pharm. Biomed. Anal..

[20] Lozano P., Gomez C., Nieto S., Sanchez-Gomez G., García-Verdugo E., Luis S.V. (2017). Highly Selective Biocatalytic Synthesis of Monoacylglycerides in Sponge-like Ionic Liquids. Green Chem..

[21] Nozaki Y., Yamaguchi K., Tomida K., Taniguchi N., Hara H., Takikawa Y., Sadakane K., Nakamura K., Konishi T., Fukao K. (2016). Phase Transition and Dynamics in Imidazolium-Based Ionic Liquid Crystals through a Metastable Highly Ordered Smectic Phase. J. Phys. Chem. B.

[22] Liu P., Zhou L., Yang C., Xia H., He Y., Feng M. (2015). A Complex Based on Imidazole Ionic Liquid and Copolymer of Acrylamide and Phenoxyacetamide Modification for Clay Stabilizer. J. Appl. Polym. Sci..

[23] Zha J.P., Zhu M.T., Qin L., Wang X.H. (2018). Study of Interaction between Ionic Liquids and Orange G in Aqueous Solution with UV-Vis Spectroscopy and Conductivity Meter. Spectrochim. Acta Part A Mol. Biomol. Spectrosc..

[24] Montaño D.F., Casanova H., Cardona W.I., Giraldo L.F. (2017). Functionalization of Montmorillonite with Ionic Liquids Based on 1-Alkyl-3-Methylimidazolium: Effect of Anion and Length Chain. Mater. Chem. Phys..

[25] Xiao S., Liu C., Chen L., Tan L., Chen Y. (2015). Liquid-Crystalline Ionic Liquids Modified Conductive Polymers as a Transparent Electrode for Indium-Free Polymer Solar Cells. J. Mater. Chem. A.

[26] Kato S., Freitag J., Gostomski P. (2013). Infinite Dilution Partial Molar Excess Entropy-Enthalpy Compensation for Thiophene, Carbon Dioxide and Water in Ionic Liquids. Fluid Phase Equilibria.

[27] Kuddushi M., Patel N.K., Rajput S., Shah A., El Seoud O.A., Malek N.I. (2018). Thermo-Switchable de Novo Ionic Liquid-Based Gelators with Dye-Absorbing and Drug-Encapsulating Characteristics. ACS Omega.

[28] Kuddushi M., Kumar A., Ray D., Aswal V.K., El Seoud O.A., Malek N.I. (2020). Concentration- And Temperature-Responsive Reversible Transition in Amide-Functionalized Surface-Active Ionic Liquids: Micelles to Vesicles to Organogel. ACS Omega.

[29] Wang G., Xu X., Sun Y., Zhuang L., Yao C. (2019). Relationship between Structure and Biodegradability of Gemini Imidazolium Surface Active Ionic Liquids. J. Mol. Liq..

[30] Ao M., Xu G., Kang W., Meng L., Gong H., Zhou T. (2011). Surface Rheological Behavior of Gelatin/Ionic Liquid-Type Imidazolium Gemini Surfactant Mixed Systems. Soft Matter.

[31] Zhuang L., Zheng C., Sun J., Yuan A., Wang G. (2014). Performances of Ramie Fiber Pretreated with Dicationic Imidazolium Ionic Liquid. Fibers Polym..

[32] Anderson J.L., Ding R., Ellern A., Armstrong D.W. (2005). Structure and Properties of High Stability Geminal Dicationic Ionic Liquids. J. Am. Chem. Soc..

[33] Khan A.S., Man Z., Arvina A., Bustam M.A., Nasrullah A., Ullah Z., Sarwono A., Muhammad N. (2017). Dicationic Imidazolium Based Ionic Liquids: Synthesis and Properties. J. Mol. Liq..

[34] Vélez J.F., Álvarez L.V., del Río C., Herradón B., Mann E., Morales E. (2017). Imidazolium-Based Mono and Dicationic Ionic Liquid Sodium Polymer Gel Electrolytes. Electrochim. Acta.

[35] Ehsani A., Kowsari E., Boorboor Ajdari F., Safari R., Mohammad Shiri H. (2017). Influence of Newly Synthesized Geminal Dicationic Ionic Liquid on Electrochemical and Pseudocapacitance Performance of Conductive Polymer Electroactive Film. J. Colloid Interface Sci..

[36] Flieger J., Flieger M. (2020). Ionic Liquids Toxicity—Benefits and Threats. Int. J. Mol. Sci..

[37] Stolte S., Steudte S., Igartua A., Stepnowski P. (2012). The Biodegradation of Ionic Liquids—The View from a Chemical Structure Perspective. Curr. Org. Chem..

[38] Costa S.P.F., Azevedo A.M.O., Pinto P.C.A.G., Saraiva M.L.M.F.S. (2017). Environmental Impact of Ionic Liquids: Recent Advances in (Eco)Toxicology and (Bio)Degradability. ChemSusChem.

[39] Welton T. (1999). Room-Temperature Ionic Liquids. Solvents for Synthesis and Catalysis. Chem. Rev..

[40] Picquet M., Tkatchenko I., Tommasi I., Wasserscheid P., Zimmermann J. (2003). Ionic Liquids, 3. Synthesis and Utilisation of Protic Imidazolium Salts in Homogeneous Catalysis. Adv. Synth. Catal..

[41] Thomaier S., Kunz W. (2007). Aggregates in Mixtures of Ionic Liquids. J. Mol. Liq..

[42] El Seoud O.A., Pires P.A.R., Abdel-Moghny T., Bastos E.L. (2007). Synthesis and Micellar Properties of Surface-Active Ionic Liquids: 1-Alkyl-3-Methylimidazolium Chlorides. J. Colloid Interface Sci..

[43] Ao M.Q., Xu G.Y., Zhu Y.Y., Bai Y. (2008). Synthesis and Properties of Ionic Liquid-Type Gemini Imidazolium Surfactants. J. Colloid Interface Sci..

[44] Inoue T., Ebina H., Dong B., Zheng L. (2007). Electrical Conductivity Study on Micelle Formation of Long-Chain Imidazolium Ionic Liquids in Aqueous Solution. J. Colloid Interface Sci..

[45] Baltazar Q.Q., Chandawalla J., Sawyer K., Anderson J.L. (2007). Interfacial and Micellar Properties of Imidazolium-Based Monocationic and Dicationic Ionic Liquids. Colloids Surf. A Physicochem. Eng. Asp..

[46] Kanjilal S., Sunitha S., Reddy P.S., Kumar K.P., Murty K.P.K., Prasad R.B.N. (2009). Synthesis and Evaluation of Micellar Properties and Antimicrobial Activities of Imidazole-Based Surfactants. Eur. J. Lipid Sci. Technol..

[47] Gordon C.M., Holbrey J.D., Kennedy A.R., Seddon K.R. (1998). Ionic Liquid Crystals: Hexafluorophosphate Salts. J. Mater. Chem..

[48] Aupoix A., Pégot B., Vo-Thanh G. (2010). Synthesis of Imidazolium and Pyridinium-Based Ionic Liquids and Application of 1-Alkyl-3-Methylimidazolium Salts as Pre-Catalysts for the Benzoin Condensation Using Solvent-Free and Microwave Activation. Tetrahedron.

[49] Cravotto G., Gaudino E.C., Boffa L., Lévêque J.M., Estager J., Bonrath W. (2008). Preparation of Second Generation Ionic Liquids by Efficient Solvent-Free Alkylation of N-Heterocycles with Chloroalkanes. Molecules.

[50] Deng Y., Morrissey S., Gathergood N., Delort A.M., Husson P., Gomes M.F.C. (2010). The Presence of Functional Groups Key for Biodegradation in Ionic Liquids: Effect on Gas Solubility. ChemSusChem.

[51] Baker G.A., Pandey S., Pandey S., Baker S.N. (2004). A New Class of Cationic Surfactants Inspired by N-Alkyl-N-Methyl Pyrrolidinium Ionic Liquids. Analyst.

[52] Lava K., Binnemans K., Cardinaels T. (2009). Piperidinium, Piperazinium and Morpholinium Ionic Liquid Crystals. J. Phys. Chem. B.

[53] Song Y., Li Q., Li Y. (2012). Self-Aggregation and Antimicrobial Activity of Alkylguanidium Salts. Colloids Surf. A: Physicochem. Eng. Asp..

[54] Zhuang L.H., Yu K.H., Wang G.W., Yao C. (2013). Synthesis and Properties of Novel Ester-Containing Gemini Imidazolium Surfactants. J. Colloid Interface Sci..

[55] Bhadani A., Singh S. (2011). Synthesis and Properties of Thioether Spacer Containing Gemini Imidazolium Surfactants. Langmuir.

[56] Zhang S., Yan H., Zhao M., Zheng L. (2012). Aggregation Behavior of Gemini Pyrrolidine-Based Ionic Liquids 1,1′-(Butane-1,4-Diyl)Bis(1-Alkylpyrrolidinium) Bromide ([C Npy-4-C Npy][Br 2]) in Aqueous Solution. J. Colloid Interface Sci..

[57] Goodchild I., Collier L., Millar S.L., Prokeš I., Lord J.C.D., Butts C.P., Bowers J., Webster J.R.P., Heenan R.K. (2007). Structural Studies of the Phase, Aggregation and Surface Behaviour of 1-Alkyl-3-Methylimidazolium Halide + Water Mixtures. J. Colloid Interface Sci..

[58] Saien J., Asadabadi S. (2013). Temperature Effect on Adsorption of Imidazolium-Based Ionic Liquids at Liquid-Liquid Interface. Colloids Surf. A Physicochem. Eng. Asp..

[59] Vaghela N.M., Sastry N.V., Aswal V.K. (2011). Surface Active and Aggregation Behavior of Methylimidazolium-Based Ionic Liquids of Type [Cnmim] [X], n = 4, 6, 8 and [X] = Cl-, Br-, and I- in Water. Colloid Polym. Sci..

[60] Tourné-Péteilh C., Devoisselle J.M., Vioux A., Judeinstein P., In M., Viau L. (2011). Surfactant Properties of Ionic Liquids Containing Short Alkyl Chain Imidazolium Cations and Ibuprofenate Anions. Phys. Chem. Chem. Phys..

[61] Bharmoria P., Singh T., Kumar A. (2013). Complexation of Chitosan with Surfactant like Ionic Liquids: Molecular Interactions and Preparation of Chitosan Nanoparticles. J. Colloid Interface Sci..

[62] Singh T., Boral S., Bohidar H.B., Kumar A. (2010). Interaction of Gelatin with Room-Temperature Ionic Liquids: A Detailed Physicochemical Study. J. Phys. Chem. B.

[63] Cornellas A., Perez L., Comelles F., Ribosa I., Manresa A., Garcia T.T. (2011). Self-Aggregation and Antimicrobial Activity of Imidazolium and Pyridinium Based Ionic Liquids in Aqueous Solution. J. Colloid Interface Sci..

[64] Aggarwal R., Singh S. (2018). Synthesis, Characterization and Evaluation of Surface and Thermal Properties of 3-Cyclohexyloxy-2-Hydroxypropyl Pyridinium and Imidazolium Surface-Active Ionic Liquids. J. Surfactants Deterg..

[65] Blesic M., Swadźba-Kwaśny M., Holbrey J.D., Canongia Lopes J.N., Seddon K.R., Rebelo L.P.N. (2009). New Catanionic Surfactants Based on 1-Alkyl-3-Methylimidazolium Alkylsulfonates, [CnH2n+1mim][CmH 2m+1SO3]: Mesomorphism and Aggregation. Phys. Chem. Chem. Phys..

[66] Sastry N.V., Vaghela N.M., Aswal V.K. (2012). Effect of Alkyl Chain Length and Head Group on Surface Active and Aggregation Behavior of Ionic Liquids in Water. Fluid Phase Equilibria.

[67] Ali A., Farooq U., Uzair S., Patel R. (2016). Conductometric and Tensiometric Studies on the Mixed Micellar Systems of Surface-Active Ionic Liquid and Cationic Surfactants in Aqueous Medium. J. Mol. Liq..

[68] Farooq U., Ali A., Patel R., Malik N.A. (2017). Self-Aggregation of Ionic Liquid-Cationic Surfactant Mixed Micelles in Water and in Diethylene Glycol–Water Mixtures: Conductometric, Tensiometric, and Spectroscopic Studies. J. Mol. Liq..

[69] Farooq U., Patel R., Ali A. (2018). Interaction of a Surface-Active Ionic Liquid with an Antidepressant Drug: Micellization and Spectroscopic Studies. J. Solut. Chem..

[70] Sharma R., Kamal A., Kang T.S., Mahajan R.K. (2015). Interactional Behavior of the Polyelectrolyte Poly Sodium 4-Styrene Sulphonate (NaPSS) with Imidazolium Based Surface Active Ionic Liquids in an Aqueous Medium. Phys. Chem. Chem. Phys..

[71] Naderi O., Sadeghi R. (2016). Effect of Temperature on the Aggregation Behaviour and Thermodynamic Properties of Surface Active Ionic Liquid 1-Decyl-3-Methylimidazolium Bromide in Aqueous Solutions: Surface Tension, Vapour Pressure Osmometery, Conductivity, Volumetric and Compressibil. J. Chem. Thermodyn..

[72] Qin L., Wang X.H. (2017). Surface Adsorption and Thermodynamic Properties of Mixed System of Ionic Liquid Surfactants with Cetyltrimethyl Ammonium Bromide. Rsc Adv..

[73] Zamani Z., Naderi O., Sadeghi R. (2018). Soluting-out Effect of Carbohydrates on the Surface Active Ionic Liquid 1-Decyl-3-Methylimidazolium Bromide in Aqueous Solutions. J. Chem. Thermodyn..

[74] Wen X., Yan Z., Kang Y., Zhang S. (2015). Apparent Molar Volume, Conductivity, and Fluorescence Studies of Ternary Systems of Dipeptides + Ionic Liquids ([Cnmim]Br, n = 10, 14) + Water at Different Temperatures. Colloid Polym. Sci..

[75] Ao M., Kim D. (2013). Aggregation Behavior of Aqueous Solutions of 1-Dodecyl-3-Methylimidazolium Salts with Different Halide Anions. J. Chem. Eng. Data.

[76] Pal A., Yadav S. (2020). Thermodynamic and Surface Properties of Aqueous 1-Dodecyl-3-Methylimidazolium Chloride [C12mim][Cl] Solution in the Presence of a Series of Inorganic Salts. J. Surfactants Deterg..

[77] Cognigni A., Gaertner P., Zirbs R., Peterlik H., Prochazka K., Schröder C., Bica K. (2016). Surface-Active Ionic Liquids in Micellar Catalysis: Impact of Anion Selection on Reaction Rates in Nucleophilic Substitutions. Phys. Chem. Chem. Phys..

[78] Singh G., Kaur M., Kang T.S., Aswal V.K. (2020). Aqueous Colloidal Systems of Bovine Serum Albumin and Functionalized Surface Active Ionic Liquids for Material Transport. RSC Adv..

[79] De Freitas D.V., Kuhn B.L., Bender C.R., Furuyama Lima A.M., de Freitas Lima M., Tiera M.J., Kloster C.L., Frizzo C.P., Villetti M.A. (2020). Thermodynamics of the Aggregation of Imidazolium Ionic Liquids with Sodium Alginate or Hydroxamic Alginate in Aqueous Solution. J. Mol. Liq..

[80] Liu X., Hu J., Huang Y., Fang Y. (2013). Aggregation Behavior of Surface Active Dialkylimidazolium Ionic Liquids [C12C n Im]Br (n = 1-4) in Aqueous Solutions. J. Surfactants Deterg..

[81] Gu Y., Shi L., Cheng X., Lu F., Zheng L. (2013). Aggregation Behavior of 1-Dodecyl-3-Methylimidazolium Bromide in Aqueous Solution: Effect of Ionic Liquids with Aromatic Anions. Langmuir.

[82] Nazemi T., Sadeghi R. (2014). Effect of Polar Organic Solvents on the Surface Adsorption and Micelle Formation of Surface Active Ionic Liquid 1-Dodecyl-3-Methylimidazolium Bromide in Aqueous Solutions and Comparison with the Traditional Cationic Surfactant Dodecyltrimethylammonium Bro. Colloids Surf. A Physicochem. Eng. Asp..

[83] Geng F., Liu J., Zheng L., Yu L., Li Z., Li G., Tung C. (2010). Micelle Formation of Long-Chain Imidazolium Ionic Liquids in Aqueous Solution Measured by Isothermal Titration Microcalorimetry. J. Chem. Eng. Data.

[84] Samarkina D.A., Gabdrakhmanov D.R., Lukashenko S.S., Khamatgalimov A.R., Zakharova L.Y. (2017). Aggregation Capacity and Complexation Properties of a System Based on an Imidazole-Containing Amphiphile and Bovine Serum Albumin. Russ. J. Gen. Chem..

[85] Blesic M., Lopes A., Melo E., Petrovski Z., Plechkova N.V., Canongia Lopes J.N., Seddon K.R., Rebelo L.P.N. (2008). On the Self-Aggregation and Fluorescence Quenching Aptitude of Surfactant Ionic Liquids. J. Phys. Chem. B.

[86] Pal A., Yadav A. (2018). Interactions Between Surface Active Ionic Liquid and Procaine Hydrochloride Drug in Aqueous Solution. J. Solut. Chem..

[87] Sharma R., Mahajan S., Mahajan R.K. (2013). Surface Adsorption and Mixed Micelle Formation of Surface Active Ionic Liquid in Cationic Surfactants: Conductivity, Surface Tension, Fluorescence and NMR Studies. Colloids Surf. A Physicochem. Eng. Asp..

[88] Sintra T.E., Vilas M., Martins M., Ventura S.P.M., Lobo Ferreira A.I.M.C., Santos L.M.N.B.F., Gonçalves F.J.M., Tojo E., Coutinho J.A.P. (2019). Synthesis and Characterization of Surface-Active Ionic Liquids Used in the Disruption of Escherichia Coli Cells. ChemPhysChem.

[89] Dong B., Zhao X., Zheng L., Zhang J., Li N., Inoue T. (2008). Aggregation Behavior of Long-Chain Imidazolium Ionic Liquids in Aqueous Solution: Micellization and Characterization of Micelle Microenvironment. Colloids Surf. A Physicochem. Eng. Asp..

[90] Das S., Ghosh S., Das B. (2018). Formation of Mixed Micelle in an Aqueous Mixture of a Surface Active Ionic Liquid and a Conventional Surfactant: Experiment and Modeling. J. Chem. Eng. Data.

[91] Keppeler N., Galgano P.D., Santos S.d.S., Malek N.I., El Seoud O.A. (2021). On the Effects of Head Group Volume on the Adsorption and Aggregation of 1-(n-Hexadecyl)-3-Cm-Imidazolium Bromide and Chloride Surfactants in Aqueous Solutions. J. Mol. Liq..

[92] Du M., Dai C., Chen A., Wu X., Li Y., Liu Y., Li W., Zhao M. (2015). Investigation on the Aggregation Behavior of Photo-Responsive System Composed of 1-Hexadecyl-3-Methylimidazolium Bromide and 2-Methoxycinnamic Acid. RSC Advances.

[93] Malek N.I., Vaid Z.S., More U.U., El Seoud O.A. (2015). Ionic-Liquid-Based Surfactants with Unsaturated Head Group: Synthesis and Micellar Properties of 1-(n-Alkyl)-3-Vinylimidazolium Bromides. Colloid Polym. Sci..

[94] Chabba S., Vashishat R., Kang T.S., Mahajan R.K. (2016). Self–Aggregation Behavior of Dialkyl Imidazolium Based Ionic Liquids in Aqueous Medium: Effect of Alkyl Chain Length. ChemistrySelect.

[95] Ge L., Wang Q., Wei D., Zhang X., Guo R. (2013). Aggregation of Double-Tailed Ionic Liquid 1,3-Dioctylimidazolium Bromide and the Interaction with Triblock Copolymer F127. J. Phys. Chem. B.

[96] Bou Malham I., Letellier P., Turmine M. (2008). Synthesis and Micellar Properties of 1-Decyl-2,3-Dimethylimidazolium Bromide Surfactant in Water and Water-Ethanolamine Mixtures at 298.15 K. J. Colloid Interface Sci..

[97] Pal A., Yadav A. (2018). Investigations of Drug Binding Ability of a Trisubstituted Surface Active Ionic Liquid 1-Dodecyl-2,3-Dimethylimidazolium Chloride [C12bmim][Cl]. J. Mol. Liq..

[98] Kaur R., Kumar S., Aswal V.K., Mahajan R.K. (2013). Influence of Headgroup on the Aggregation and Interactional Behavior of Twin-Tailed Cationic Surfactants with Pluronics. Langmuir.

[99] Sastry N.V., Vaghela N.M., Macwan P.M., Soni S.S., Aswal V.K., Gibaud A. (2012). Aggregation Behavior of Pyridinium Based Ionic Liquids in Water—Surface Tension, 1H NMR Chemical Shifts, SANS and SAXS Measurements. J. Colloid Interface Sci..

[100] Korotkikh O.P., Kochurova N.N. (2007). Temperature Effects on the Aggregation of Decylpyridinium Chloride in Aqueous Solution. Russ. J. Phys. Chem. A.

[101] Malovikova A., Hayakawa K., Kwak J.C.T. (1984). Surfactant-Polyelectrolyte Interactions. 4. Surfactant Chain Length Dependence of the Binding of Alkylpyridinium Cations to Dextran Sulfate. J. Phys. Chem..

[102] Korotkikh O.P., Kochurova N.N., Hong P. (2008). da Aggregation in the Aqueous Solutions of Alkylpyridinium Chlorides. Mendeleev Commun..

[103] Banjare R.K., Banjare M.K., Panda S. (2020). Effect of Acetonitrile on the Colloidal Behavior of Conventional Cationic Surfactants: A Combined Conductivity, Surface Tension, Fluorescence and FTIR Study. J. Solut. Chem..

[104] Fu D., Gao X., Huang B., Wang J., Sun Y., Zhang W., Kan K., Zhang X., Xie Y., Sui X. (2019). Micellization, Surface Activities and Thermodynamics Study of Pyridinium-Based Ionic Liquid Surfactants in Aqueous Solution. RSC Adv..

[105] Zhang H., Zhou X., Dong J., Zhang G., Wang C. (2007). A Novel Family of Green Ionic Liquids with Surface Activities. Sci. ChinaSer. B Chem..

[106] Singh D.K., Sastry N.V., Trivedi P.A. (2017). Amphiphilic Copolymers and Surface Active Ionic Liquid Systems in Aqueous Media—Surface Active and Aggregation Characteristics. Colloids Surf. A Physicochem. Eng. Asp..

[107] Tariq M., Podgoršek A., Ferguson J.L., Lopes A., Costa Gomes M.F., Pádua A.A.H., Rebelo L.P.N., Canongia Lopes J.N. (2011). Characteristics of Aggregation in Aqueous Solutions of Dialkylpyrrolidinium Bromides. J. Colloid Interface Sci..

[108] Shi L., Zhao M., Zheng L. (2011). Micelle Formation by N-Alkyl-N-Methylpyrrolidinium Bromide in Ethylammonium Nitrate. Colloids Surf. A Physicochem. Eng. Asp..

[109] Lukáč M., Pisárčik M., Lacko I., Devínsky F. (2010). Surface-Active Properties of Nitrogen Heterocyclic and Dialkylamino Derivates of Hexadecylphosphocholine and Cetyltrimethylammonium Bromide. J. Colloid Interface Sci..

[110] Sastry N.V., Singh D.K. (2016). Surfactant and Gelation Properties of Acetylsalicylate Based Room Temperature Ionic Liquid in Aqueous Media. Langmuir.

[111] Zhao Y., Yue X., Wang X., Huang D., Chen X. (2012). Micelle Formation by N-Alkyl-N-Methylpiperidinium Bromide Ionic Liquids in Aqueous Solution. Colloids Surf. A Physicochem. Eng. Asp..

[112] Mirgorodskaya A.B., Lukashenko S.S., Yatskevich E.I., Kulik N.V., Voloshina A.D., Kudryavtsev D.B., Panteleeva A.R., Zobov V.V., Zakharova L.Y., Konovalov A.I. (2014). Aggregation Behavior, Anticorrosion Effect, and Antimicrobial Activity of Alkylmethylmorpholinium Bromides. Prot. Met. Phys. Chem. Surf..

[113] Sharma R., Mahajan S., Mahajan R.K. (2014). Physicochemical Studies of Morpholinium Based Ionic Liquid Crystals and Their Interaction with Cyclodextrins. Fluid Phase Equilibria.

[114] Mirgorodskaya A.B., Valeeva F.G., Zakharov S.V., Kuryashov D.A., Bashkirtseva N.Y., Zakharova L.Y. (2018). Aggregation Behavior of Morpholinium Surfactants in the Presence of Organic Electrolytes. Russ. Chem. Bull..

[115] Miyake M., Oyama N. (2009). Effect of Amidoalkyl Group as Spacer on Aggregation Properties of Guanidine-Type Surfactants. J. Colloid Interface Sci..

[116] Gainanova G.A., Vagapova G.I., Syakaev V.V., Ibragimova A.R., Valeeva F.G., Tudriy E.V., Galkina I.V., Kataeva O.N., Zakharova L.Y., Latypov S.K. (2012). Self-Assembling Systems Based on Amphiphilic Alkyltriphenylphosphonium Bromides: Elucidation of the Role of Head Group. J. Colloid Interface Sci..

[117] Ibragimova A.R., Arkhipova D.M., Vagapova G.I., Ermolaev V.V., Galkina I.V., Nigmatullina L.S., Rizvanov I.K., Zakharova L.Y., Milyukov V.A., Konovalov A.I. (2014). Influence of the Medium Selforganization on the Catalytic Activity of Palladium Nanoparticles Stabilized by Amphiphilic Phosphonium Salts in the Suzuki Reaction. Russ. Chem. Bull..

[118] Thakkar K., Bharatiya B., Shah D.O., Ray D., Aswal V.K., Bahadur P. (2015). Interaction of Ionic Liquid Type Cationic Surfactants with Triton X-100 Nonionic Micelles. Colloids Surf. A Physicochem. Eng. Asp..

[119] Basu Ray G., Ghosh S., Moulik S.P. (2010). Ternary Mixtures of Alkyltriphenylphosphonium Bromides (C12TPB, C14TPB and C16TPB) in Aqueous Medium: Their Interfacial, Bulk and Fluorescence Quenching Behaviour. J. Chem. Sci..

[120] Lu F., Shi L., Yan H., Yang X., Zheng L. (2014). Aggregation Behavior of Dodecyltriphenylphosphonium Bromide in Aqueous Solution: Effect of Aromatic Ionic Liquids. Colloids Surf. A Physicochem. Eng. Asp..

[121] Verma S.K., Ghosh K.K. (2011). Micellar and Surface Properties of Some Monomeric Surfactants and a Gemini Cationic Surfactant. J. Surfactants Deterg..

[122] Mata J., Varade D., Bahadur P. (2005). Aggregation Behavior of Quaternary Salt Based Cationic Surfactants. Thermochim. Acta.

[123] Sehgal P., Kosaka O., Doe H. (2008). Interfacial and Aggregation Properties of the Binary Mixture of Decanoyl-N-Methyl-Glucamide and Hexadecyltriphenylphosphonium Bromide. Colloid Polym. Sci..

[124] Verma S.K., Ghosh K.K., Verma R., Xiang W., Li N., Zhao X. (2015). Surface, Conformational and Catalytic Activity Approach of α-Chymotrypsin and Trypsin in Micellar Media. Colloids Surf. A Physicochem. Eng. Asp..

[125] Gaynanova G.A., Valeeva F.G., Kushnazarova R.A., Bekmukhametova A.M., Zakharov S.V., Mirgorodskaya A.B., Zakharova L.Y. (2018). Effect of Hydrotropic Compounds on the Self-Organization and Solubilization Properties of Cationic Surfactants. Russ. J. Phys. Chem. A.

[126] Chakraborty A., Saha S.K., Chakraborty S. (2008). Effect of Size of Tetraalkylammonium Counterions on the Temperature Dependent Micellization of AOT in Aqueous Medium. Colloid Polym. Sci..

[127] Brown P., Butts C., Dyer R., Eastoe J., Grillo I., Guittard F., Rogers S., Heenan R. (2011). Anionic Surfactants and Surfactant Ionic Liquids with Quaternary Ammonium Counterions. Langmuir.

[128] Yu Z.J., Zhang X., Xu G., Zhao G.X. (1990). Physicochemical Properties of Aqueous Mixtures of Tetrabutylammonium Bromide and Anionic Surfactants. 3. Effects of Surfactant Chain Length and Salinity. J. Phys. Chem..

[129] Yu Z.J., Xu G. (1989). Physicochemical Properties of Aqueous Mixtures of Tetrabutylammonium Bromide and Anionic Surfactants. 1. Temperature-Induced Micellar Growth and Cloud Point Phenomenon. J. Phys. Chem..

[130] Rao K.S., Gehlot P.S., Gupta H., Drechsler M., Kumar A. (2015). Sodium Bromide Induced Micelle to Vesicle Transitions of Newly Synthesized Anionic Surface Active Ionic Liquids Based on Dodecylbenzenesulfonate. J. Phys. Chem. B.

[131] Jiao J., Dong B., Zhang H., Zhao Y., Wang X., Wang R., Yu L. (2012). Aggregation Behaviors of Dodecyl Sulfate-Based Anionic Surface Active Ionic Liquids in Water. J. Phys. Chem. B.

[132] Jin Y., Wang L., Wang T., Chen P., Bi Y., Yu L. (2015). Aggregation Behavior of Dodecylsulfonate-Based Surface Active Ionic Liquids in Water. J. Mol. Liq..

[133] Rao K.S., Trivedi T.J., Kumar A. (2012). Aqueous-Biamphiphilic Ionic Liquid Systems: Self-Assembly and Synthesis of Gold Nanocrystals/Microplates. J. Phys. Chem. B.

[134] Singh T., Rao K.S., Kumar A. (2012). Effect of Ethylene Glycol and Its Derivatives on the Aggregation Behavior of an Ionic Liquid 1-Butyl-3-Methyl Imidazolium Octylsulfate in Aqueous Medium. J. Phys. Chem. B.

[135] Comelles F., Ribosa I., González J.J., Garcia M.T. (2014). Micellization of Sodium Laurylethoxysulfate (SLES) and Short Chain Imidazolium Ionic Liquids in Aqueous Solution. J. Colloid Interface Sci..

[136] Pal A., Yadav A. (2015). Modulations in the Aggregation Behavior of Ionic Liquid 1-Butyl-3-Methylimidazolium Octylsulfate in Aqueous Alcohol Solutions. J. Mol. Liq..

[137] Thakkar K., Bharatiya B., Aswal V.K., Bahadur P. (2016). Aggregation of 1-Alkyl-3-Methylimidazolium Octylsulphate Ionic Liquids and Their Interaction with Triton X-100 Micelles. RSC Adv..

[138] Banjare M.K., Behera K., Satnami M.L., Pandey S., Ghosh K.K. (2017). Supra-Molecular Inclusion Complexation of Ionic Liquid 1-Butyl-3-Methylimidazolium Octylsulphate with Α- and Β-Cyclodextrins. Chem. Phys. Lett..

[139] Jiao J., Han B., Lin M., Cheng N., Yu L., Liu M. (2013). Salt-Free Catanionic Surface Active Ionic Liquids 1-Alkyl-3-Methylimidazolium Alkylsulfate: Aggregation Behavior in Aqueous Solution. J. Colloid Interface Sci..

[140] Brown P., Butts C.P., Eastoe J., Fermin D., Grillo I., Lee H.C., Parker D., Plana D., Richardson R.M. (2012). Anionic Surfactant Ionic Liquids with 1-Butyl-3-Methyl-Imidazolium Cations: Characterization and Application. Langmuir.

[141] Zhao Y., Du W., Sun L., Yu L., Jiao J., Wang R. (2013). Facile Synthesis of Calcium Carbonate with an Absolutely Pure Crystal Form Using 1-Butyl-3-Methylimidazolium Dodecyl Sulfate as the Modifier. Colloid Polym. Sci..

[142] Pal A., Yadav S. (2018). Effect of Cationic Polyelectrolyte Poly(Diallyldimethylammonium Chloride) on Micellization Behavior of Anionic Surface Active Ionic Liquid 1-Butyl-3-Methylimidazolium Dodecylsulfate [C4mim][C12SO4] in Aqueous Solutions. Colloid Polym. Sci..

[143] Pal A., Punia R. (2020). Self-Aggregation Behaviour of Cationic Surfactant Tetradecyltrimethylammonium Bromide and Bi-Amphiphilic Surface Active Ionic Liquid 3-Methyl-1-Pentylimidazolium Dodecylsulfate in Aqueous Solution. J. Mol. Liq..

[144] Pal A., Saini M. (2020). Effect of Alkyl Chain on Micellization Properties of Dodecylbenzenesulfonate Based Surface Active Ionic Liquids Using Conductance, Surface Tension, and Spectroscopic Techniques. J. Dispers. Sci. Technol..

[145] Silvas B.F.B., Marques E.F., Olsson U., Pons R. (2010). Headgroup Effects on the Unusual Lamellar-Lamellar Coexistence and Vesicle-to-Micelle Transition of Salt-Free Catanionic Amphiphiles. Langmuir.

[146] Galgano P.D., El Seoud O.A., Paul B.K., Moulik S.P. (2015). Ionic liquid-based surfactants: Synthesis, molecular structure, micellar properties and applications. Ionic Liquid-Based Surfactant Science: Formulation, Characterization, and Applications.

[147] Evans H.C. (1956). Alkyl Sulphates. Part I. Critical Micelle Concentration of the Sodium Salts. J. Chem. Soc..

[148] Shimizu S., Pires P.A.R., El Seoud O.A. (2004). Thermodynamics of Micellization of Benzyl (2-Acylaminoethyl) Dimethylammonium Chloride Surfactants in Aqueous Solutions: A Conductivity and Titration Calorimetry Study. Langmuir.

[149] Chatterjee A., Moulik S.P., Sanyal S.K., Mishra B.K., Puri P.M. (2001). Thermodynamics of Micelle Formation of Ionic Surfactants: A Critical Assessment for Sodium Dodecyl Sulfate, Cetyl Pyridinium Chloride and Dioctyl Sulfosuccinate (Na Salt) by Microcalorimetric, Conductometric, and Tensiometric Measurements. J. Phys. Chem. B.

[150] Schnee V.P., Palmer C.P. (2008). Cationic Surfactants for Micellar Electrokinetic Chromatography: 1. Characterization of Selectivity Using the Linear Solvation Energy Relationships Model. Electrophoresis.

[151] Engberts J.B.F.N., Nusselder J.J.H. (1990). The Effect of Chain Packing on Surfactant Aggregation in Aqueous Solution. Pure Appl. Chem..

[152] Benrraou M., Bales B.L., Zana R. (2003). Effect of the Nature of the Counterion on the Properties of Anionic Surfactants. 1. Cmc, Ionization Degree at the Cmc and Aggregation Number of Micelles of Sodium, Cesium, Tetramethylammonium, Tetraethylammonium, Tetrapropylammonium, and Tetrabutylammoniu. J. Phys. Chem. B.

[153] Guàrdia E., Skarmoutsos I., Masia M. (2009). On Ion and Molecular Polarization of Halides in Water. J. Chem. Theory Comput..

[154] Marcus Y. (1988). Ionic Radii in Aqueous Solutions. Chem. Rev..

[155] Bijma K., Engberts J.B.F.N. (1997). Effect of Counterions on Properties of Micelles Formed by Alkylpyridinium Surfactants. 1. Conductometry and 1H-NMR Chemical Shifts. Langmuir.

[156] Ao M., Huang P., Xu G., Yang X., Wang Y. (2009). Aggregation and Thermodynamic Properties of Ionic Liquid-Type Gemini Imidazolium Surfactants with Different Spacer Length. Colloid Polym. Sci..

[157] Pal A., Datta S., Aswal V.K., Bhattacharya S. (2012). Small-Angle Neutron-Scattering Studies of Mixed Micellar Structures Made of Dimeric Surfactants Having Imidazolium and Ammonium Headgroups. J. Phys. Chem. B.

[158] Maurya J.K., Khan A.B., Dohare N., Ali A., Kumar A., Patel R. (2018). Effect of Aromatic Amino Acids on the Surface Properties of 1-Dodecyl-3-(4-(3-Dodecylimidazolidin-1-Yl)Butyl)Imidazolidine Bromide Gemini Surfactant. J. Dispers. Sci. Technol..

[159] Kamboj R., Singh S., Bhadani A., Kataria H., Kaur G. (2012). Gemini Imidazolium Surfactants: Synthesis and Their Biophysiochemical Study. Langmuir.

[160] Tawfik S.M. (2015). Simple One Step Synthesis of Gemini Cationic Surfactant-Based Ionic Liquids: Physicochemical, Surface Properties and Biological Activity. J. Mol. Liq..

[161] Li H., Yu C., Chen R., Li J., Li J. (2012). Novel Ionic Liquid-Type Gemini Surfactants: Synthesis, Surface Property and Antimicrobial Activity. Colloids Surf. A Physicochem. Eng. Asp..

[162] Lu F., Zhang S., Zheng L. (2012). Dispersion of Multi-Walled Carbon Nanotubes (MWCNTs) by Ionic Liquid-Based Phosphonium Surfactants in Aqueous Solution. J. Mol. Liq..

[163] Nacham O., Martín-Pérez A., Steyer D.J., Trujillo-Rodríguez M.J., Anderson J.L., Pino V., Afonso A.M. (2015). Interfacial and Aggregation Behavior of Dicationic and Tricationic Ionic Liquid-Based Surfactants in Aqueous Solution. Colloids Surf. A Physicochem. Eng. Asp..

[164] In M., Zana R. (2007). Phase Behavior of Gemini Surfactants. J. Dispers. Sci. Technol..

[165] Welton T. (2004). Ionic Liquids in Catalysis. Coord. Chem. Rev..

[166] Plechkova N.V., Seddon K.R. (2008). Applications of Ionic Liquids in the Chemical Industry. Chem. Soc. Rev..

[167] Weingärtner H. (2008). Understanding Ionic Liquids at the Molecular Level: Facts, Problems, and Controversies. Angew. Chem. Int. Ed..

[168] Sawant A.D., Raut D.G., Darvatkar N.B., Salunkhe M.M. (2011). Recent Developments of Task-Specific Ionic Liquids in Organic Synthesis. Green Chem. Lett. Rev..

[169] Dupont J., Itoh T., Lozano P., Malhotra S.V. (2015). Environmentally Friendly Syntheses Using Ionic Liquids.

[170] Mohammad A.I. (2012). Green Solvents II: Properties and Applications of Ionic Liquids.

[171] Kuddushi M., Patel N.K., Gawali S.L., Mata J.P., Montes-Campos H., Varela L.M., Hassan P.A., Malek N.I. (2020). Thermo-Switchable de Novo Ionogel as Metal Absorbing and Curcumin Loaded Smart Bandage Material. J. Mol. Liq..

[172] Kuddushi M., Patel N.K., Rajput S., El Seoud O.A., Mata J.P., Malek N.I. (2020). Temperature-Responsive Low Molecular Weight Ionic Liquid Based Gelator: An Approach to Fabricate an Anti-Cancer Drug-Loaded Hybrid Ionogel. ChemSystemsChem.

[173] Rajput S.M., Mondal K., Kuddushi M., Jain M., Ray D., Aswal V.K., Malek N.I. (2020). Formation of Hydrotropic Drug/Gemini Surfactant Based Catanionic Vesicles as Efficient Nano Drug Delivery Vehicles. Colloids Interface Sci. Commun..

[174] Kulshrestha A., Gehlot P.S., Kumar A. (2020). Magnetic Proline-Based Ionic Liquid Surfactant as a Nano-Carrier for Hydrophobic Drug Delivery. J. Mater. Chem. B.

[175] Adawiyah N., Moniruzzaman M., Hawatulaila S., Goto M. (2016). Ionic Liquids as a Potential Tool for Drug Delivery Systems. MedChemComm.

[176] Shamshina J.L., Barber P.S., Rogers R.D. (2013). Ionic Liquids: Novel Applications in Drug Delivery. Expert Opin. Drug Deliv..

[177] Kuddushi M., Ray D., Aswal V., Hoskins C., Malek N. (2020). Poly(Vinyl Alcohol) and Functionalized Ionic Liquid-Based Smart Hydrogels for Doxorubicin Release. Acs Appl. Bio Mater..

[178] Zakrzewska M.E., Bogel-Łukasik E., Bogel-Łukasik R. (2011). Ionic Liquid-Mediated Formation of 5-Hydroxymethylfurfural-A Promising Biomass-Derived Building Block. Chem. Rev..

[179] Passos H., Freire M.G., Coutinho J.A.P. (2014). Ionic Liquids Solutions as Extractive Solvents of Value-Added Compounds from Biomass. Green Chem..

[180] Wang H., Gurau G., Rogers R.D. (2012). Ionic Liquid Processing of Cellulose. Chem. Soc. Rev..

[181] Pinkert A., Marsh K.N., Pang S., Staiger M.P. (2009). Ionic Liquids and Their Interaction with Cellulose. Chem. Rev..

[182] Amarasekara A.S. (2016). Acidic Ionic Liquids. Chem. Rev..

[183] Mai N.L., Kim C.K., Park B., Park H.J., Lee S.H., Koo Y.M. (2016). Prediction of Cellulose Dissolution in Ionic Liquids Using Molecular Descriptors Based QSAR Model. J. Mol. Liq..

[184] Yamauchi Y., Kuroda K. (2008). Rational Design of Mesoporous Metals and Related Nanomaterials by a Soft-Template Approach. Chem. Asian J..

[185] Tian T., Hu Q., Wang Y., Gao Y., Yu L. (2016). Effect of Imidazolium-Based Surface-Active Ionic Liquids on the Orientation of Liquid Crystals at Various Fluid/Liquid Crystal Interfaces. Langmuir.

[186] Goossens K., Lava K., Bielawski C.W., Binnemans K. (2016). Ionic Liquid Crystals: Versatile Materials. Chem. Rev..

[187] Kuddushi M., Mata J., Malek N. (2020). Self-Sustainable, Self-Healable, Load Bearable and Moldable Stimuli Responsive Ionogel for the Selective Removal of Anionic Dyes from Aqueous Medium. J. Mol. Liq..

[188] Shah A., Kuddushi M., Ray D., Aswal V.K., Malek N.I. (2020). Sodium Salicylate Mediated Ionic Liquid Based Catanionic Coacervates as Membrane-Free Microreactors for the Selective Sequestration of Dyes and Curcumin. ChemSystemsChem.

[189] Kuddushi M., Rajput S., Shah A., Mata J., Aswal V.K., El Seoud O.A., Kumar A., Malek N.I. (2019). Stimuli Responsive, Self-Sustainable, and Self-Healable Functionalized Hydrogel with Dual Gelation, Load-Bearing, and Dye-Absorbing Properties. ACS Appl. Mater. Interfaces.

[190] Shah A., Kuddushi M., Rajput S., El Seoud O.A., Malek N.I. (2018). Ionic Liquid-Based Catanionic Coacervates: Novel Microreactors for Membrane-Free Sequestration of Dyes and Curcumin. ACS Omega.

[191] Shah A., Jain M., Lad V.N., Ray D., Aswal V.K., Malek N.I. (2020). Selective Accumulation of Dyes and Curcumin in a Macroscopic Complex Coacervates Composed of Morpholinium Based Ester Functionalized Ionic Liquid and Sodium Salicylate. J. Mol. Liq..

[192] Qi L., Gong Y., Fang M., Jia Z., Cheng N., Yu L. (2020). Surface-Active Ionic-Liquid-Encapsulated Polyoxometalate Nanospheres: Construction, Self-Assembly, Adsorption Behavior, and Application for Dye Removal. ACS Appl. Nano Mater..

[193] Cheng N., Hu Q., Guo Y., Wang Y., Yu L. (2015). Efficient and Selective Removal of Dyes Using Imidazolium-Based Supramolecular Gels. ACS Appl. Mater. Interfaces.

[194] Jusoh R., Jalil A.A., Triwahyono S., Idris A., Haron S., Sapawe N., Jaafar N.F., Jusoh N.W.C. (2014). Synthesis of Reverse Micelle α-FeOOH Nanoparticles in Ionic Liquid as an Only Electrolyte: Inhibition of Electron-Hole Pair Recombination for Efficient Photoactivity. Appl. Catal. A Gen..

[195] Han P., Liu T., Ji X., Tang S. (2018). Morphology-Controlled Synthesis of Mesoporous Silica with Co-Template of Surfactant P123 and Ionic Liquid [Dmim]Cl. Chin. Chem. Lett..

[196] Brevet D., Jouannin C., Tourné-Péteilh C., Devoisselle J.M., Vioux A., Viau L. (2016). Self-Encapsulation of a Drug-Containing Ionic Liquid into Mesoporous Silica Monoliths or Nanoparticles by a Sol-Gel Process. RSC Adv..

[197] Isa E.D.M., Ahmad H., Rahman M.B.A. (2020). Long Chain Imidazolium Ionic Liquids as Templates in the Formation of Mesoporous Silica Nanospheres. Solid State Phenom..

[198] Mohamed Isa E.D., Abdul Rahman M.B., Ahmad H. (2018). Monodispersed Mesoporous Silica Nanospheres Based on Pyridinium Ionic Liquids. J. Porous Mater..

[199] Isa E.D.M., Ahmad H., Rahman M.B.A. (2019). Optimization of Synthesis Parameters of Mesoporous Silica Nanoparticles Based on Ionic Liquid by Experimental Design and Its Application as a Drug Delivery Agent. J. Nanomater..

[200] Zaharudin N.S., Mohamed Isa E.D., Ahmad H., Abdul Rahman M.B., Jumbri K. (2020). Functionalized Mesoporous Silica Nanoparticles Templated by Pyridinium Ionic Liquid for Hydrophilic and Hydrophobic Drug Release Application. J. Saudi Chem. Soc..

[201] Zhang M., Zhu W., Li H., Li M., Yin S., Li Y., Wei Y., Li H. (2016). Facile Fabrication of Molybdenum-Containing Ordered Mesoporous Silica Induced Deep Desulfurization in Fuel. Colloids Surf. A Physicochem. Eng. Asp..

[202] Zare A., Lashanizadegan A., Darvishi P., Zerafat M.M. (2020). Synthesis and Characterization of NaP Zeolite Nanocrystals Using [C12mim][Cl] Ionic Liquid. Chem. Pap..

[203] Hu J., Yang Q., Yang L., Zhang Z., Su B., Bao Z., Ren Q., Xing H., Dai S. (2015). Confining Noble Metal (Pd, Au, Pt) Nanoparticles in Surfactant Ionic Liquids: Active Non-Mercury Catalysts for Hydrochlorination of Acetylene. ACS Catal..

[204] Huang B., Huang C., Chen J., Sun X. (2017). Size-Controlled Synthesis and Morphology Evolution of Nd2O3 Nano-Powders Using Ionic Liquid Surfactant Templates. J. Alloy. Compd..

[205] Komal, Kaur H., Kainth M., Meena S.S., Kang T.S. (2019). Sustainable Preparation of Sunlight Active α-Fe2O3 Nanoparticles Using Iron Containing Ionic Liquids for Photocatalytic Applications. RSC Adv..

[206] Li M., Wang Y., Ye X., Wang Z., Wu T., Li C. (2017). Controlled Synthesis of Icosahedral Gold Nanocrystals, and Their Self-Assembly with an Ionic Liquid for Enhanced Immunosensing of Squamous Cell Carcinoma Antigen. Microchim. Acta.

[207] Xu Z.B., Lu G.P., Cai C. (2017). Palladium Nanoparticles Stabilized by Aqueous Vesicles Self-Assembled from a PEGylated Surfactant Ionic Liquid for the Chemoselective Reduction of Nitroarenes. Catal. Commun..

[208] Duan W., Li A., Chen Y., Zhuo K., Liu J., Wang J. (2018). Ionic Liquid-Assisted Synthesis of Reduced Graphene Oxide–Supported Hollow Spherical PtCu Alloy and Its Enhanced Electrocatalytic Activity toward Methanol Oxidation. J. Nanoparticle Res..

[209] Abbaszadegan A., Nabavizadeh M., Gholami A., Aleyasin Z.S., Dorostkar S., Saliminasab M., Ghasemi Y., Hemmateenejad B., Sharghi H. (2015). Positively Charged Imidazolium-Based Ionic Liquid-Protected Silver Nanoparticles: A Promising Disinfectant in Root Canal Treatment. Int. Endod. J..

[210] Lv X., Zhang L., Xing F., Lin H. (2016). Controlled Synthesis of Monodispersed Mesoporous Silica Nanoparticles: Particle Size Tuning and Formation Mechanism Investigation. Microporous Mesoporous Mater..

[211] Olivier-Bourbigou H., Magna L. (2002). Ionic Liquids: Perspectives for Organic and Catalytic Reactions. J. Mol. Catal. A Chem..

[212] Cole A.C., Jensen J.L., Ntai I., Tran K.L.T., Weaver K.J., Forbes D.C., Davis J.H. (2002). Novel Brønsted Acidic Ionic Liquids and Their Use as Dual Solvent-Catalysts. J. Am. Chem. Soc..

[213] Planellas M., Pleixats R., Shafir A. (2012). Palladium Nanoparticles in Suzuki Cross-Couplings: Tapping into the Potential of Tris-Imidazolium Salts for Nanoparticle Stabilization. Adv. Synth. Catal..

[214] MacLeod M.J., Johnson J.A. (2015). PEGylated N-Heterocyclic Carbene Anchors Designed to Stabilize Gold Nanoparticles in Biologically Relevant Media. J. Am. Chem. Soc..

[215] Yasukawa T., Miyamura H., Kobayashi S. (2015). Cellulose-Supported Chiral Rhodium Nanoparticles as Sustainable Heterogeneous Catalysts for Asymmetric Carbon-Carbon Bond-Forming Reactions. Chem. Sci..

[216] Visbal R., Gimeno M.C. (2014). N-Heterocyclic Carbene Metal Complexes: Photoluminescence and Applications. Chem. Soc. Rev..

[217] Naderi O., Nyman M., Amiri M., Sadeghi R. (2019). Synthesis and Characterization of Silver Nanoparticles in Aqueous Solutions of Surface Active Imidazolium-Based Ionic Liquids and Traditional Surfactants SDS and DTAB. J. Mol. Liq..

[218] Janiak C. (2013). Ionic Liquids for the Synthesis and Stabilization of Metal Nanoparticles. Z. Fur Nat. Sect. B J. Chem. Sci..

[219] Manojkumar K., Sivaramakrishna A., Vijayakrishna K. (2016). A Short Review on Stable Metal Nanoparticles Using Ionic Liquids, Supported Ionic Liquids, and Poly(Ionic Liquids). J. Nanoparticle Res..

[220] Tshemese Z., Masikane S.C., Mlowe S., Revaprasadu N., Rahman M.M. (2018). Progress in Green Solvents for the Stabilisation of Nanomaterials: Imidazolium Based Ionic Liquids. Recent Advances in Ionic Liquids.

[221] Verma M., Singh K., Bakshi M.S. (2019). Surface Active Magnetic Iron Oxide Nanoparticles for Extracting Metal Nanoparticles across an Aqueous-Organic Interface. J. Mater. Chem. C.

[222] Galgano P.D., El Seoud O.A. (2011). Surface Active Ionic Liquids: Study of the Micellar Properties of 1-(1-Alkyl)-3-Methylimidazolium Chlorides and Comparison with Structurally Related Surfactants. J. Colloid Interface Sci..

[223] Zhu W., Yang H., Yu Y., Hua L., Li H., Feng B., Hou Z. (2011). Amphiphilic Ionic Liquid Stabilizing Palladium Nanoparticles for Highly Efficient Catalytic Hydrogenation. Phys. Chem. Chem. Phys..

[224] Danielsson I., Lindman B. (1981). The Definition of Microemulsion. Colloids Surf..

[225] Moulik S.P., Paul B.K. (1998). Structure, Dynamics and Transport Properties of Micro Emulsions. Adv. Colloid Interface Sci..

[226] Winsor P.A. (1948). Hydrotropy, Solubilisation and Related Emulsification Processes. Part I. Trans. Faraday Soc..

[227] Yan F., Texter J. (2006). Surfactant Ionic Liquid-Based Microemulsions for Polymerization. Chem. Commun..

[228] Chen Z., Yan F., Qiu L., Lu J., Zhou Y., Chen J., Tang Y., Texter J. (2010). Sustainable Polymerizations in Recoverable Microemulsions. Langmuir.

[229] Wang G.X., Lu M., Liu L.C., Wu H., Zhong M. (2013). Fe-Mediated ARGET Atom Transfer Radical Polymerization of Methyl Methacrylate in Ionic Liquid-Based Microemulsion. J. Appl. Polym. Sci..

[230] Zhou Y., Qiu L., Deng Z., Texter J., Yan F. (2011). Low-Temperature AGET ATRP of Methyl Methacrylate in Ionic Liquid-Based Microemulsions. Macromolecules.

[231] Lu J., Ding Y., Yu Y., Wu S., Feng W. (2010). Effect of Two Kinds of Imidazolium Ionic Liquids on the Microemulsion Polymerization of Methyl Methylacrylate. Macromol. Symp..

[232] Mirhoseini F., Salabat A. (2015). Ionic Liquid Based Microemulsion Method for the Fabrication of Poly(Methyl Methacrylate)-TiO2 Nanocomposite as a Highly Efficient Visible Light Photocatalyst. RSC Adv..

[233] Wu L.G., Shen J.N., Du C.H., Wang T., Teng Y., van der Bruggen B. (2013). Development of AgCl/Poly(MMA-Co-AM) Hybrid Pervaporation Membranes Containing AgCl Nanoparticles through Synthesis of Ionic Liquid Microemulsions. Sep. Purif. Technol..

[234] Chakraborty S., Jähnichen K., Komber H., Basfar A.A., Voit B. (2014). Synthesis of Magnetic Polystyrene Nanoparticles Using Amphiphilic Ionic Liquid Stabilized RAFT Mediated Miniemulsion Polymerization. Macromolecules.

[235] England D., Tambe N., Texter J. (2012). Stimuli-Responsive Nanolatexes: Porating Films. ACS Macro Lett..

[236] Bonnenfold A., Ibarra M., Mecerreyes D., Leiza J.R. (2016). Adding Magnetic Ionic Liquid Monomers to the Emulsion PolymerizationTool-Box: Towards Polymer Latexes and Coatings with New Properties. J. Polym. Sci. A Polym. Chem..

[237] Fernandes A.M., Gracia R., Leal P.G., Paulis M., Mecerreyes D. (2014). Simple route to prepare stable liquid marbles using poly(ionic liquid)s. Polymer.

[238] Fernandes A.M., Mantione D., Gracia R., Leiza J.R., Paulis M., Mecerreyes D. (2015). From Polymer Latexes to Multifunctional Liquid Marbles. ACS Appl. Mater. Interfaces.

[239] Hanamertani A.S., Pilus R.M., Irawan S. (2017). A Review on the Application of Ionic Liquids for Enhanced Oil Recovery. Icipeg 2016.

[240] Nandwani S.K., Malek N.I., Chakraborty M., Gupta S. (2020). Insight into the Application of Surface-Active Ionic Liquids in Surfactant Based Enhanced Oil Recovery Processes-A Guide Leading to Research Advances. Energy Fuels.

[241] Pletnev I.V., Smirnova S.V., Shvedene N.V. (2019). New Directions in Using Ionic Liquids in Analytical Chemistry. 1: Liquid–Liquid Extraction. J. Anal. Chem..

[242] Pletnev I.V., Smirnova S.V., Shvedene N.V. (2019). New Directions in Using Ionic Liquids in Analytical Chemistry. 2: Electrochemical Methods. J. Anal. Chem..

[243] Nawała J., Dawidziuk B., Dziedzic D., Gordon D., Popiel S. (2018). Applications of Ionic Liquids in Analytical Chemistry with a Particular Emphasis on Their Use in Solid-Phase Microextraction. Trac Trends Anal. Chem..

[244] Trujillo-Rodríguez M.J., Nan H., Varona M., Emaus M.N., Souza I.D., Anderson J.L. (2019). Advances of Ionic Liquids in Analytical Chemistry. Anal. Chem..

