# Supplementary material for: Ionic Liquid-Based Surfactants: Recent Advances in Their Syntheses, Solution Properties, and Applications
Source: Polymers (Basel). 2021 Mar 30;13(7):1100. doi: 10.3390/polym13071100 (PMC8036849; doi:10.3390/polym13071100)
Supplement: Supplementary file 1 [file polymers-13-01100-s001.pdf]

polymers

Supplementary Material

Ionic Liquid-Based Surfactants: Recent advances in their syntheses, solution properties, and applications

Omar A. El Seoud [1,*], Nicolas Keppeler [1], Naved I. Malek [2] and Paula D. Galgano [1]

[1] Institute of Chemistry, the University of São Paulo, 05508-000 SP, Brazil.
[2] Applied Chemistry Department, Sardar Vallabhbhai National Institute of Technology, Surat, 395 007 Gujarat, India.
* Correspondence: elseoud.usp@gmail.com.

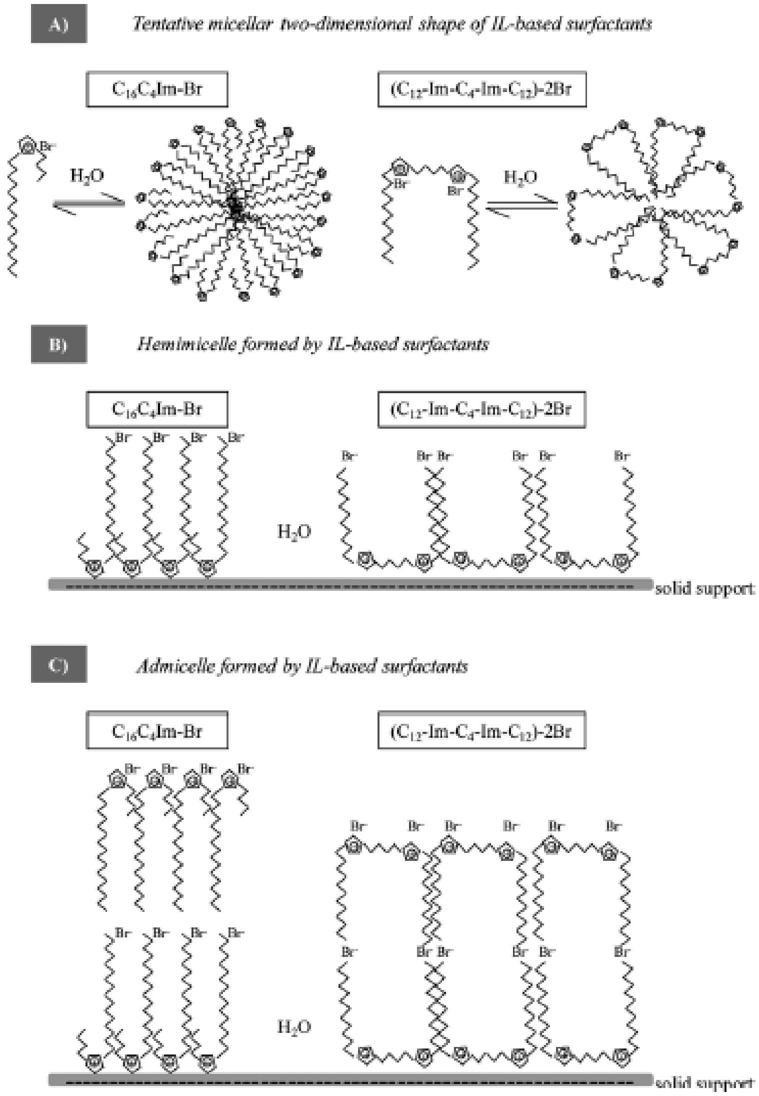

Figure S1. Scheme of A) micellar two-dimensional shape, B) hemimicelle, and C) admicelle of monocationic and gemini ILBSs [1].

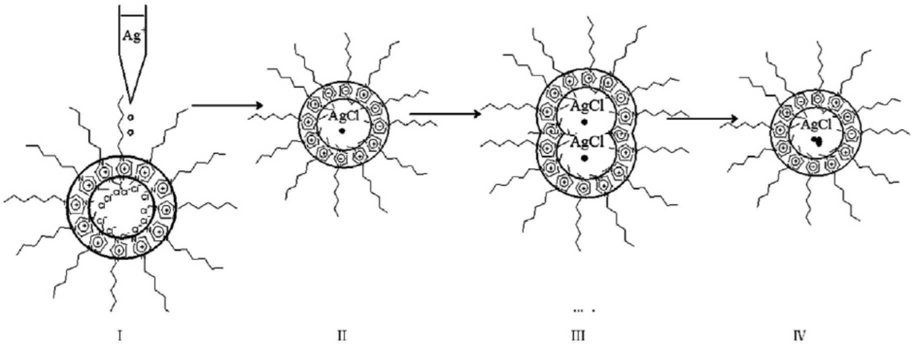

Figure S2. Schematic representation of the formation of NP core (AgCl) in the poly(MMA-co-AM) shell. Addition of aqueous $AgNO_3$ solution to the $C_{12}C_1$ImCl-based μE leads to formation of AgCl [2].

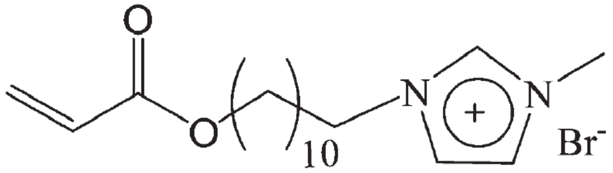

Figure S3. The molecular structure of 1-(2-acryloyloxyundecyl)-3-methylimidazolium bromide (ILBS-b) [3].

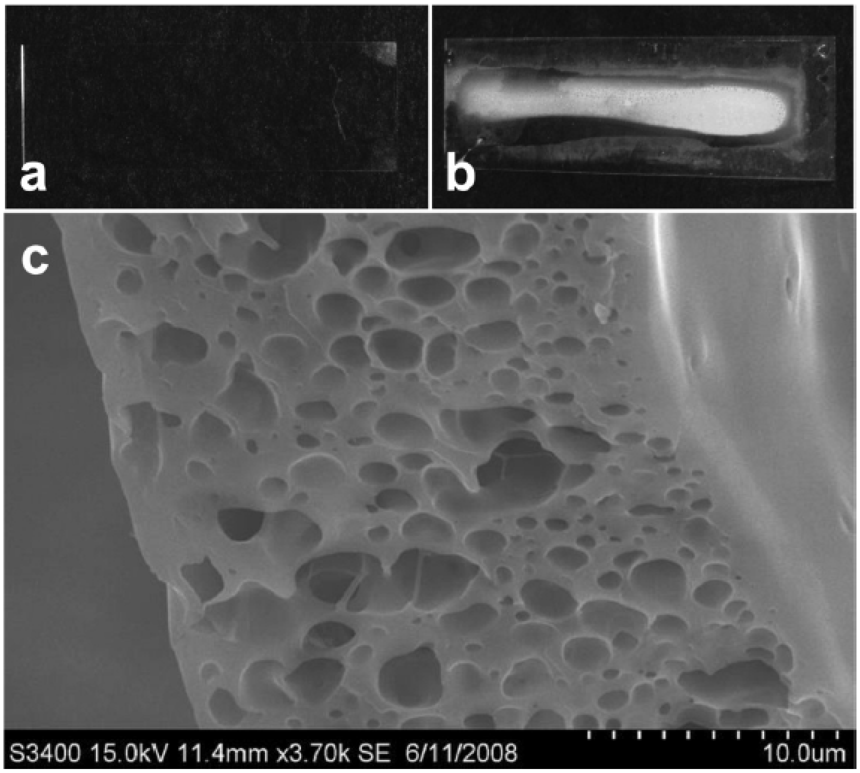

Figure S4. SEM of 4% ILBr undialyzed nanolatex coating on a glass slide before (a) and after (b) treatment with 0.1 mol L^{-1} KPF_6; (c) SEM image of film shaving fracture surface [4].

Table S1. Literature data of ionic liquid-based surfactants aqueous solutions at 25 °C. Parameters calculated using *techniques other than surface tension.*

Entry	Cation[1]	Anion[1]	cmc x 10^3 (mol L^{-1}) – Cond.[2]	cmc x 10^3 (mol L^{-1}) – Fluor.[3]	cmc x 10^3 (mol L^{-1}) – Other[4]	α_{mic} Frahm[5]	ΔG^0_{mic} (kJ mol^{-1})[6]	ΔH^0_{mic} (kJ mol^{-1}) – Cond.[7]	ΔH^0_{mic} (kJ mol^{-1}) – ITC[8]	N_{agg}[9]
**Cationic ILBSs**										
1	$C_8C_1Im^+$	Cl^-	101.2 [5],<br>205 [6],<br>107.8 [7],<br>174.2 [8],<br>150 [9],<br>210 [10],<br>179 [11]	184.8 [8],<br>210 [10],<br>234 [12]	90[10] [5],<br>100.8[11] [7],<br>240[12] [10]	0.410 [5],<br>0.65 [6],<br>0.56 [7],<br>0.34 [8],<br>0.664 [9],<br>0.37 [10]	-22.0 [5],<br>-18.7 [6],<br>-22.3 [7],<br>-20.9 [8],<br>-22.4 [10]	-1.56 [5]		39 [10],<br>23 [12]
2		Br^-	150.0 [5],<br>160 [10],<br>160 [13],<br>137.4 [14],<br>130 [15]	130 [13],<br>190 [10]	180[12] [10],<br>177[10] [13],<br>210[11] [15],<br>149[14] [16]	0.630 [5],<br>0.34 [10],<br>0.43 [13],<br>0.49 [14],<br>0.47 [15]	-24.8 [5],<br>-23.6 [10],<br>-20.7 [13],<br>-22.5 [14],<br>-23.0 [15]	-1.79 [5]		53 [10]
3		I^-	149.8 [5]			0.644 [5]	-26.0 [5]	-1.57 [5]		
5		$C_4SO_3^-$		155 [17]						
6	$C_9C_1Im^+$	Cl^-	130 [9],<br>76.1 [18]			0.528 [9],<br>0.51 [18]	-15.99 [18]	26.64 [18]		
7		Br^-	73.9 [18]			0.25 [18]	-18.66 [18]	0 [18]		
8	$C_{10}C_1Im^+$	Cl^-	78 [6],<br>59.9 [9],<br>48.7 [11],<br>57.3 [18],<br>40.47 [19],<br>53.81 [20],<br>58.80 [21],<br>58.0 [22],<br>58.5 [23],<br>33.5 [24],<br>54.6 [25]	53.8 [12],<br>40.52 [19],<br>57 [23],<br>33.4 [24]	39.0[14] [24],<br>41.3[15] [24]	0.54 [6],<br>0.572 [9],<br>0.52 [18],<br>0.51 [20],<br>0.57 [21],<br>0.53 [23],<br>0.52 [25]	-23.8 [6],<br>-14.84 [18],<br>-31.14 [19],<br>-25.61 [20],<br>-24.0 [21],<br>-23.12 [23],<br>-30.28 [23],<br>-26.12 [25]	6.22 [18],<br>5.00 [21],<br>-1.70 [25],<br>-1.70 [28]		32 [12],<br>40 [19],<br>22 [23]
9		Br^-	42 [13],<br>34.0 [15],<br>41.2 [18],<br>40.8 [26],<br>28.9 [27],<br>46.48 [28],<br>42.4 [29]	25 [13]	39[10] [13],<br>39.7[11] [15],<br>45[13] [16],<br>42.1[16] [26],<br>43.3[12] [26]	0.67 [13],<br>0.40 [15],<br>0.27 [18],<br>0.332 [26],<br>0.321 [27]	-29.7 [13],<br>-28.9 [15],<br>-15.94 [18],<br>-29.83 [26],<br>-37.44 [27],<br>-12.76 [28],<br>-10.4 [29]	-9.99 [26],<br>-8.28 [27],<br>-0.44 [28],<br>-1.78 [18]	-0.57 [15]	43 [28]

Entry	Cation[1]	Anion[1]	cmc x 10^3 (mol L^{-1}) – Cond.[2]	cmc x 10^3 (mol L^{-1}) – Fluor.[3]	cmc x 10^3 (mol L^{-1}) – Other[4]	α_{mic} Frahm[5]	ΔG^0_{mic} (kJ mol^{-1})[6]	ΔH^0_{mic} (kJ mol^{-1}) – Cond.[7]	ΔH^0_{mic} (kJ mol^{-1}) – ITC[8]	N_{agg}[9]
10		$C_1SO_3^-$		65 [17]						
11	$C_{11}C_1Im^+$	Cl^-	30.2 [9]			0.463 [9]				
12	$C_{12}C_1Im^+$	Cl^-	15.1 [9],<br>13.4 [11],<br>13.47 [19],<br>19.96 [20],<br>12.1 [24],<br>14.6 [30],<br>14.53 [31],<br>15.26 [32],<br>9.46 [33]	16.1 [12],<br>14.0 [19],<br>12.0 [24],<br>13.15 [32]	12.8^{17} [19],<br>15.0^{14} [24],<br>12.0^{15} [24],<br>9.09^{18} [33],<br>14.56^{11} [33]	0.461 [9],<br>0.44 [20],<br>0.44 [30],<br>0.43 [31],<br>0.352 [32]	-36.51 [19],<br>-30.65 [20],<br>-29.31 [30],<br>-29.1 [31],<br>-33.50 [32]	-2.62 [20],<br>-1.17 [30],<br>-7.16 [32]		51 [12],<br>58 [19],<br>40 [32]
13		Br^-	10 [13],<br>9.78 [15],<br>10.1 [27],<br>10.6 [30],<br>10.29 [31],<br>9.02 [29],<br>9.97 [34],<br>9.63 [35],<br>9.00 [36]	7.0 [13],<br>12.00 [36],<br>11.2 [37]	8.0^{10} [13],<br>9.10^{11} [15],<br>10^{13} [16],<br>10.24^{13} [31],<br>9.56^{11} [38],<br>10.79^{16} [39],<br>10.02^{16} [39],<br>10.11^{12} [39]	0.74 [13],<br>0.34 [15],<br>0.321 [27],<br>0.25 [30],<br>0.70 [31],<br>0.213 [34],<br>0.250 [35],<br>0.22 [36]	-37.2 [13],<br>-35.9 [15],<br>-43.17 [27],<br>-20.4 [29],<br>-37.12 [30],<br>-36.2 [31],<br>-38.21 [34],<br>-37.44 [35],<br>-37.2 [36]	-35.96 [27],<br>-4.88 [30]	-2.86 [15],<br>-3.51 [38]	37 [37],<br>81 [36]
14		I^-	5.2 [30],<br>5.19 [31]		4.12^{13} [31]	0.15 [30],<br>0.84 [31]	-42.51 [30],<br>-42.3 [31]	-16.60 [30]		
15		$C_1SO_3^-$		19 [17]						
16	$C_{13}C_1Im^+$	Cl^-	7.50 [9]			0.408 [9]				
17	$C_{14}C_1Im^+$	Cl^-	4.26 [9],<br>3.68 [19],<br>2.80 [24],<br>3.47 [40],<br>3.64 [41]	3.2 [12],<br>3.65 [19],<br>2.80 [24]	3.52^{17} [19],<br>3.30^{14} [24],<br>5.00^{15} [24],<br>3.69^{11} [41]	0.430 [9],<br>0.36 [40]	-42.91 [19],<br>-39.26 [40],<br>-24.0 [41]	-14.84 [40]		60 [12],<br>79 [19]
18		Br^-	2.5 [13],<br>2.68 [15],<br>2.47 [28],<br>2.46 [29],<br>2.50 [35],<br>2.80 [42],<br>2.4 [43],<br>2.34 [44]	1.8 [13],<br>2.9 [37]	1.4^{10} [13],<br>2.78^{11} [15],<br>2.52^{11} [38]	0.74 [13],<br>0.33 [15],<br>0.265 [35],<br>0.63 [42],<br>0.27 [43]	-43.1 [13],<br>-41.0 [15],<br>-25.92 [28],<br>-25.8 [29],<br>-42.90 [35],<br>-23.76 [42],<br>-43 [43]	-6.77 [28]	-4.01 [15],<br>-4.18 [38]	59 [28],<br>48 [37],<br>66 [42]
19	$C_{16}C_1Im^+$	Cl^-	1.4 [11],<br>0.86 [19],	1.30 [19]	0.92^{17} [19],<br>0.98^{11} [41]	0.55 [20],<br>0.45 [45]	-49.68 [19],<br>-38.45 [20],	-7.15 [20]		99 [19],<br>118^{20}

Entry	Cation[1]	Anion[1]	cmc x 10^3 (mol L^{-1}) – Cond.[2]	cmc x 10^3 (mol L^{-1}) – Fluor.[3]	cmc x 10^3 (mol L^{-1}) – Other[4]	α_{mic} Frahm[5]	ΔG^0_{mic} (kJ mol^{-1})[6]	ΔH^0_{mic} (kJ mol^{-1}) – Cond.[7]	ΔH^0_{mic} (kJ mol^{-1}) – ITC[8]	N_{agg}[9]
			1.25 [20], 1.05 [41], 0.882 [45], 0.91 [46]				-30.0 [41], -48.63 [46]			[19], 54 [45]
20		Br^-	0.59 [15], 0.611 [27], 0.56 [29], 0.65 [46]	0.84 [37]	0.61[11] [15], 0.64[11] [38]	0.33 [15], 0.316 [27], 0.283 [46]	-47.3 [15], -56.61 [27], -31.6 [29], -52.59 [46]	-33.36 [27], -4.43 [38]	-4.90 [15]	64 [37]
21	$C_{18}C_1Im^+$	Cl^-	0.37 [11], 0.44 [20]			0.601 [20]	-40.72 [20]	-7.50 [20]		
23	$C_{16}C_2Im^+$	Cl^-	0.58 [46]				-49.10 [46]			
24		Br^-	0.52 [46], 0.27 [47]	0.29 [47]	0.26[13] [47]	0.344 [46]	-53.09 [46], -50.70 [47]			
25	$C_{10}VnIm^+$	Br^-	33.22 [47]	24.13 [47]	26.86[13] [47]		-30.34 [47]	-4.87 [47]		
26	$C_{12}VnIm^+$	Br^-	8.17 [47]	11.33 [47]	8.75[13] [47]		-37.33 [47]	-8.83 [47]		
27		I^-	4.20 [48]		5.50[14] [48]					
28	$C_{14}VnIm^+$	Br^-	2.06 [47]	1.86 [47]	1.85[13] [47]		-43.59 [47]	-10.19 [47]		
29	$C_{16}VnIm^+$	Br^-	0.52 [46], 0.49 [47]	0.41 [47]	0.40[13] [47]	0.338 [46]	-53.01 [46], -50.02 [47]	-11.53 [47]		
31	$C_{16}C_3Im^+$	Cl^-	0.46 [46]				-49.37 [46]			
32		Br^-	0.41 [46]			0.440 [46]	-53.41 [46]			
33	$C_{16}AlIm^+$	Br^-	0.48 [46]			0.366 [46]	-52.98 [46]			
34	$C_8C_4Im^+$	Br^-	94.2 [49]	109.8 [49]	80.0[10] [49], 80.4[11] [49]		-23.2 [49]		18.3 [49]	53 [49], 60[10] [49]
36	$C_{12}C_4Im^+$	Br^-	5.1 [49]	4.2 [49]	5.5[10] [49], 5.0[11] [49]		-35.6 [49]		-2.5 [49]	46 [49], 54[10] [49]
37	$C_{16}C_4Im^+$	Cl^-	0.51 [46]				-49.52 [46]			
38		Br^-	0.30 [46]			0.498 [46]	-54.30 [46]			
39	$C_{16}C_5Im^+$	Cl^-	0.39 [46]				-49.90 [46]			
42	$C_{10}C_1C_1Im^+$	Br^-			45.2[12] [50]					
43	$C_{12}C_1C_1Im^+$	Cl^-	13.0 [51]	12.90 [51]			-35.84 [51]	-6.65 [51]		60 [51]
44	$C_{10}C_1C_{10}Im^+$	Cl^-	0.49 [52]	0.40 [52]	0.4[13] [52], 0.4[10] [52]		-27.01 [53]			
45	C_8Py^+	Cl^-	180 [54]		180[10] [54], 250[11] [55], 230[11] [56]	0.564 [54]	-20.4 [54]	-1.49 [54]	5.14 [55], 5.4 [56]	
46		Br^-	190 [13], 250 [57]		183[10] [13], 190[11] [55]	0.54 [13], 0.42 [57]	-20.5 [13]		1.32 [55]	

Entry	Cation[1]	Anion[1]	cmc x 10^3 (mol L^{-1}) – Cond.[2]	cmc x 10^3 (mol L^{-1}) – Fluor.[3]	cmc x 10^3 (mol L^{-1}) – Other[4]	α_{mic} Frahm[5]	ΔG^0_{mic} (kJ mol^{-1})[6]	ΔH^0_{mic} (kJ mol^{-1}) – Cond.[7]	ΔH^0_{mic} (kJ mol^{-1}) – ITC[8]	N_{agg}[9]
47		I^-			94[11] [58]				-5.5 [58]	
48	$C_{10}Py^+$	Cl^-	63.8 [59], 64.5 [60], 63.3 [61]		60[11] [55], 57[11] [56]	0.330 [60]	-26.2 [60]	-16.0 [60]	4.08 [55], 4.5 [56]	
49	$C_{10}Py^+$	Br^-	48 [13], 54 [57], 44 [62]		45[10] [13], 30[11] [55]	0.33 [13], 0.37 [57], 0.38 [62]	-29.2 [13]		0.31 [55]	
50	$C_{11}Py^+$	Br^-	21 [62]		18.6[11] [62]	0.36 [62]			2.3 [62]	
51	$C_{12}Py^+$	Cl^-	17.7 [60], 17.5 [61], 10 [62], 16.2 [63]		15[11] [55], 16[11] [56]	0.479 [60], 0.491 [63]	-31.2 [60]	-20.0 [60]	2.44 [55], 1.7 [56]	
52	$C_{12}Py^+$	Br^-	12 [13], 12.1 [57], 11.17 [63]	10 [13]	9.3[10] [13]	0.26 [13], 0.307 [57], 0.34 [62], 0.265 [63]	-36.4 [13]			
53	$C_{13}Py^+$	Br^-	5.3 [62]		4.6[11] [62]	0.34 [62]			5.4 [62]	
54	$C_{14}Py^+$	Cl^-	3.49 [60], 4.18 [63]			0.518 [60], 0.422 [63]	-37.5 [60]	-14.0 [60]		
55	$C_{14}Py^+$	Br^-	2.8 [13], 2.9 [57], 2.7 [62], 2.77 [63]	2.4 [13]	1.5[10] [13], 2.7[11] [62]	0.26 [13], 0.303 [57], 0.31 [62], 0.253 [63]	-42.9 [13]		6.9 [62]	
56	$C_{15}Py^+$	Br^-	1.3 [62], 1.36 [63]		1.3[11] [62]	0.31 [62], 0.275 [63]			8.3 [62]	
57	$C_{16}Py^+$	Cl^-	0.971 [63], 1.2 [64], 0.836 [65]	1.3 [64]		0.417 [63], 0.43 [64], 0.582 [65]	-6.03 [64], -43.25 [65]	-8.08 [65]		
58	$C_{16}Py^+$	Br^-	0.72 [44], 0.64 [62], 0.725 [63], 0.9 [64]	1.2 [64]		0.31 [62], 0.312 [63], 0.40 [64]	-44.41 [44], -6.0 [64]			
59	C_8-(*o*-C_1)Py^+	Cl^-	165 [54]			0.510 [54]	-21.5 [54]	-1.54 [54]		
60	C_{10}-(*o*-C_1)Py^+	Br^-	46.0 [66]				-35.2 [66]	0.92 [66]		
61	C_{12}-(*o*-C_1)Py^+	Br^-	10.2 [66]				-42.7 [66]	-3.4 [66]		
62	C_{14}-(*o*-C_1)Py^+	Br^-	2.6 [66]				-49.4 [66]	-6.3 [66]		
63	C_8-(*m*-C_1)Py^+	Cl^-	170 [54]		170[10] [54]	0.520 [54]	-21.2 [54]	-1.64 [54]		
64	C_8-(*m*-C_1)Py^+	Br^-	350 [67]							
65	C_{10}-(*m*-C_1)Py^+	Cl^-		44 [68]						

**Entry**	**Cation[1]**	**Anion[1]**	**cmc x 10^3 (mol L^{-1}) – Cond.[2]**	**cmc x 10^3 (mol L^{-1}) – Fluor.[3]**	**cmc x 10^3 (mol L^{-1}) – Other[4]**	**α_{mic} Frahm[5]**	**ΔG^0_{mic} (kJ mol^{-1})[6]**	**ΔH^0_{mic} (kJ mol^{-1}) – Cond.[7]**	**ΔH^0_{mic} (kJ mol^{-1}) – ITC[8]**	**N_{agg}[9]**
66	C_{12}-(*m*-C_1)Py$^+$	Cl$^-$		13.5 [68]	12.5[10] [68]					
67		Br$^-$	9.74 [69]		10.04[13] [69]		-37.12 [69]	-4.472 [69]		
68	C_{14}-(*m*-C_1)Py$^+$	Cl$^-$		3.1 [68]	3.2[10] [68]					
69		Br$^-$	2.40 [69]		2.33[13] [69]		-42.46 [69]	-6.237 [69]		
70	C_{16}-(*m*-C_1)Py$^+$	Cl$^-$		0.77 [68]	0.9[10] [68]					
71		Br$^-$	0.567 [69]		0.52[13] [69]		-48.50 [69]	-3.653 [69]		
72	C_{18}-(*m*-C_1)Py$^+$	Cl$^-$		0.23 [68]	0.25[10] [68]					
73	C_8-(*p*-C_1)Py$^+$	Cl$^-$	175 [54]		175[10] [54]	0.539 [54]	-20.8 [54]	1.59 [54]		
74		Br$^-$	160 [10]	130 [10]	170[12] [10]	0.32 [10]	-24.6 [10]			46 [10]
75	C_{12}-(*p*-C_1)Py$^+$	I$^-$	2.50 [70]							
76	C_{12}-(*p*-C_2)Py$^+$	I$^-$	2.21 [70]							
77	C_{12}-(*p*-C_3)Py$^+$	I$^-$	1.91 [70]							
78	C_{12}-(*p*-C_4)Py$^+$	I$^-$	1.54 [70]							
84	C_8C_1Pyrro$^+$	Br$^-$	200 [10]	200 [10]	240[12] [10]	0.42 [10]	-21.9 [10]			74 [10]
85	$C_{10}C_1$Pyrro$^+$	Br$^-$	55.5 [71]	60.1 [71], 56.7 [72], 56.3 [72]		0.322 [71]				
86	$C_{12}C_1$Pyrro$^+$	Cl$^-$		18.16 [73]						
87		Br$^-$	13.6 [71], 13.5 [74]	16.6 [71], 14.0 [72], 13.5 [72], 15 [75]	15.03[11] [15], 10[10] [75], 15.3[11] [75]	0.259 [71], 0.248 [74], 0.23 [75]	-35.6 [15], -35.9 [75], -36.12 [74]	-10.60 [74]	-1.28 [15], -1.78 [75]	49 [74]
88	$C_{14}C_1$Pyrro$^+$	Br$^-$	3.30 [71], 3.30 [74]	3.03 [71], 4.1 [72], 3.2 [72]	3.83[11] [15]	0.277 [71], 0.242 [74]	-41.8 [15], -42.38 [74]	-8.659 [74]	-2.54 [15]	55 [74]
89	$C_{16}C_1$Pyrro$^+$	Br$^-$	0.83 [71], 0.83 [72], 0.856 [74]	0.66 [71], 0.10 [72]	0.95[11] [15]	0.241 [71], 0.246 [74]	-47.6 [15], -48.15 [74]	-17.07 [74]	-2.80 [15]	59 [74], 69 [76]
90	$C_{18}C_1$Pyrro$^+$	Cl$^-$	0.42 [20]			0.528 [20]	-43.01 [20]	-8.16 [20]		
91		Br$^-$	0.25 [71]	0.22 [71], 0.33 [72]		0.466 [71]				
92	C_8C_4Pyrro$^+$	Br$^-$		150 [75]	120[10] [75], 144.7[11] [75]	0.51 [75]	-21.9 [75]		7.23 [75]	
93	$C_{12}C_4$Pyrro$^+$	Br$^-$			5[10] [75], 6.1[11] [75]	0.40 [75]	-36.1 [75]		2.57 [75]	
94	C_8Pip$^+$	Cl$^-$			280[12] [77]					
95	C_{12}Pip$^+$	Cl$^-$			14[12] [77]					
96	C_8C_1Pip$^+$	Cl$^-$			310[12] [77]					

Entry	Cation[1]	Anion[1]	cmc x 10^3 (mol L^{-1}) – Cond.[2]	cmc x 10^3 (mol L^{-1}) – Fluor.[3]	cmc x 10^3 (mol L^{-1}) – Other[4]	α_{mic} Frahm[5]	ΔG^0_{mic} (kJ mol^{-1})[6]	ΔH^0_{mic} (kJ mol^{-1}) – Cond.[7]	ΔH^0_{mic} (kJ mol^{-1}) – ITC[8]	N_{agg}[9]
97	$C_{10}C_1Pip^+$	Cl^-			70[12] [77]					
98	$C_{12}C_1Pip^+$	Cl^-	19.93 [78]	19.50 [78]	20[12] [77], 17.5[11] [78]	0.47 [78]	-30.08 [78]	-2.32 [78]	-0.97 [78]	66 [78], 86[10] [78]
99		Br^-					-35.84 [79]	-0.26 [79]		
100	$C_{14}C_1Pip^+$	Br^-					-42.03 [79]	-9.81 [79]		
101	$C_{16}C_1Pip^+$	Br^-	0.76 [76]				-47.47 [79]	-4.29 [79]		53 [76]
102	$C_{18}C_1Pip^+$	Cl^-				0.565 [20]	-41.69 [20]	-7.67 [20]		
103	$C_{16}C_1Aze^+$	Br^-	0.67 [76]							54 [76]
104	$C_{16}C_1Azo^+$ 35°C	Br^-	0.63 [76]							
106	$C_{12}C_1Mor^+$	Cl^-		22.66 [73]	25[11] [80]				-4.2 [80]	
108	$C_{14}C_1Mor^+$	Br^-	4.37 [81]		3.8[13] [82]	0.27 [81]	-15.5 [81]			61 [81]
109	$C_{16}C_1Mor^+$	Br^-	1.00 [81]		0.57[10] [83]	0.34 [81]	-9.6 [81], -19.3 [84]			58 [81]
111	C_8Gu^+	Cl^-	68 [85]	44.6 [86]		0.14 [85]	-33.8 [85]			
112	$C_{10}Gu^+$	Cl^-	20 [85], 26 [87]		2.84[11] [87]	0.06 [85], 0.29 [87]	-39.3 [85], -22.3 [87]		-4.5 [87]	
113	$C_{12}Gu^+$	Cl^-	5.8 [85], 6.2 [87], 6.0 [88]		6.2[11] [87]	0.04 [85], 0.26 [87]	-45.4 [85], -28.3 [87]		-7.8 [87]	
114	$C_{14}Gu^+$	Cl^-	1.8 [87]		1.5[11] [87], 0.13[13] [89]	0.28 [87]	-33.6 [87]		-17.5 [87]	
115	$C_8C_1C_1Gu^+$	Cl^-	7.6 [85]			0.34 [85]	-44.1 [85]			
116	$C_{10}C_1C_1Gu^+$	Cl^-	2.7 [85]			0.33 [85]	-49.2 [85]			
117	$C_{12}C_1C_1Gu^+$	Cl^-	0.72 [85]			0.18 [85]	-55.8 [85]			
118	$C_8Ph_3P^+$	Br^-	30 [90]		27[13] [90]					
119		I^-	22 [91]							
120	$C_{10}Ph_3P^+$	Br^-	5.6 [90], 7.21 [92], 7.54 [93]		5.6[14] [90], 2.3[10] [90], 6.2[13] [90], 7.26[11] [92], 5.3[13] [94]	0.867 [92], 0.52 [93]	-25.1 [92], -32.43 [93]	-4.47 [93]	3.97 [92]	74[19] [94]
121		I^-	19 [91]							
122	$C_{12}Ph_3P^+$	Br^-	1.7 [90], 1.82 [92], 1.81 [93], 1.75 [95], 2.20 [96]	1.8 [97]	1.6[10] [90], 2.1[13] [90], 1.80[11] [92], 1.15[13] [94], 1.2[13] [97]	0.604 [92], 0.56 [93], 0.64 [95], 0.40 [96]	-35.7 [92], -37.12 [93], -43.4 [95], -40.4 [96]	-6.19 [93]	1.15 [92]	90[19] [94]
123	$C_{14}Ph_3P^+$	Br^-	0.40 [90],	0.38 [97],	0.81[14] [90],	0.559 [92],	-39.9 [95],	-15.86 [93],	-5.96	107[19]

Entry	Cation^1	Anion^1	cmc x 10^3 (mol L^{-1}) – Cond.^{2}	cmc x 10^3 (mol L^{-1}) – Fluor.^{3}	cmc x 10^3 (mol L^{-1}) – Other^4	α_{mic} Frahm^5	ΔG^0_{mic} (kJ mol^{-1})^6	ΔH^0_{mic} (kJ mol^{-1}) – Cond.^{7}	ΔH^0_{mic} (kJ mol^{-1}) – ITC^8	N_{agg}^{9}
			0.77 [92], 0.55 [93], 0.57 [95], 0.83 [98], 0.69 [99]	0.71 [100], 0.71 [101]	0.41^{10} [90], 0.30^{13} [90], 0.35^{13} [94], 0.33^{13} [97], 0.59^{13} [98], 0.80^{11} [100]	0.61 [93], 0.62 [95], 0.499 [98], 0.54 [99]	-15.96 [98], -39.14 [93], -39.8 [100]	-20.13 [98]	[100]	[94]
124	$C_{16}Ph_3P^+$	Br^-	0.10 [90], 0.14 [92], 0.16 [93], 0.20 [95], 0.16 [96], 0.12 [102], 0.194 [104]	0.18 [97], 0.12 [100], 0.12 [101], 0.20 [104]	0.15^{14} [90], 0.062^{10} [90], 0.11^{13} [90], 0.10^{13} [94], 0.16^{13} [97], 0.115^{11} [100]	0.692 [92], 0.62 [93], 0.68 [95], 0.35 [96], 0.50 [102], 0.39 [104]	-46.24 [93], -41.7 [95], -52.5 [96], -42.4 [100], -36.5 [102], -31.8 [103], -50.1 [104]	-14.13 [93], -22.2 [104]	-19.90 [100]	159^{19} [94], 18 [104]
125	$C_{18}Ph_3P^+$	Br^-			0.02^{14} [90], 0.017^{10} [90]					
**Anionic ILBSs**										
126	$(CH_3)_4N^+$	$C_{12}SO_4^-$	5.4 [105]	5.9 [106], 5.1 [106]		0.20 [105]	-41.2 [105]			64 [105]
127		AOT^-	2.90 [107]				-30.8 [107]	-27.2 [107]		55 [94]
128	$(C_2H_5)_4N^+$	$C_{12}SO_4^-$	3.7 [105]			0.21 [105]	-42.6 [105]			63 [105]
129		AOT^-	2.50 [107]				-32.5 [107]	-12.3 [107]		78^{10} [94]
130	$(C_3H_7)_4N^+$	$C_{12}SO_4^-$	2.20 [105]			0.20 [105]	-45.2 [105]			57 [105]
131		AOT^-	0.95 [107]				-36.6 [107]	-8.8 [107]		131^{10} [94]
134	$(C_4H_9)_4N^+$	$C_{12}SO_4^-$	1.15 [105]			0.17 [105]	-48.9 [105]			57 [105]
135		$C_{14}SO_4^-$	0.21 [108]							
136		AOT^-	0.80 [107]				-34.4 [107]	15.2 [107]		139^{10} [94]
137	C_4Py^+	DBS^-			1.16^{11} [109]		-36.07 [109]		-3.51 [109]	
138	$C_4C_1Pyrro^+$	$C_{12}SO_4^-$	3.8 [110]			0.43 [110]	-37.27 [110]	-3.96 [110]		
139	$C_4C_1Im^+$	$C_8SO_3^-$		130 [17]						
140		$C_{12}SO_3^-$	4.70 [111]			0.39 [111]	-34.06 [111]	-0.71 [111]		
141		$C_8SO_4^-$	34.6 [7], 32.8 [8], 35.8 [112], 36.0 [113], 36.4 [114],	29.9 [8], 31.0 [113], 37.7 [114], 33 [119]	34.8^{11} [7], 37.0^{10} [117], 20^{13} [118], 40^{10} [120], 54.9^{11} [120]	0.30 [7], 0.64 [8], 0.29 [112], 0.39 [113], 0.45 [114],	-30.9 [7], -32.2 [8], -31.28 [112], -30.1 [113], -28.2 [114],	0.26 [112], -11.0 [113], -16.3 [114]		27^{20} [115], 19 [116], 27^{10} [117]

Entry	Cation[1]	Anion[1]	cmc x 10^3 (mol L^{-1}) – Cond.[2]	cmc x 10^3 (mol L^{-1}) – Fluor.[3]	cmc x 10^3 (mol L^{-1}) – Other[4]	α_{mic} Frahm[5]	ΔG^0_{mic} (kJ mol^{-1})[6]	ΔH^0_{mic} (kJ mol^{-1}) – Cond.[7]	ΔH^0_{mic} (kJ mol^{-1}) – ITC[8]	N_{agg}[9]
			32.4 [115], 30.5 [116], 36 [117], 30.0 [118]			0.31 [115], 0.63 [116], 0.63 [118]	-30.0 [117], -18.95 [118]			
142		$C_{10}SO_4^-$	8.4 [121]	11.0 [121]		0.48 [121]	-33.09 [121]	-5.69 [121]		
143		$C_{12}SO_4^-$	2.1 [110], 2.6 [112], 2.24 [122]	2.27 [122]		0.35 [110], 0.31 [112], 0.356 [122]	-41.71 [110], -41.66 [112], -41.23 [122]	-5.05 [110], -2.65 [112], -5.50 [122]		27 [122]
144		$C_{14}SO_4^-$	0.5 [121]	0.6 [121]		0.45 [121]	-44.61 [121]	-14.64 [121]		
145		DBS^-			1.47[11] [109]		-34.95 [109]		-2.79 [109]	
148	$C_5C_1Im^+$	$C_{12}SO_4^-$	1.6 [123]	1.6 [123]		0.399 [123]	-41.62 [123]	-13.94 [123]		134 [123]
149		DBS^-	0.36 [124]	0.33 [124]	0.35[13] [124]	0.687 [124]	-38.93 [124]	37.38 [124]		
150	$C_6C_1Im^+$	$C_8SO_4^-$	22.2 [112], 19.5 [115]			0.12 [112], 0.22 [115]	-36.49 [112]	0.59 [112]		77[20] [115]
151		$C_{12}SO_4^-$	0.9 [112], 0.9 [121]	1.3 [121]		0.18 [112], 0.35 [121]	-49.76 [112], -44.51 [121]	3.88 [112], -5.52 [121]		
152	$C_7C_1Im^+$	DBS^-	0.13 [124]	0.18 [124]	0.19[13] [124]	0.394 [124]	-51.54 [124]	6.75 [124]		
**Biamphiphilic ILBSs**										
153	$C_8C_1Im^+$	$C_8SO_3^-$		17 [17]						
154		$C_8SO_4^-$	5.6 [112]			0.06 [112]	-44.32 [112]	4.04 [112]		
155		$C_{12}SO_4^-$	0.4 [112], 0.3 [121]	0.4 [121]		0.16 [112], 0.32 [121]	-54.31 [112], -50.47 [121]	45.31 [112], -5.84 [121]		
156	$C_{10}C_1Im^+$	$C_{12}SO_4^-$	0.09 [121]			0.25 [121]	-57.80 [121]			
159	$C_8(CH_3)_3N^+$	$C_8SO_4^-$	7.64 [125]							
160		$C_{10}SO_4^-$	1.72 [125]							
161		$C_{12}SO_4^-$	0.46 [125]							

[1] Abbreviations and acronyms of the cations and anions are those given in Table 1. [2] Critical micelle concentration (cmc) from conductivity measurements. [3] Critical micelle concentration (cmc) from fluorescence measurements. [4] Critical micelle concentration (cmc) obtained by other. [5] Degree of counter-ion dissociation calculated with Frahm's approximation. [6] Gibbs free energy of micellization. [7] Enthalpy of micellization calculated indirectly by the van't Hoff treatment. [8] Enthalpy of micellization calculated directly by isothermal titration calorimetry. [9] Average micellar aggregation number from fluorescence measurements and other techniques. [10] Data determined by nuclear magnetic resonance spectroscopy (NMR). [11] Data determined by isothermal titration calorimetry (ITC). [12] Data determined by apparent molar volume. [13] Data determined by ultraviolet–visible spectroscopy (UV-Vis). [14] Data determined by potentiometry. [15] Data determined by differential pulse voltammetry (DPV). [16] Data determined by apparent molar adiabatic compressibility. [17] Data determined by electromagnetic field (EMF). [18] Data determined by turbidity. [19] Data determined by small-angle neutron scattering (SANS). [20] Data determined by light scattering.

Table S2. Literature data of gemini ionic liquid-based surfactants aqueous solutions at 25 °C. Parameters calculated using techniques other than surface tension. All surfactants have two bromides as counterions, except for entries 54 and 55, that have three bromide counterions.

Entry	Cation [1]	cmc x 10^3 (mol L^{-1}) – Cond.	cmc x 10^3 (mol L^{-1}) – Fluor.	α_{mic} Frahm	ΔG^0_{mic} (kJ mol^{-1})	ΔH^0_{mic} (kJ mol^{-1}) – Cond.	N_{agg}
2	$(C_{16}Im)_2C_2)^{2+}$		0.0341 [126] [2]				
3	$(C_{16}Im)_2C_3)^{2+}$		0.0048 [126] [2]				
4	$(C_{10}Im)_2C_4)^{2+}$	5.07 [127]		0.33 [127]	-26.96 [127], -28.7 [128]	-2.84 [127]	
5	$(C_{12}Im)_2C_4)^{2+}$	0.73 [127], 1.04 [129]		0.30 [127], 0.40 [129]	-33.41 [127], -43.09 [129], -33.7 [128]	-5.77 [127], -12.62 [129]	
6	$(C_{14}Im)_2C_4)^{2+}$	0.12 [127]		0.43 [127]	-34.58 [127], -36.52 [128]	-13.78 [127], -10.92 [128]	
7	$(C_{16}Im)_2C_4)^{2+}$		0.0222 [126] [2]				398 [126] [2]
8	$(C_{16}Im)_2C_5)^{2+}$		0.0269 [126] [2]				
10	$(C_{16}Im)_2C_6)^{2+}$		0.0501 [126] [2]				
12	$(C_{16}Im)_2C_8)^{2+}$		0.0512 [126] [2]				
15	$(C_{16}Im)_2C_{10})^{2+}$		0.0607 [126] [2]				
16	$(C_4Im)_2C_{12})^{2+}$	58.6 [130]	58.5 [130]				547 [130]
17	$(C_{10}Im)_2C_{12})^{2+}$	0.66 [130]	1.04 [130]				4.7 [130]
18	$(C_{16}Im)_2C_{12})^{2+}$	0.19 [130]	0.21 [130], 0.0619 [126] [2]				99.5 [130]
19	$((C_{12}SMeIm)_2C_2)^{2+}$	0.40 [131]		0.37 [131]	-33.14 [131]		
20	$((C_{14}SMeIm)_2C_2)^{2+}$	0.10 [131]		0.46 [131]	-34.08 [131]		
21	$((C_{16}SMeIm)_2C_2)^{2+}$	0.022 [131]		0.33 [131]	-42.72 [131]		
22	$((C_{12}SMeIm)_2C_3)^{2+}$	0.35 [131]		0.37 [131]	-33.52 [131]		
23	$((C_{14}SMeIm)_2C_3)^{2+}$	0.085 [131]		0.48 [131]	-33.83 [131]		
24	$((C_{16}SMeIm)_2C_3)^{2+}$	0.021 [131]		0.34 [131]	-42.49 [131]		
25	$((C_{12}SMeIm)_2C_4)^{2+}$	0.31 [131]		0.42 [131]	-32.36 [131]		
26	$((C_{14}SMeIm)_2C_4)^{2+}$	0.071 [131]		0.47 [131]	-34.62 [131]		
27	$((C_{16}SMeIm)_2C_4)^{2+}$	0.020 [131]		0.35 [131]	-42.26 [131]		
28	$((C_{12}OHIm)_2C_3)^{2+}$	1.37 [132]		0.25 [132]	-32.85 [132]		
29	$((C_{12}OHIm)_2C_4)^{2+}$	1.40 [132]		0.30 [132]	-31.63 [132]		
30	$((C_{12}OHIm)_2C_5)^{2+}$	1.47 [132]		0.16 [132]	-35.21 [132]		
31	$((C_{12}OHIm)_2C_6)^{2+}$	1.57 [132]		0.26 [132]	-32.31 [132]		
32	$((C_{12}OHIm)_2C_8)^{2+}$	1.59 [132]		0.30 [132]	-31.14 [132]		
33	$((C_{12})_3N)_2C_2)^{2+}$				-15.14 [133]		
34	$((C_{12})_3N)_2C_3)^{2+}$				-15.76 [133]		

35	$((C_{12})_3N)_2C_6)^{2+}$				-15.93 [133]		
51	$(C_{10}Pyrro)_2C_4)^{2+}$	4.7 [134]	4.5 [134]	0.36 [134]	-26.48 [134]	-16.60 [134]	
52	$(C_{12}Pyrro)_2C_4)^{2+}$	0.57 [134]	0.59 [134]	0.47 [134]	-29.31 [134]	-18.27 [134]	
53	$(C_{14}Pyrro)_2C_4)^{2+}$	0.106 [134]	0.078 [134]	0.37 [134]	-36.87 [134]	-24.02 [134]	
54	$((C_8Im)_3Am)^{3+}$	4.15 [130]	5.13 [130]				24.7 [130]
55	$((C_8Im)_3Bn)^{3+}$	1.87 [130]	2.30 [130]				98.6 [130]

[1] Abbreviations are those given in Table 2. [2] Measurements done at 30 °C.

References

1. Pino, V.; Germán-Hernández, M.; Martín-Pérez, A.; Anderson, J.L. Ionic Liquid-Based Surfactants in Separation Science. *Sep. Sci. Technol.* **2012**, *47*, 264–276, doi:10.1080/01496395.2011.620589.
2. Wu, L.G.; Shen, J.N.; Du, C.H.; Wang, T.; Teng, Y.; Bruggen, B. van der Development of AgCl/Poly(MMA-Co-AM) Hybrid Pervaporation Membranes Containing AgCl Nanoparticles through Synthesis of Ionic Liquid Microemulsions. *Sep. Purif. Technol.* **2013**, *114*, 117–125, doi:10.1016/j.seppur.2013.04.010.
3. Yan, F.; Texter, J. Surfactant Ionic Liquid-Based Microemulsions for Polymerization. *Chem. Commun.* **2006**, 2696–2698, doi:10.1039/b605287h.
4. England, D.; Tambe, N.; Texter, J. Stimuli-Responsive Nanolatexes: Porating Films. *ACS Macro Lett.* **2012**, *1*, 310–314, doi:10.1021/mz2002356.
5. Vaghela, N.M.; Sastry, N. v.; Aswal, V.K. Surface Active and Aggregation Behavior of Methylimidazolium-Based Ionic Liquids of Type [Cnmim] [X], n = 4, 6, 8 and [X] = Cl-, Br-, and I- in Water. *Colloid Polym. Sci.* **2011**, *289*, 309–322, doi:10.1007/s00396-010-2332-5.
6. Tourné-Péteilh, C.; Devoisselle, J.M.; Vioux, A.; Judeinstein, P.; In, M.; Viau, L. Surfactant Properties of Ionic Liquids Containing Short Alkyl Chain Imidazolium Cations and Ibuprofenate Anions. *Phys. Chem. Chem. Phys.* **2011**, *13*, 15523–15529, doi:10.1039/c1cp21057b.
7. Bharmoria, P.; Singh, T.; Kumar, A. Complexation of Chitosan with Surfactant like Ionic Liquids: Molecular Interactions and Preparation of Chitosan Nanoparticles. *J. Colloid Interface Sci.* **2013**, *407*, 361–369, doi:10.1016/j.jcis.2013.06.032.
8. Singh, T.; Boral, S.; Bohidar, H.B.; Kumar, A. Interaction of Gelatin with Room-Temperature Ionic Liquids: A Detailed Physicochemical Study. *J. Phys. Chem. B* **2010**, *114*, 8441–8448, doi:10.1021/jp102419f.
9. Belchior, D.C.V.; Sintra, T.E.; Carvalho, P.J.; Soromenho, M.R.C.; Esperança, J.M.S.S.; Ventura, S.P.M.; Rogers, R.D.; Coutinho, J.A.P.; Freire, M.G. Odd-Even Effect on the Formation of Aqueous Biphasic Systems Formed by 1-Alkyl-3-Methylimidazolium Chloride Ionic Liquids and Salts. *J. Chem. Phys.* **2018**, *148*, doi:10.1063/1.5012020.
10. Wang, H.; Wang, J.; Zhang, S.; Xuan, X. Structural Effects of Anions and Cations on the Aggregation Behavior of Ionic Liquids in Aqueous Solutions. *J. Phys. Chem. B* **2008**, *112*, 16682–16689, doi:10.1021/jp8069089.
11. Guzmán, N.M.; Fernández, J.F.; Parada, M.; Orbegozo, C.; Rodríguez, M.A.; Padrón, A.; Thöming, J. Efecto Del Catión, Del Anión y Del Co-Ión Sobre La Agregación de Líquidos Iónicos En Solución Acuosa. *Quim. Nova* **2010**, *33*, 1703–1708, doi:10.1590/s0100-40422010000800016.
12. Łuczak, J.; Jungnickel, C.; Markiewicz, M.; Hupka, J. Solubilization of Benzene, Toluene, and Xylene (BTX) in Aqueous Micellar Solutions of Amphiphilic Imidazolium Ionic Liquids. *J. Phys. Chem. B* **2013**, *117*, 5653–5658, doi:10.1021/jp3112205.
13. Cornellas, A.; Perez, L.; Comelles, F.; Ribosa, I.; Manresa, A.; Garcia, T.T. Self-Aggregation and Antimicrobial Activity of Imidazolium and Pyridinium Based Ionic Liquids in Aqueous Solution. *J. Colloid Interface Sci.* **2011**, *355*, 164–171, doi:10.1016/j.jcis.2010.11.063.
14. Aggarwal, R.; Singh, S. Synthesis, Characterization and Evaluation of Surface and Thermal Properties of 3-Cyclohexyloxy-2-Hydroxypropyl Pyridinium and Imidazolium Surface-Active Ionic Liquids. *J. Surfactants Deterg.* **2018**, *21*, 43–52, doi:10.1002/jsde.12002.
15. Liu, J.; Zhao, M.; Zhang, Q.; Sun, D.; Wei, X.; Zheng, L. Interaction between Two Homologues of Cationic Surface Active Ionic Liquids and the PEO-PPO-PEO Triblock Copolymers in Aqueous Solutions. *Colloid Polym. Sci.* **2011**, *289*, 1711–1718, doi:10.1007/s00396-011-2492-y.
16. Adam, C.G.; Bravo, M.V.; Granados, A.M. Anion Influence on Aggregation Behavior of Imidazolium-Based Ionic Liquid in Aqueous Solutions: Effect on Diverse Chemical Processes. *Ind. Eng. Chem. Res.* **2017**, *56*, 1214–1222, doi:10.1021/acs.iecr.6b03083.
17. Blesic, M.; Swadźba-Kwaśny, M.; Holbrey, J.D.; Canongia Lopes, J.N.; Seddon, K.R.; Rebelo, L.P.N. New Catanionic Surfactants Based on 1-Alkyl-3-Methylimidazolium Alkylsulfonates, [CnH2n+1mim][CmH 2m+1SO3]: Mesomorphism and Aggregation. *Phys. Chem. Chem. Phys.* **2009**, *11*, 4260–4268, doi:10.1039/b822341f.
18. El-Dossoki, F.I. Micellization Thermodynamics of Some Imidazolium Ionic Liquids in Aqueous Solutions—Conductometric Study. *J. Solut. Chem.* **2013**, *42*, 125–135, doi:10.1007/s10953-012-9947-8.
19. El Seoud, O.A.; Pires, P.A.R.; Abdel-Moghny, T.; Bastos, E.L. Synthesis and Micellar Properties of Surface-Active Ionic Liquids: 1-Alkyl-3-Methylimidazolium Chlorides. *J. Colloid Interface Sci.* **2007**, *313*, 296–304, doi:10.1016/j.jcis.2007.04.028.

20. Sastry, N. v.; Vaghela, N.M.; Aswal, V.K. Effect of Alkyl Chain Length and Head Group on Surface Active and Aggregation Behavior of Ionic Liquids in Water. *Fluid Phase Equilibria* **2012**, *327*, 22–29, doi:10.1016/j.fluid.2012.04.013.
21. Ali, A.; Farooq, U.; Uzair, S.; Patel, R. Conductometric and Tensiometric Studies on the Mixed Micellar Systems of Surface-Active Ionic Liquid and Cationic Surfactants in Aqueous Medium. *J. Mol. Liq.* **2016**, *223*, 589–602, doi:10.1016/j.molliq.2016.08.082.
22. Farooq, U.; Ali, A.; Patel, R.; Malik, N.A. Self-Aggregation of Ionic Liquid-Cationic Surfactant Mixed Micelles in Water and in Diethylene Glycol–Water Mixtures: Conductometric, Tensiometric, and Spectroscopic Studies. *J. Mol. Liq.* **2017**, *234*, 452–462, doi:10.1016/j.molliq.2017.03.109.
23. Farooq, U.; Patel, R.; Ali, A. Interaction of a Surface-Active Ionic Liquid with an Antidepressant Drug: Micellization and Spectroscopic Studies. *J. Solut. Chem.* **2018**, *47*, 568–585, doi:10.1007/s10953-018-0739-7.
24. Sharma, R.; Kamal, A.; Kang, T.S.; Mahajan, R.K. Interactional Behavior of the Polyelectrolyte Poly Sodium 4-Styrene Sulphonate (NaPSS) with Imidazolium Based Surface Active Ionic Liquids in an Aqueous Medium. *Phys. Chem. Chem. Phys.* **2015**, *17*, 23582–23594, doi:10.1039/c5cp02642c.
25. Mondal, S.; Ray, D.; Das, B. Thermodynamics of Aggregation of Imidazolium-Based Surface Active Ionic Liquids in Aqueous Poly(Ethylene Oxide) Media. *J. Chem. Thermodyn.* **2018**, *116*, 61–66, doi:10.1016/j.jct.2017.08.036.
26. Naderi, O.; Sadeghi, R. Effect of Temperature on the Aggregation Behaviour and Thermodynamic Properties of Surface Active Ionic Liquid 1-Decyl-3-Methylimidazolium Bromide in Aqueous Solutions: Surface Tension, Vapour Pressure Osmometery, Conductivity, Volumetric and Compressibil. *J. Chem. Thermodyn.* **2016**, *102*, 68–78, doi:10.1016/j.jct.2016.06.034.
27. Qin, L.; Wang, X.H. Surface Adsorption and Thermodynamic Properties of Mixed System of Ionic Liquid Surfactants with Cetyltrimethyl Ammonium Bromide. *Rsc Adv.* **2017**, *7*, 51426–51435, doi:10.1039/c7ra08915e.
28. Wen, X.; Yan, Z.; Kang, Y.; Zhang, S. Apparent Molar Volume, Conductivity, and Fluorescence Studies of Ternary Systems of Dipeptides + Ionic Liquids ([Cnmim]Br, n = 10, 14) + Water at Different Temperatures. *Colloid Polym. Sci.* **2015**, *293*, 2485–2495, doi:10.1007/s00396-015-3644-2.
29. de Freitas, D.V.; Kuhn, B.L.; Bender, C.R.; Furuyama Lima, A.M.; de Freitas Lima, M.; Tiera, M.J.; Kloster, C.L.; Frizzo, C.P.; Villetti, M.A. Thermodynamics of the Aggregation of Imidazolium Ionic Liquids with Sodium Alginate or Hydroxamic Alginate in Aqueous Solution. *J. Mol. Liq.* **2020**, *297*, 111734, doi:10.1016/j.molliq.2019.111734.
30. Ao, M.; Kim, D. Aggregation Behavior of Aqueous Solutions of 1-Dodecyl-3-Methylimidazolium Salts with Different Halide Anions. *J. Chem. Eng. Data* **2013**, *58*, 1529–1534, doi:10.1021/je301147k.
31. Cognigni, A.; Gaertner, P.; Zirbs, R.; Peterlik, H.; Prochazka, K.; Schröder, C.; Bica, K. Surface-Active Ionic Liquids in Micellar Catalysis: Impact of Anion Selection on Reaction Rates in Nucleophilic Substitutions. *Phys. Chem. Chem. Phys.* **2016**, *18*, 13375–13384, doi:10.1039/c6cp00493h.
32. Pal, A.; Yadav, S. Thermodynamic and Surface Properties of Aqueous 1-Dodecyl-3-Methylimidazolium Chloride [C12mim][Cl] Solution in the Presence of a Series of Inorganic Salts. *J. Surfactants Deterg.* **2020**, *23*, 53–65, doi:10.1002/jsde.12347.
33. Singh, G.; Kaur, M.; Kang, T.S.; Aswal, V.K. Aqueous Colloidal Systems of Bovine Serum Albumin and Functionalized Surface Active Ionic Liquids for Material Transport. *RSC Adv.* **2020**, *10*, 7073–7082, doi:10.1039/c9ra05549e.
34. Nazemi, T.; Sadeghi, R. Effect of Polar Organic Solvents on the Surface Adsorption and Micelle Formation of Surface Active Ionic Liquid 1-Dodecyl-3-Methylimidazolium Bromide in Aqueous Solutions and Comparison with the Traditional Cationic Surfactant Dodecyltrimethylammonium Bro. *Colloids Surf. A Physicochem. Eng. Asp.* **2014**, *462*, 271–279, doi:10.1016/j.colsurfa.2014.09.010.
35. Fan, X.; Zhao, K. Aggregation Behavior and Electrical Properties of Amphiphilic Pyrrole-Tailed Ionic Liquids in Water, from the Viewpoint of Dielectric Relaxation Spectroscopy. *Soft Matter* **2014**, *10*, 3259–3270, doi:10.1039/c3sm53143k.
36. Wang, J.; Zhang, L.; Wang, H.; Wu, C. Aggregation Behavior Modulation of 1-Dodecyl-3-Methylimidazolium Bromide by Organic Solvents in Aqueous Solution. *J. Phys. Chem. B* **2011**, *115*, 4955–4962, doi:10.1021/jp201604u.
37. Dong, B.; Zhao, X.; Zheng, L.; Zhang, J.; Li, N.; Inoue, T. Aggregation Behavior of Long-Chain Imidazolium Ionic Liquids in Aqueous Solution: Micellization and Characterization of Micelle Microenvironment. *Colloids Surf. A Physicochem. Eng. Asp.* **2008**, *317*, 666–672, doi:10.1016/j.colsurfa.2007.12.001.
38. Geng, F.; Liu, J.; Zheng, L.; Yu, L.; Li, Z.; Li, G.; Tung, C. Micelle Formation of Long-Chain Imidazolium Ionic Liquids in Aqueous Solution Measured by Isothermal Titration Microcalorimetry. *J. Chem. Eng. Data* **2010**, *55*, 147–151, doi:10.1021/je900290w.
39. Golabiazar, R.; Sadeghi, R. Salt-Effects in Aqueous Surface-Active Ionic Liquid 1-Dodecyl-3- Methylimidazolium Bromide Solutions: Volumetric and Compressibility Property Changes and Critical Aggregation Concentration Shifts. *J. Chem. Thermodyn.* **2014**, *76*, 29–44, doi:10.1016/j.jct.2014.03.001.
40. Pal, A.; Yadav, A. Interactions Between Surface Active Ionic Liquid and Procaine Hydrochloride Drug in Aqueous Solution. *J. Solut. Chem.* **2018**, *47*, 1096–1111, doi:10.1007/s10953-018-0778-0.
41. Usma, C.L.; Lindman, B.; Alfredsson, V.; Taboada, P.; Renamayor, C.S.; Pacios, I.E. Association of Imidazolium Surfactants with Poly(N-Isopropylacrylamide). *J. Mol. Liq.* **2018**, *265*, 46–52, doi:10.1016/j.molliq.2018.05.051.
42. Sharma, R.; Mahajan, S.; Mahajan, R.K. Surface Adsorption and Mixed Micelle Formation of Surface Active Ionic Liquid in Cationic Surfactants: Conductivity, Surface Tension, Fluorescence and NMR Studies. *Colloids Surf. A Physicochem. Eng. Asp.* **2013**, *427*, 62–75, doi:10.1016/j.colsurfa.2013.03.023.

43. Sintra, T.E.; Vilas, M.; Martins, M.; Ventura, S.P.M.; Lobo Ferreira, A.I.M.C.; Santos, L.M.N.B.F.; Gonçalves, F.J.M.; Tojo, E.; Coutinho, J.A.P. Synthesis and Characterization of Surface-Active Ionic Liquids Used in the Disruption of Escherichia Coli Cells. *ChemPhysChem* **2019**, *20*, 727–735, doi:10.1002/cphc.201801127.
44. He, Y.; Sun, L.; Fang, D.; Han, C.; Liu, C.; Luo, G. Aggregation Behaviour and Thermodynamics of Mixed Micellization of 1-Hexadecylpyridinium Bromide and Ionic Liquid in Ethylene Glycol/Water Binary Mixtures. *Colloid J.* **2014**, *76*, 96–103, doi:10.1134/S1061933X14010062.
45. Das, S.; Ghosh, S.; Das, B. Formation of Mixed Micelle in an Aqueous Mixture of a Surface Active Ionic Liquid and a Conventional Surfactant: Experiment and Modeling. *J. Chem. Eng. Data* **2018**, *63*, 3784–3800, doi:10.1021/acs.jced.8b00372.
46. Keppeler, N.; Galgano, P.D.; Santos, S. da S.; Malek, N.I.; El Seoud, O.A. On the Effects of Head Group Volume on the Adsorption and Aggregation of 1-(n-Hexadecyl)-3-Cm-Imidazolium Bromide and Chloride Surfactants in Aqueous Solutions. *J. Mol. Liq.* **2021**, *328*, 115478, doi:10.1016/j.molliq.2021.115478.
47. Malek, N.I.; Vaid, Z.S.; More, U.U.; El Seoud, O.A. Ionic-Liquid-Based Surfactants with Unsaturated Head Group: Synthesis and Micellar Properties of 1-(n-Alkyl)-3-Vinylimidazolium Bromides. *Colloid Polym. Sci.* **2015**, *293*, 3213–3224, doi:10.1007/s00396-015-3746-x.
48. Tribet, C.; Gaboriaud, R.; Lelievre, J. Micellar Polymerisation of Ionic Surfactants Studied by Conductimetry. *Polym. Int.* **1992**, *29*, 79–83, doi:10.1002/pi.4990290203.
49. Chabba, S.; Vashishat, R.; Kang, T.S.; Mahajan, R.K. Self–Aggregation Behavior of Dialkyl Imidazolium Based Ionic Liquids in Aqueous Medium: Effect of Alkyl Chain Length. *ChemistrySelect* **2016**, *1*, 2458–2470, doi:10.1002/slct.201600301.
50. Bou Malham, I.; Letellier, P.; Turmine, M. Synthesis and Micellar Properties of 1-Decyl-2,3-Dimethylimidazolium Bromide Surfactant in Water and Water-Ethanolamine Mixtures at 298.15 K. *J. Colloid Interface Sci.* **2008**, *328*, 166–171, doi:10.1016/j.jcis.2008.09.016.
51. Pal, A.; Yadav, A. Investigations of Drug Binding Ability of a Trisubstituted Surface Active Ionic Liquid 1-Dodecyl-2,3-Dimethylimidazolium Chloride [C12bmim][Cl]. *J. Mol. Liq.* **2018**, *251*, 167–177, doi:10.1016/j.molliq.2017.12.058.
52. Figueira-González, M.; Francisco, V.; García-Río, L.; Marques, E.F.; Parajó, M.; Rodríguez-Dafonte, P. Self-Aggregation Properties of Ionic Liquid 1,3-Didecyl-2-Methylimidazolium Chloride in Aqueous Solution: From Spheres to Cylinders to Bilayers. *J. Phys. Chem. B* **2013**, *117*, 2926–2937, doi:10.1021/jp3117962.
53. Kaur, R.; Kumar, S.; Aswal, V.K.; Mahajan, R.K. Influence of Headgroup on the Aggregation and Interactional Behavior of Twin-Tailed Cationic Surfactants with Pluronics. *Langmuir* **2013**, *29*, 11821–11833, doi:10.1021/la401864p.
54. Sastry, N. v.; Vaghela, N.M.; Macwan, P.M.; Soni, S.S.; Aswal, V.K.; Gibaud, A. Aggregation Behavior of Pyridinium Based Ionic Liquids in Water—Surface Tension, 1H NMR Chemical Shifts, SANS and SAXS Measurements. *J. Colloid Interface Sci.* **2012**, *371*, 52–61, doi:10.1016/j.jcis.2011.12.077.
55. Fisicaro, E.; Pelizzetti, E.; Barbieri, M. Aqueos Micellar Solutions of Some N-Alkylnicotinamide and N-Alkylpyridinium Halide Surfactants: Apparent and Partial Molar Enthalpies. *Thermochim. Acta* **1990**, *168*, 143–159.
56. Causi, S.; de Lisi, R.; Milioto, S. Thermodynamic Properties of N-Octyl- and N-Dodecylnicotinamide Chlorides in Water. *J. Solut. Chem.* **1991**, *20*, 1031–1058.
57. Jordan, D.; Tan, E.; Hegh, D. Synthesis, Characterization and Conductivity of Quaternary Nitrogen Surfactants Modified by the Addition of a Hydroxymethyl Substructure on the Head Group. *J. Surfactants Deterg.* **2012**, *15*, 587–592, doi:10.1007/s11743-012-1360-1.
58. Fisicaro, E.; Ghiozzi, A.; Pelizzetti, E.; Viscardi, G.; Quagliotto, P.L. Effect of Counterion on Thermodynamic Properties of Aqueous Micellar Solutions of 1-(3,3,4,4,5,5,6,6,6-Nonafluorohexyl) Pyridinium Halides. *J. Colloid Interface Sci.* **1996**, *184*, 147–154, doi:10.1006/jcis.1996.0605.
59. Korotkikh, O.P.; Kochurova, N.N. Temperature Effects on the Aggregation of Decylpyridinium Chloride in Aqueous Solution. *Russ. J. Phys. Chem. A* **2007**, *81*, 1059–1061, doi:10.1134/S0036024407070096.
60. Korotkikh, O.P.; Kochurova, N.N.; Hong, P. da Aggregation in the Aqueous Solutions of Alkylpyridinium Chlorides. *Mendeleev Commun.* **2008**, *18*, 347–349, doi:10.1016/j.mencom.2008.11.023.
61. Mehrian, T.; de Keizer, A.; Kortewegand, A.J.; Lyklema, J. No Title. *Colloids Surf. A Physicochem. Eng. Asp.* **1993**, *71*.
62. Škerjanc, J.; Kogej, K.; Cerar, J. Equilibrium and Transport Properties of Alkylpyridinium Bromides. *Langmuir* **1999**, *15*, 5023–5028, doi:10.1021/la981710+.
63. González-Pérez, A.; Varela, L.M.; García, M.; Rodríguez, J.R. Sphere to Rod Transitions in Homologous Alkylpyridinium Salts: A Stauff-Klevens-Type Equation for the Second Critical Micelle Concentration. *J. Colloid Interface Sci.* **2006**, *293*, 213–221, doi:10.1016/j.jcis.2005.06.026.
64. Banjare, R.K.; Banjare, M.K.; Panda, S. Effect of Acetonitrile on the Colloidal Behavior of Conventional Cationic Surfactants: A Combined Conductivity, Surface Tension, Fluorescence and FTIR Study. *J. Solut. Chem.* **2020**, *49*, 34–51, doi:10.1007/s10953-019-00937-4.
65. Mahjoub, H.F.; Aouissi, Z.; Othman, T. Influence of Carboxymethylcellulose (NaCMC) on the Aggregation and Micellization Behaviors in Aqueous Cethylpyridinium Chloride Solutions: Thermodynamic Study and Effect of Polymer Concentration. *J. Mol. Liq.* **2018**, *265*, 473–486, doi:10.1016/j.molliq.2018.06.037.
66. Adderson, J.E.; Taylor, H. The Effects of Temperature on the Critical Micelle Concentrations of Alkyl A-picolinium Bromides. *J. Pharm. Pharmacol.* **1970**, *22*, 523–530, doi:10.1111/j.2042-7158.1970.tb10557.x.

67. Fayyaz, S.; Talat, R.; Ali, S.; Khalid, N.; Shah, A.; Ullah, F. Synthesis, Characterization, and Micellization Behavior of Cationic Surfactants: N-Alkyl-3-Methylpyridinium Bromides and Their Drug Interaction Study by UV–Visible Spectroscopy and Conductometry. *J. Surfactants Deterg.* **2019**, *22*, 625–632, doi:10.1002/jsde.12263.
68. Blesic, M.; Lopes, A.; Melo, E.; Petrovski, Z.; Plechkova, N. v.; Canongia Lopes, J.N.; Seddon, K.R.; Rebelo, L.P.N. On the Self-Aggregation and Fluorescence Quenching Aptitude of Surfactant Ionic Liquids. *J. Phys. Chem. B* **2008**, *112*, 8645–8650, doi:10.1021/jp802179j.
69. Fu, D.; Gao, X.; Huang, B.; Wang, J.; Sun, Y.; Zhang, W.; Kan, K.; Zhang, X.; Xie, Y.; Sui, X. Micellization, Surface Activities and Thermodynamics Study of Pyridinium-Based Ionic Liquid Surfactants in Aqueous Solution. *RSC Adv.* **2019**, *9*, 28799–28807, doi:10.1039/c9ra04226a.
70. Engberts, J.B.F.N.; Nusselder, J.J.H. The Effect of Chain Packing on Surfactant Aggregation in Aqueous Solution. *Pure Appl. Chem.* **1990**, *62*, 47–55, doi:10.1351/pac199062010047.
71. Baker, G.A.; Pandey, S.; Pandey, S.; Baker, S.N. A New Class of Cationic Surfactants Inspired by N-Alkyl-N-Methyl Pyrrolidinium Ionic Liquids. *Analyst* **2004**, *129*, 890–892, doi:10.1039/b410301g.
72. Karukstis, K.K.; McDonough, J.R. Characterization of the Aggregates of N-Alkyl-N-Methylpyrrolidinium Bromide Surfactants in Aqueous Solution. *Langmuir* **2005**, *21*, 5716–5721, doi:10.1021/la047015l.
73. Singh, D.K.; Sastry, N. v.; Trivedi, P.A. Amphiphilic Copolymers and Surface Active Ionic Liquid Systems in Aqueous Media—Surface Active and Aggregation Characteristics. *Colloids Surf. A Physicochem. Eng. Asp.* **2017**, *524*, 111–126, doi:10.1016/j.colsurfa.2017.04.033.
74. Shi, L.; Zhao, M.; Zheng, L. Micelle Formation by N-Alkyl-N-Methylpyrrolidinium Bromide in Ethylammonium Nitrate. *Colloids Surf. A Physicochem. Eng. Asp.* **2011**, *392*, 305–312, doi:10.1016/j.colsurfa.2011.09.064.
75. Tariq, M.; Podgoršek, A.; Ferguson, J.L.; Lopes, A.; Costa Gomes, M.F.; Pádua, A.A.H.; Rebelo, L.P.N.; Canongia Lopes, J.N. Characteristics of Aggregation in Aqueous Solutions of Dialkylpyrrolidinium Bromides. *J. Colloid Interface Sci.* **2011**, *360*, 606–616, doi:10.1016/j.jcis.2011.04.083.
76. Schnee, V.P.; Palmer, C.P. Cationic Surfactants for Micellar Electrokinetic Chromatography: 1. Characterization of Selectivity Using the Linear Solvation Energy Relationships Model. *Electrophoresis* **2008**, *29*, 767–776, doi:10.1002/elps.200700494.
77. Milioto, S.; Causi, S.; de Lisi, R. Thermodynamic Properties of Some N-Alkyl-N-Methylpiperidinium Chlorides and N-Alkylpiperidine Hydrochlorides in Water. *J. Solut. Chem.* **1993**, *22*, 1–26, doi:10.1007/BF00647052.
78. Sastry, N. v.; Singh, D.K. Surfactant and Gelation Properties of Acetylsalicylate Based Room Temperature Ionic Liquid in Aqueous Media. *Langmuir* **2016**, *32*, 10000–10016, doi:10.1021/acs.langmuir.6b02074.
79. Zhao, Y.; Yue, X.; Wang, X.; Huang, D.; Chen, X. Micelle Formation by N-Alkyl-N-Methylpiperidinium Bromide Ionic Liquids in Aqueous Solution. *Colloids Surf. A Physicochem. Eng. Asp.* **2012**, *412*, 90–95, doi:10.1016/j.colsurfa.2012.07.021.
80. Rózycka-Roszak, B.; Fisicaro, E. Thermochemical Study of Aqueous Micellar Solutions of Some Amphiphilic Quaternary Ammonium Chlorides at 313 K. *Thermochim. Acta* **1992**, *205*, 19–31, doi:10.1016/0040-6031(92)85245-Q.
81. Sharma, R.; Mahajan, S.; Mahajan, R.K. Physicochemical Studies of Morpholinium Based Ionic Liquid Crystals and Their Interaction with Cyclodextrins. *Fluid Phase Equilibria* **2014**, *361*, 104–115, doi:10.1016/j.fluid.2013.10.042.
82. Mirgorodskaya, A.B.; Ya Zakharova, L.; Khairutdinova, E.I.; Lukashenko, S.S.; Sinyashin, O.G. Supramolecular Systems Based on Gemini Surfactants for Enhancing Solubility of Spectral Probes and Drugs in Aqueous Solution. *Colloids Surf. A: Physicochem. Eng. Asp.* **2016**, *510*, 33–42, doi:10.1016/j.colsurfa.2016.07.065.
83. Zakharova, L.Y.; Pashirova, T.N.; Kashapov, R.R.; Zhil'Tsova, E.P.; Gaisin, N.K.; Gnezdilov, O.I.; Konov, A.B.; Lukashenko, S.S.; Magdeev, I.M. Catalytic Properties of Micellar Systems Based on 4-Aza-1-Alkyl-1- Azoniabicyclo[2.2.2]Octane Bromides. *Kinet. Catal.* **2011**, *52*, 179–185, doi:10.1134/S0023158411020236.
84. Mirgorodskaya, A.B.; Valeeva, F.G.; Zakharov, S. v.; Kuryashov, D.A.; Bashkirtseva, N.Y.; Zakharova, L.Y. Aggregation Behavior of Morpholinium Surfactants in the Presence of Organic Electrolytes. *Russ. Chem. Bull.* **2018**, *67*, 291–296, doi:10.1007/s11172-018-2072-0.
85. Song, Y.; Li, Q.; Li, Y. Self-Aggregation and Antimicrobial Activity of Alkylguanidium Salts. *Colloids Surf. A: Physicochem. Eng. Asp.* **2012**, *393*, 11–16, doi:10.1016/j.colsurfa.2011.10.015.
86. Pacheco-Fernández, I.; Pino, V.; Ayala, J.H.; Afonso, A.M. Guanidinium Ionic Liquid-Based Surfactants as Low Cytotoxic Extractants: Analytical Performance in an in-Situ Dispersive Liquid–Liquid Microextraction Method for Determining Personal Care Products. *J. Chromatogr. A* **2018**, *1559*, 102–111, doi:10.1016/j.chroma.2017.04.061.
87. Bouchal, R.; Hamel, A.; Hesemann, P.; In, M.; Prelot, B.; Zajac, J. Micellization Behavior of Long-Chain Substituted Alkylguanidinium Surfactants. *Int. J. Mol. Sci.* **2016**, *17*, doi:10.3390/ijms17020223.
88. Miyake, M.; Oyama, N. Effect of Amidoalkyl Group as Spacer on Aggregation Properties of Guanidine-Type Surfactants. *J. Colloid Interface Sci.* **2009**, *330*, 180–185, doi:10.1016/j.jcis.2008.10.047.
89. Onda, M.; Yoshihara, K.; Koyano, H.; Ariga, K.; Kunitake, T. Molecular Recognition of Nucleotides by the Guanidinium Unit at the Surface of Aqueous Micelles and Bilayers. A Comparison of Microscopic and Macroscopic Interfaces. *J. Am. Chem. Soc.* **1996**, *118*, 8524–8530, doi:10.1021/ja960991+.
90. Gainanova, G.A.; Vagapova, G.I.; Syakaev, V. v.; Ibragimova, A.R.; Valeeva, F.G.; Tudriy, E. v.; Galkina, I. v.; Kataeva, O.N.; Zakharova, L.Y.; Latypov, S.K.; et al. Self-Assembling Systems Based on Amphiphilic Alkyltriphenylphosphonium Bromides: Elucidation of the Role of Head Group. *J. Colloid Interface Sci.* **2012**, *367*, 327–336, doi:10.1016/j.jcis.2011.10.074.

91. Escoula, B.; Hajjaji, N.; Lattes, A. A New Type of Water Insoluble Surfactant: Molecular Aggregation. *J. Chem. Soc. Chem. Commun.* **1984**, *18*, 1233–1234.
92. Prasad, M.; Moulik, S.P.; MacDonald, A.; Palepu, R. Self-Aggregation of Alkyl (C10-, C12-, C14-, and C16-) Triphenyl Phosphonium Bromides and Their 1:1 Molar Mixtures in Aqueous Medium: A Thermodynamic Study. *J. Phys. Chem. B* **2004**, *108*, 355–362, doi:10.1021/jp036358+.
93. Owoyomi, O.; Ige, J. Thermodynamics of Micellization of N-Alkyltriphenylphosphonium Bromides: A Conductometric Study. *Chem. Sci. J.* **2011**, *2*, 1–13, doi:10.4172/2150-3494.1000017.
94. Brown, P.; Butts, C.; Dyer, R.; Eastoe, J.; Grillo, I.; Guittard, F.; Rogers, S.; Heenan, R. Anionic Surfactants and Surfactant Ionic Liquids with Quaternary Ammonium Counterions. *Langmuir* **2011**, *27*, 4563–4571, doi:10.1021/la200387n.
95. Basu Ray, G.; Ghosh, S.; Moulik, S.P. Ternary Mixtures of Alkyltriphenylphosphonium Bromides (C12TPB, C14TPB and C16TPB) in Aqueous Medium: Their Interfacial, Bulk and Fluorescence Quenching Behaviour. *J. Chem. Sci.* **2010**, *122*, 109–117, doi:10.1007/s12039-010-0011-1.
96. Verma, S.K.; Ghosh, K.K. Micellar and Surface Properties of Some Monomeric Surfactants and a Gemini Cationic Surfactant. *J. Surfactants Deterg.* **2011**, *14*, 347–352, doi:10.1007/s11743-010-1237-0.
97. Gaynanova, G.A.; Vagapova, G.I.; Valeeva, F.G.; Vasilieva, E.A.; Galkina, I. v.; Zakharova, L.Y.; Sinyashin, O.G. A Novel Supramolecular Catalytic System Based on Amphiphilic Triphenylphosphonium Bromide for the Hydrolysis of Phosphorus Acid Esters. *Colloids Surf. A: Physicochem. Eng. Asp.* **2016**, *489*, 95–102, doi:10.1016/j.colsurfa.2015.10.032.
98. Mata, J.; Varade, D.; Bahadur, P. Aggregation Behavior of Quaternary Salt Based Cationic Surfactants. *Thermochim. Acta* **2005**, *428*, 147–155, doi:10.1016/j.tca.2004.11.009.
99. Basu Ray, G.; Chakraborty, I.; Ghosh, S.; Moulik, S.P.; Holgate, C.; Glenn, K.; Palepu, R.M. Studies on Binary and Ternary Amphiphile Combinations of Tetradecyltrimethylammonium Bromide (C14TAB), Tetradecyltriphenylphosphonium Bromide (C14TPB), and Tetradecylpyridinium Bromide (C14PB). A Critical Analysis of Their Interfacial and Bulk Behaviors. *J. Phys. Chem. B* **2007**, *111*, 9828–9837, doi:10.1021/jp073179.
100. Prasad, M.; Moulik, S.P.; Palepu, R. Self-Aggregation of Binary Mixtures of Alkyltriphenylphosphonium Bromides: A Critical Assessment in Favor of More than One Kind of Micelle Formation. *J. Colloid Interface Sci.* **2005**, *284*, 658–666, doi:10.1016/j.jcis.2004.10.063.
101. Bakshi, M.S.; Kaur, G.; Ahmad, I. Synergistic Interactions in Mixed Micelles of Alkyltriphenylphosphonium Bromides and Triblock Polymers. *Colloids Surf. A Physicochem. Eng. Asp.* **2005**, *253*, 1–8, doi:10.1016/j.colsurfa.2004.10.122.
102. Verma, S.K.; Ghosh, K.K.; Verma, R.; Xiang, W.; Li, N.; Zhao, X. Surface, Conformational and Catalytic Activity Approach of α-Chymotrypsin and Trypsin in Micellar Media. *Colloids Surf. A Physicochem. Eng. Asp.* **2015**, *470*, 188–193, doi:10.1016/j.colsurfa.2015.01.070.
103. Sehgal, P.; Kosaka, O.; Doe, H. Interfacial and Aggregation Properties of the Binary Mixture of Decanoyl-N-Methyl-Glucamide and Hexadecyltriphenylphosphonium Bromide. *Colloid Polym. Sci.* **2008**, *286*, 275–282, doi:10.1007/s00396-007-1766-x.
104. Dubey, N. Studies of Mixing Behavior of Cationic Surfactants. *Fluid Phase Equilibria* **2014**, *368*, 51–57, doi:10.1016/j.fluid.2014.02.007.
105. Benrraou, M.; Bales, B.L.; Zana, R. Effect of the Nature of the Counterion on the Properties of Anionic Surfactants. 1. Cmc, Ionization Degree at the Cmc and Aggregation Number of Micelles of Sodium, Cesium, Tetramethylammonium, Tetraethylammonium, Tetrapropylammonium, and Tetrabutylammoniu. *J. Phys. Chem. B* **2003**, *107*, 13432–13440, doi:10.1021/jp021714u.
106. Nakahara, Y.; Kida, T.; Nakatsuji, Y.; Akashi, M. New Fluorescence Method for the Determination of the Critical Micelle Concentration by Photosensitive Monoazacryptand Derivatives. *Langmuir* **2005**, *21*, 6688–6695, doi:10.1021/la050206j.
107. Chakraborty, A.; Saha, S.K.; Chakraborty, S. Effect of Size of Tetraalkylammonium Counterions on the Temperature Dependent Micellization of AOT in Aqueous Medium. *Colloid Polym. Sci.* **2008**, *286*, 927–934, doi:10.1007/s00396-008-1850-x.
108. Yu, Z.J.; Xu, G. Physicochemical Properties of Aqueous Mixtures of Tetrabutylammonium Bromide and Anionic Surfactants. 1. Temperature-Induced Micellar Growth and Cloud Point Phenomenon. *J. Phys. Chem.* **1989**, *93*, 7441–7445, doi:10.1021/j100358a037.
109. Rao, K.S.; Gehlot, P.S.; Gupta, H.; Drechsler, M.; Kumar, A. Sodium Bromide Induced Micelle to Vesicle Transitions of Newly Synthesized Anionic Surface Active Ionic Liquids Based on Dodecylbenzenesulfonate. *J. Phys. Chem. B* **2015**, *119*, 4263–4274, doi:10.1021/jp512805e.
110. Jiao, J.; Dong, B.; Zhang, H.; Zhao, Y.; Wang, X.; Wang, R.; Yu, L. Aggregation Behaviors of Dodecyl Sulfate-Based Anionic Surface Active Ionic Liquids in Water. *J. Phys. Chem. B* **2012**, *116*, 958–965, doi:10.1021/jp209276c.
111. Jin, Y.; Wang, L.; Wang, T.; Chen, P.; Bi, Y.; Yu, L. Aggregation Behavior of Dodecylsulfonate-Based Surface Active Ionic Liquids in Water. *J. Mol. Liq.* **2015**, *212*, 23–29, doi:10.1016/j.molliq.2015.08.049.
112. Rao, K.S.; Trivedi, T.J.; Kumar, A. Aqueous-Biamphiphilic Ionic Liquid Systems: Self-Assembly and Synthesis of Gold Nanocrystals/Microplates. *J. Phys. Chem. B* **2012**, *116*, 14363–14374, doi:10.1021/jp309717n.
113. Singh, T.; Rao, K.S.; Kumar, A. Effect of Ethylene Glycol and Its Derivatives on the Aggregation Behavior of an Ionic Liquid 1-Butyl-3-Methyl Imidazolium Octylsulfate in Aqueous Medium. *J. Phys. Chem. B* **2012**, *116*, 1612–1622, doi:10.1021/jp211537m.
114. Pal, A.; Yadav, A. Modulations in the Aggregation Behavior of Ionic Liquid 1-Butyl-3-Methylimidazolium Octylsulfate in Aqueous Alcohol Solutions. *J. Mol. Liq.* **2015**, *212*, 569–575, doi:10.1016/j.molliq.2015.10.009.

115. Thakkar, K.; Bharatiya, B.; Aswal, V.K.; Bahadur, P. Aggregation of 1-Alkyl-3-Methylimidazolium Octylsulphate Ionic Liquids and Their Interaction with Triton X-100 Micelles. *RSC Adv.* **2016**, *6*, 80585–80594, doi:10.1039/c6ra14664c.
116. Banjare, M.K.; Behera, K.; Satnami, M.L.; Pandey, S.; Ghosh, K.K. Supra-Molecular Inclusion Complexation of Ionic Liquid 1-Butyl-3-Methylimidazolium Octylsulphate with Α- and Β-Cyclodextrins. *Chem. Phys. Lett.* **2017**, *689*, 30–40, doi:10.1016/j.cplett.2017.09.033.
117. Singh, T.; Drechsler, M.; Müeller, A.H.E.; Mukhopadhyay, I.; Kumar, A. Micellar Transitions in the Aqueous Solutions of a Surfactant-like Ionic Liquid: 1-Butyl-3-Methylimidazolium Octylsulfate. *Phys. Chem. Chem. Phys.* **2010**, *12*, 11728–11735, doi:10.1039/c003855p.
118. Banjare, M.K.; Behera, K.; Banjare, R.K.; Sahu, R.; Sharma, S.; Pandey, S.; Satnami, M.L.; Ghosh, K.K. Interaction of Ionic Liquid with Silver Nanoparticles: Potential Application in Induced Structural Changes of Globular Proteins. *ACS Sustain. Chem. Eng.* **2019**, *7*, 11088–11100, doi:10.1021/acssuschemeng.8b06598.
119. Sen, S.; Paul, B.K.; Guchhait, N. Interaction of a Phenazinium-Based Photosensitizer with Surface Active Ionic Liquid Micelles: Investigating the Effect of Cyclodextrins on SAIL Micelles. *J. Mol. Liq.* **2019**, *274*, 584–591, doi:10.1016/j.molliq.2018.11.007.
120. Phani Kumar, B.V.N.; Reddy, R.R.; Pan, A.; Aswal, V.K.; Tsuchiya, K.; Prameela, G.K.S.; Abe, M.; Mandal, A.B.; Moulik, S.P. Physicochemical Understanding of Self-Aggregation and Microstructure of a Surface-Active Ionic Liquid [C4mim] [C8OSO3] Mixed with a Reverse Pluronic 10R5 (PO8EO22PO8). *ACS Omega* **2018**, *3*, 5155–5164, doi:10.1021/acsomega.8b00267.
121. Jiao, J.; Han, B.; Lin, M.; Cheng, N.; Yu, L.; Liu, M. Salt-Free Catanionic Surface Active Ionic Liquids 1-Alkyl-3-Methylimidazolium Alkylsulfate: Aggregation Behavior in Aqueous Solution. *J. Colloid Interface Sci.* **2013**, *412*, 24–30, doi:10.1016/j.jcis.2013.09.001.
122. Pal, A.; Yadav, S. Effect of Cationic Polyelectrolyte Poly(Diallyldimethylammonium Chloride) on Micellization Behavior of Anionic Surface Active Ionic Liquid 1-Butyl-3-Methylimidazolium Dodecylsulfate [C4mim][C12SO4] in Aqueous Solutions. *Colloid Polym. Sci.* **2018**, *296*, 1635–1650, doi:10.1007/s00396-018-4379-7.
123. Pal, A.; Punia, R. Self-Aggregation Behaviour of Cationic Surfactant Tetradecyltrimethylammonium Bromide and Bi-Amphiphilic Surface Active Ionic Liquid 3-Methyl-1-Pentylimidazolium Dodecylsulfate in Aqueous Solution. *J. Mol. Liq.* **2020**, *304*, 112803, doi:10.1016/j.molliq.2020.112803.
124. Pal, A.; Saini, M. Effect of Alkyl Chain on Micellization Properties of Dodecylbenzenesulfonate Based Surface Active Ionic Liquids Using Conductance, Surface Tension, and Spectroscopic Techniques. *J. Dispers. Sci. Technol.* **2020**, *41*, 547–556, doi:10.1080/01932691.2019.1593859.
125. Kaneshina, S.; Shibata, O.; Nakamura, M.; Tanaka, M. The Effect of Pressure on the Micelle Formation of Octyltrimethylammonium Octyl Sulfate and Its Homologous Surfactants. *Colloids Surf.* **1983**, *6*, 73–82, doi:10.1016/0166-6622(83)80008-0.
126. Pal, A.; Datta, S.; Aswal, V.K.; Bhattacharya, S. Small-Angle Neutron-Scattering Studies of Mixed Micellar Structures Made of Dimeric Surfactants Having Imidazolium and Ammonium Headgroups. *J. Phys. Chem. B* **2012**, *116*, 13239–13247, doi:10.1021/jp304700t.
127. Ao, M.Q.; Xu, G.Y.; Zhu, Y.Y.; Bai, Y. Synthesis and Properties of Ionic Liquid-Type Gemini Imidazolium Surfactants. *J. Colloid Interface Sci.* **2008**, *326*, 490–495, doi:10.1016/j.jcis.2008.06.048.
128. Liu, G.; Gu, D.; Liu, H.; Ding, W.; Li, Z. Enthalpy-Entropy Compensation of Ionic Liquid-Type Gemini Imidazolium Surfactants in Aqueous Solutions: A Free Energy Perturbation Study. *J. Colloid Interface Sci.* **2011**, *358*, 521–526, doi:10.1016/j.jcis.2011.03.064.
129. Maurya, J.K.; Khan, A.B.; Dohare, N.; Ali, A.; Kumar, A.; Patel, R. Effect of Aromatic Amino Acids on the Surface Properties of 1-Dodecyl-3-(4-(3-Dodecylimidazolidin-1-Yl)Butyl)Imidazolidine Bromide Gemini Surfactant. *J. Dispers. Sci. Technol.* **2018**, *39*, 174–180, doi:10.1080/01932691.2017.1306782.
130. Nacham, O.; Martín-Pérez, A.; Steyer, D.J.; Trujillo-Rodríguez, M.J.; Anderson, J.L.; Pino, V.; Afonso, A.M. Interfacial and Aggregation Behavior of Dicationic and Tricationic Ionic Liquid-Based Surfactants in Aqueous Solution. *Colloids Surf. A Physicochem. Eng. Asp.* **2015**, *469*, 224–234, doi:10.1016/j.colsurfa.2015.01.026.
131. Bhadani, A.; Singh, S. Synthesis and Properties of Thioether Spacer Containing Gemini Imidazolium Surfactants. *Langmuir* **2011**, *27*, 14033–14044, doi:10.1021/la202201r.
132. Kamboj, R.; Singh, S.; Bhadani, A.; Kataria, H.; Kaur, G. Gemini Imidazolium Surfactants: Synthesis and Their Biophysiochemical Study. *Langmuir* **2012**, *28*, 11969–11978, doi:10.1021/la300920p.
133. Tawfik, S.M. Simple One Step Synthesis of Gemini Cationic Surfactant-Based Ionic Liquids: Physicochemical, Surface Properties and Biological Activity. *J. Mol. Liq.* **2015**, *209*, 320–326, doi:10.1016/j.molliq.2015.05.054.
134. Zhang, S.; Yan, H.; Zhao, M.; Zheng, L. Aggregation Behavior of Gemini Pyrrolidine-Based Ionic Liquids 1,1′-(Butane-1,4-Diyl)Bis(1-Alkylpyrrolidinium) Bromide ([C Npy-4-C Npy][Br 2]) in Aqueous Solution. *J. Colloid Interface Sci.* **2012**, *372*, 52–57, doi:10.1016/j.jcis.2012.01.040.
